# Screening for thyroid dysfunction and treatment of screen-detected thyroid dysfunction in asymptomatic, community-dwelling adults: a systematic review

**DOI:** 10.1186/s13643-019-1181-7

**Published:** 2019-11-18

**Authors:** Francesca Reyes Domingo, Marc T. Avey, Marion Doull

**Affiliations:** 1 Public Health Agency of Canada, 130 Colonnade Road, Ottawa, Ontario Canada; 2 Public Health Agency of Canada, 785 Carling Ave, Ottawa, Ontario Canada

**Keywords:** Thyroid dysfunction, Hypothyroidism, Hyperthyroidism, Screening, Treatment

## Abstract

**Background:**

This systematic review was conducted to inform the Canadian Task Force on Preventive Health Care recommendations on screening for thyroid dysfunction (TD). The review sought to answer key questions on the benefits and harms of screening for TD, patients’ values and preferences for screening, and the benefits and harms of treating screen-detected TD.

**Methods:**

This review followed Canadian Task Force on Preventive Health Care methods, which include the Grading of Recommendations Assessment, Development, and Evaluation (GRADE) approach. The search strategy used for benefits and harms of screening and treatment was an update to the 2014 review by the US Preventive Services Task Force and searched MEDLINE and the Cochrane Library. MEDLINE, Embase, ProQuest Public Health, and SCOPUS were searched for patients’ values and preferences for screening. Outcomes of interest included all-cause mortality, deaths due to cardiovascular diseases, fatal and non-fatal cardiovascular events, atrial fibrillation, fractures, quality of life, cognitive function, and harms due to TD treatment. Two reviewers independently screened abstracts and full texts according to pre-determined inclusion criteria and assessed the risk of bias for each study included. Strength and quality of the evidence was assessed for each outcome. A narrative synthesis was conducted due to heterogeneity of the included studies.

**Results:**

No studies were found on screening for TD, treatment of subclinical hyperthyroidism, or patients’ values and preferences for screening for TD. Twenty-two studies (from 24 publications) on the treatment of TD in patients with screen-detected subclinical hypothyroidism were included. Results from the included randomized controlled trials suggested no benefit of treatment for subclinical hypothyroidism for the large majority of outcomes. We found very low-quality evidence (from two cohort studies) for a small reduction in all-cause mortality among adults < 65 or 40–70 years who were treated for TD compared to those who were not.

**Conclusions:**

This review found moderate to very low-quality evidence on the benefits and harms of treatment for subclinical hypothyroidism, with most of the evidence showing no benefit of treatment.

## Background

### Purpose

The purpose of this review is to synthesize the evidence on the effects of screening and subsequent treatment for thyroid dysfunction (TD) in asymptomatic, non-pregnant, community-dwelling adults to inform the development of a Canadian Task Force on Preventive Health Care (Task Force) guideline on screening for TD. Screening for TD may identify asymptomatic subclinical TD or overt TD in cases where symptoms are not recognized as symptoms of TD or are not reported.

### Definition

TD is an impairment in the function of the thyroid gland and comprises a spectrum of disorders ranging from asymptomatic disorders to symptomatic thyroid disease. Hypothyroidism is a condition where there is too little thyroid hormone present in the bloodstream, because the thyroid gland is either unable to produce sufficient thyroid hormones or is absent (athyreosis); while hyperthyroidism results when there is too much thyroid hormone present in the bloodstream resulting from an overactive thyroid gland [1, 2].

Onset of hypothyroidism is typically slow and often includes fatigue/tiredness, dry skin, sensitivity to cold, hair loss, weight gain, constipation, voice changes, and slowed movements and thoughts [1, 3–5]. Hyperthyroidism may develop gradually or suddenly and may include tachycardia, fatigue, weight loss, intolerance to heat, increased sweating, tremor, and hyperactive reflexes [2, 6].

TD is defined as either subclinical or overt, based on laboratory findings [1, 2, 4, 5, 7–10]. Overt hypothyroidism is characterized by elevated serum thyroid-stimulating hormone (TSH), also known as thyrotropin, and subnormal free thyroxine levels (T_4_) while subclinical hypothyroidism is characterized by elevated serum TSH levels and normal free T_4_ levels [1, 4, 5, 11]. Overt hyperthyroidism is defined as subnormal serum TSH levels and elevated free serum triiodothyronine (T_3_) or free T_4_ levels while subclinical hyperthyroidism is characterized by subnormal serum TSH levels and normal serum free T_3_ and free T_4_ levels [5, 12, 13]. In the context of TD, the term “subclinical” is used to refer to the laboratory parameters above; it is possible (though less common) for symptoms to occur when subclinical hyper or hypothyroidism is present. Similarly, the term “overt” refers to the laboratory parameters above; the symptoms of overt TD are often non-specific and may be unrecognized and unreported by patients.

The normal reference range for TSH varies depending on the laboratory and/or the reference population surveyed, and the range may widen with increasing age [5, 11, 13]. Currently, no single Canadian reference standard for normal TSH range is available. As such, clinicians refer to the normal reference ranges provided by their provincial health ministries or laboratories [14–18] or refer to guidelines produced by other organizations [5, 11, 13, 19, 20]. Canadian sources report upper limits of normal TSH in adults ranging from 4.0 to 5.5 milliunits per liter (mU/L) [14–20], with one source recommending an upper limit of 6.0 mU/L in older adults (> 65 years of age) [19]. Lower limits of normal TSH in adults range from 0.20 to 0.45 mU/L [14–17, 19, 20]. Hence estimates for the prevalence of TD would vary depending on the TSH reference ranges used, which may potentially lead to an over-estimation in the adult population ≥ 60 years of age, if age-specific TSH ranges are appropriate and were not used [21, 22].

### Prevalence and burden of TD

Very few studies have reported on the prevalence of TD in Canada. The 2008–2009 Canadian Community Health Survey on Healthy Aging surveyed a representative sample of the Canadian population ≥ 45 years of age living in the 10 provinces with an overall combined (household and person) response rate of 74.4% [23]. That survey found that 10% of respondents reported that they had been diagnosed by a health professional as having a thyroid condition that was expected to last, or had already lasted, 6 months, or more. The rate was higher in females compared to males (16% vs. 4%) and also increased with age: 9% in adults 45–64 years of age, 14% in adults 65–84 years of age, and 16% in those ≥ 85 years [23]. The 2005 Canadian Community Health Survey included a representative sample of Canadians 12 years of age and older living in households in all provinces and territories with an overall combined (household and person) response rate of 78.9%. Only 6% of respondents self-reported that they had a thyroid condition that was diagnosed by a health professional (no definition was provided) [24]. Given the different populations surveyed, these estimates are consistent. Both the 2008–2009 and 2005 Canadian Community Health Surveys excluded persons living on reserves, in other Aboriginal settlements and residents of certain remote regions, full-time members of the Canadian Armed Forces, and institutionalized residents [23, 24].

One Canadian study estimated the prevalence and incidence of autoimmune thyroid disease in adults ≥ 20 years of age living in Manitoba by examining hospital, physician, and/or prescription claims suggestive of autoimmune thyroid disease in the previous 5 years among 20,940 people [25]. The study found that the 2005 age-adjusted prevalence of autoimmune thyroid disease in the general population was 9% (95% confidence interval (CI) of 8–11). Prevalence increased with age and was higher in women than in men. The study also estimated that the age-adjusted incidence of autoimmune thyroid disease per 100,000 persons was 398 new cases annually (95% CI 299–497) [25].

It has been estimated that TD affects approximately 5% of people living in the USA [26]. Studies from the USA and UK report prevalence rates in adults between 4 and 10% for subclinical hypothyroidism and between 1 and 2% for subclinical hyperthyroidism [27]. Studies consistently report higher prevalence of TD in women compared to men and higher rates in older (> 60 years) compared to younger adults [27, 28]. The prevalence of TD differs between areas with low versus sufficient iodine consumption. In iodine-replete areas (such as Canada), the prevalence of overt hypothyroidism ranges from 1 to 2%, and overt hyperthyroidism is between 0.5 and 2% [28].

### Etiology and natural history

In iodine-replete areas, the most common cause of hypothyroidism is Hashimoto’s thyroiditis or chronic autoimmune thyroiditis [28]. Hypothyroidism may also be caused by other autoimmune diseases, congenital anomalies, iodine deficiency, infiltrative diseases, surgical removal of all or part of the thyroid gland, radiation treatment to the thyroid gland or around the head and neck area, and by taking medications that can alter thyroid levels (i.e., amiodarone, lithium) [1, 11].

The most common cause of hyperthyroidism in iodine-replete areas is Graves’ disease, which is an autoimmune disorder that causes the thyroid gland to release too much thyroid hormone [13]. Other common causes of hyperthyroidism include toxic multinodular goiter, toxic adenoma, and painless thyroiditis and less common causes include drug-induced thyroiditis, pregnancy-induced, and post-partum-induced thyroiditis [2, 29].

The annual risk of progression to overt disease is 2–6% for those with subclinical hypothyroidism [30] and 1–2% for those with subclinical hyperthyroidism [29]. One study reported that 38% of patients with elevated serum TSH levels and 52% of those with subnormal serum TSH levels spontaneously reverted to euthyroidism (i.e., TSH levels within normal range) without intervention over a 60-month period [31]. Another study reported that 37% of the subclinical hypothyroidism patients in the study subsequently showed normal TSH levels without the use of treatment over a period of 6–72 months (mean 31.7 months) [32].

### Risk factors

Individuals at increased risk of TD include females, older adults (> 60 years of age), those with a previous personal history of or strong family history of thyroid disease, and post-partum women [5, 20, 28]. Individuals at increased risk for hypothyroidism include patients with other autoimmune diseases, goiter, previous hyperthyroidism, and those who have had previous surgery or radiation therapy on the thyroid gland or head and neck area [5, 20]. Those at increased risk for hyperthyroidism include individuals receiving drug therapies that affect thyroid levels such as lithium and amiodarone, and those with low iodine intake [5, 20].

### Interventions/treatments

Appropriate clinical history and examination of the patient, including an assessment of the cause and severity of the TD, is recommended prior to initiation of treatment.

Thyroid hormone replacement with l-thyroxine monotherapy is used to treat hypothyroidism [11]. Adverse drug reactions are usually the result of taking too much l-thyroxine causing the person to develop symptoms of hyperthyroidism that may include nervousness, palpitations, atrial fibrillation, heart failure, exacerbation of angina pectoris, weight loss, and decreased bone mineral density leading to an increased risk of fractures [26, 33]. Treatment is usually recommended for overt hypothyroid patients or in subclinical hypothyroid patients with TSH levels > 10.0 mIU/L. Treatment of individuals with elevated TSH levels but < 10.0 mIU/L is considered based on the clinical status of the patient or if the patient presents with symptoms suggestive of hypothyroidism [11, 20].

Treatment of hyperthyroidism may include antithyroid drugs (e.g., methimazole, propylthiouracil), radioactive iodine ablation, and thyroidectomy [2, 13]. Antithyroid drugs may cause rashes, jaundice, arthralgia, nausea, abdominal pain, fatigue, pale stools or dark urine, fever, vomiting, or sore throat [2, 13]. Rare but serious side effects may include agranulocytosis, vasculitis, or hepatic damage [13]. Radioactive iodine ablation may lead to permanent hypothyroidism requiring lifelong thyroid hormone replacement therapy [2]. Complications from surgical removal of the thyroid gland include complications from damage to the surrounding parathyroid glands and recurrent or superior laryngeal nerves, hypocalcemia due to hypoparathyroidism, postoperative bleeding, and complications from general anesthesia [2, 13].

### Consequences if left untreated

Between 37 and 38% of individuals with elevated TSH levels [31, 32] and 52% of individuals with subnormal TSH levels will become euthyroid without treatment over 5–6 years [31]. For non-pregnant adults, untreated hypothyroidism may increase the risk of developing cardiac dysfunction, hypertension, dyslipidemia, cognitive impairment, neuromuscular dysfunction, neuropsychiatric symptoms, and infertility [1, 29]. Untreated hyperthyroidism may increase the risk of adverse cardiac events (e.g., atrial fibrillation, cardiac dysfunction, heart failure), systemic and neuropsychiatric symptoms, reduced bone mineral density and fractures [2, 29], and, in rare cases, a life-threatening condition called thyroid storm (which may include symptoms such as tachycardia, fever, nausea/vomiting, delirium, and extreme lethargy) [2].

### Considerations for screening

Screening tests are performed on asymptomatic individuals to identify a disease or risk factor at an early or unrecognized stage in order to offer interventions that may lead to better health outcomes sooner compared to treatment at a later stage after symptoms are recognized [34].

Screening for TD can identify both patients with asymptomatic subclinical TD, as well as those with unrecognized or undiagnosed overt TD. An initial blood test to measure serum levels of TSH can be used to screen for TD. It may be followed up with additional blood tests to measure free T_4_/free T_3_ levels if TSH levels are abnormal. However, there is uncertainty over what the appropriate reference ranges are for TSH test results. At present, TSH reference ranges in Canada are not adjusted for age. As well, to date, those > 70 years of age have been shown to have fewer symptoms and less benefit from treatment compared to younger adults [11, 30].

Potential harms from screening include overdiagnosis and overtreatment that can lead to negative health outcomes and additional costs to the health care system [35]. A diagnosis of disease may also be associated with psychological consequences that may impair a patient’s quality of life (QoL), which has been called the “labelling effect” [36]. Two studies looked at the health-related QoL of subjects with abnormal TSH values or of women with subclinical thyroid disease who were not aware of their health status before answering a health-related QoL questionnaire [37, 38]. Both studies found that a poor health-related QoL score was not related to the abnormalities in subjects’ TSH or thyroid hormone levels, but perhaps could partly be explained by the labelling effect phenomenon.

### Current clinical practice

In Canada, no formal screening programs for TD in adults exist, but research suggests that TSH tests are potentially overused in clinical practice. One Canadian study, which predominantly included patients from urban areas in Ontario, reported that 71% of patients without thyroid disease and not on thyroid medications had at least one TSH test recorded in their chart in the previous 2 years [39, 40]. The study also found high variability in TSH testing among family practices, with practices testing between 25 and 100% of all adult patients and a trend towards more testing in large practices [39].

Canadian data on the number of individuals being treated for TD is lacking. However, a 2015 report by the Canadian Institute for Health Information indicated that the rates of thyroid hormone use among active beneficiaries^1^ of provincial public drug programs (excluding Quebec) for thyroid hormones ranged from 5 to 22% [42].

### Previous review and Canadian Task Force on Preventive Health Care Recommendations

In 1990, the Task Force, previously known as the Canadian Task Force on the Periodic Health Examination, developed recommendations on the early detection of hyperthyroidism and hypothyroidism among asymptomatic individuals [43]. At that time, the Task Force found fair evidence to exclude serum TSH test from periodic health examination for the early detection of hyperthyroidism in asymptomatic individuals. They also found insufficient evidence to support the inclusion of TSH screening for hypothyroidism among asymptomatic people, particularly in those ≥ 75 years of age. The Task Force did, however, recommend maintaining a high index of suspicion for hypothyroidism in post-menopausal women given the high prevalence in that group. Although the evidence used to inform those recommendations was obtained using standardized methods for evaluating and weighing scientific evidence, it was not based on a systematic review of the literature. The Task Force has not issued any recommendations since then.

## Methods

This review was completed according to Task Force methods [44], which are based on the Cochrane Handbook for Systematic Reviews of Interventions [45] and the Grading of Recommendations Assessment, Development and Evaluation (GRADE) methods [46]. The review and abstract are reported according to the Preferred Reporting Items for Systematic Reviews and Meta-Analyses (PRISMA) guidelines and the PRISMA checklist [47, 48]. A protocol was developed a priori and registered with the International Prospective Register of Systematic Reviews [49] (protocol registration number CRD42016033622). Any amendments to the protocol are outlined in this report.

### Analytic framework, review approach, and key questions

**Fig. 1:**
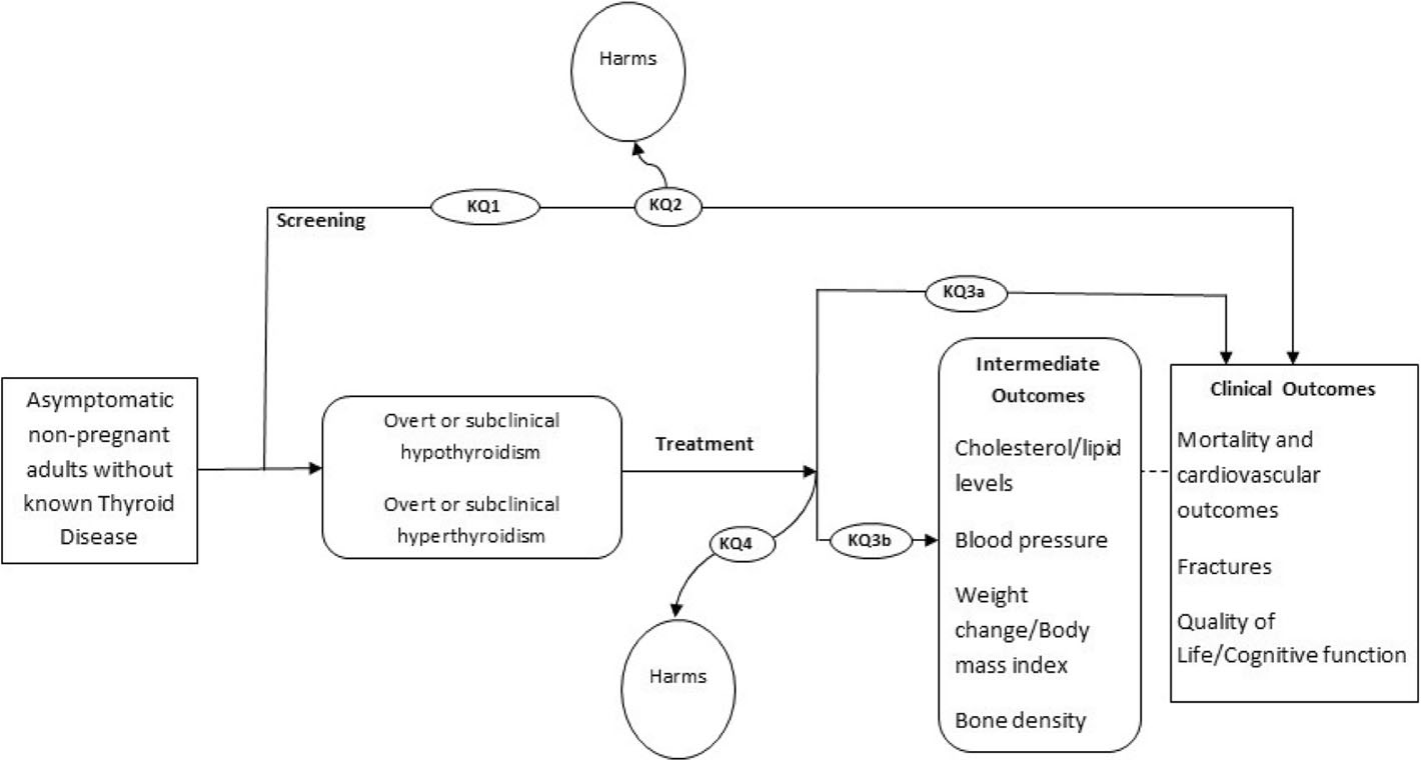
Analytic framework

The analytic framework for this review is presented in Fig. 1 and was adapted from the 2014 US Preventive Services Task Force (USPSTF) review on screening for TD [50]. Because no studies on TD screening were identified in previous reviews [26, 50], key questions (KQs) on clinical benefits and harms of subsequent treatment for TD among screen-detected individuals were retained in the framework to use as linked evidence. The framework presents KQs and outcomes examined for this review.

Key questions (KQ) are as follows:

- KQ1: Does screening asymptomatic, non-pregnant adults for TD reduce morbidity and mortality?
- KQ2: What are the harms of screening asymptomatic, non-pregnant adults for TD?
- KQ3: Does treatment of screen-detected overt or subclinical TD improve (a) morbidity or mortality or (b) intermediate outcomes?
- KQ4: What are the harms of treating screen-detected TD in asymptomatic, non-pregnant adults?
- KQ5: What are asymptomatic, non-pregnant adults’ preferences and values concerning screening for TD?
- KQ6: If screening asymptomatic, non-pregnant adults for TD is clinically effective, then what is the cost-effectiveness and associated resource use?

### Search strategy

The search strategy for key questions 1 to 4 (KQ1-4) was an updated search from the 2014 review on TD completed for the USPSTF [50]. The update included a search for published literature from the end date of the original USPSTF review search (July 2014) to July 25, 2018. The search was limited to English language articles using the following databases: Cochrane Library, Ovid MEDLINE(R), Ovid MEDLINE(R) Daily, Ovid MEDLINE(R) In-Process & Other Non-Indexed Citations, and Ovid OLDMEDLINE(R) (see Additional file 1).

In addition, to enhance the search for relevant literature for KQ1-4, a forward citation search on the 17 included studies from the USPSTF review [50] was conducted using the SCOPUS database on July 25, 2018 (see Additional file 1). The list of excluded studies from the USPSTF review (that were excluded based on wrong outcome or population) and the lists of primary studies from systematic reviews that passed full-text screening were manually searched for additional relevant literature. All of the studies included in USPSTF systematic review [50] were screened to ensure they met all of the inclusion and exclusion criteria for this review.

For KQ5 on patient values and preferences for screening, English and French language literature from time of database inception to July 25, 2018, were searched using the same OVID databases used to search KQ1-4 as well as Embase, ProQuest Public Health, and SCOPUS. The search strategy for KQ5 can be found in Additional file 1. A search for KQ6 (cost-effectiveness) of screening was not initiated as evidence on the effectiveness of screening was not found (KQ1-2). The search updates (KQ1-4), forward citation searches, and search for KQ5 were conducted by Health Canada research librarians.

### Eligibility criteria

Studies were eligible for KQ1-2 if they included and analyzed data for outcomes of patients screened for TD. Studies eligible for KQ3-4 could have applied a screening test as part of study eligibility, but only examined outcomes related to treating or not treating TD. Studies examining the effects of screening for TD or treating screen-detected TD among asymptomatic non-pregnant community-dwelling adults (age ≥ 18 years) were included. Included studies involved patients without a history of thyroid disease (though studies of patients with Hashimoto’s thyroiditis, subclinical hypothyroidism or subclinical hyperthyroidism could have been included as long as patients were not clearly symptomatic). Studies involving patients with uninvestigated non-specific symptoms (e.g., fatigue, weight gain) and studies that did not clearly describe enrolment of symptomatic patients were also included. Studies involving > 20% of patients who were hospitalized or were recently hospitalized (in the past month), or were undergoing treatment with medications that may alter thyroid levels, such as lithium, amiodarone, radiation, and chemotherapy were excluded.

The TD screening intervention of interest had to include a TSH measurement and the treatment interventions of interest included thyroid hormone replacement (e.g., levothyroxine), antithyroid medications (e.g., methimazole), ablation therapy (e.g., radioactive iodine), and/or surgery. The comparison was screening versus no screening for questions on screening effectiveness and patient values and preferences (KQ1-2 and KQ5). For questions on treatment effectiveness (KQ3-4), populations that were screened positive for TD and then treated compared to those that were screened positive and not treated (either placebo or observation) were included. Studies were not excluded based on the type of treatment provided, treatment dose or the duration of treatment.

The clinical outcomes of interest for KQ1 and KQ3a were mortality (all-cause and cardiovascular related), fatal and non-fatal cardiovascular events, atrial fibrillation, fractures, QoL, and cognitive function. The intermediate outcomes of interest for KQ3b were cholesterol and lipid levels, blood pressure, body mass index (BMI) or weight change, and bone density. Harms of screening (i.e., psychological effects, harms of workup, overdiagnosis, and overtreatment) and harms due to treatment were the outcomes of interest for KQ2 and KQ4, respectively. For KQ5, outcomes were patient values and preferences towards screening and for KQ6, cost-effectiveness analysis of screening. The outcome definitions used in this review are provided in Additional file 1. For KQs 1–4, we included study designs that evaluated the comparative effectiveness of screening vs. no screening or treatment vs. placebo/observation. Since we were interested in both the benefit of screening or treatment (KQ 1 and 3) and harms (KQ 2 and 4), we included randomized controlled trials (RCTs) as well as controlled observational studies when RCT evidence was not available. For KQs 5 and 6, our criteria for study design was broader since we were interested in patient preferences and values (e.g., descriptive, mixed-methods studies), and economic studies that evaluated cost-effectiveness (e.g., RCTs, modeling studies) which are unlikely to be captured in RCTs and observational studies alone.

### Study selection

Two reviewers independently screened all abstracts and full texts from the database searches using predetermined inclusion criteria. In cases of disagreement that could not be resolved by discussion, a third reviewer was consulted. DistillerSR [51] online software interface was used to document the screening and full-text review process.

A staged approach was used to identify the source of evidence for each outcome for KQ1-4, starting with study type providing the highest quality evidence—RCTs—followed by controlled observational studies (i.e., controlled observational studies were only included for outcomes/populations not already addressed via RCT evidence).

### Rating of outcomes

Outcomes for KQ1-4 were rated independently by the 4 members of the Task Force TD working group as per the GRADE approach [46]. Studies reporting on outcomes rated as critical or important were considered for inclusion in this review. The final outcome ratings are provided in Additional file 1. As per Task Force methods [44], the TD working group discussed and agreed on nine critical or important outcomes for consideration in the guideline on screening for TD. The nine outcomes were: all-cause mortality, deaths due to cardiovascular diseases, fatal and non-fatal cardiovascular events, atrial fibrillation, fractures, cognitive function, thyroid-specific QoL, fatigue/tiredness, and harms due to TD treatment.

### Data extraction

One reviewer extracted relevant information on study characteristics (e.g., study design, setting, sample size, population), and a second reviewer extracted study results. A third reviewer independently verified the accuracy and completeness of the entire data extraction. For all outcomes, unadjusted values and intention-to-treat data were extracted where possible. If unadjusted values were not published, the adjusted values were included in the narrative review. For included studies with several publications, data from all sources were extracted with the intent of using the most directly applicable or appropriate data for each outcome only. When required, study authors were contacted for further information or data (see Additional file 1).

### Data synthesis

Due to the clinical and methodological heterogeneity of the included studies (i.e., varying treatment dosages and duration of treatment and follow-up) a meta-analysis was not completed, and results were summarized narratively. Results from RCTs and observational studies were synthesized and reported separately.

When possible, outcome differences between the treatment and control groups were reported as measured in the included studies (i.e., hazard ratios or incidence rate ratios for dichotomous outcomes and mean difference (MD) at follow-up or difference in mean change from baseline to follow-up for continuous outcomes). However, if outcome differences between the treatment and control groups were not provided in the studies, differences between groups were calculated using RevMan (i.e., MD for continuous outcomes or odds ratios (ORs) for dichotomous outcomes) [52]. Where appropriate or if sufficient data were available, absolute values were calculated using GRADEPro [53]. For consistency and ease of interpretation, values reported as mg/dL were converted to mmol/L. Results were stratified by age group and sex for mortality and cardiovascular outcomes where available based on the published data.

When an outcome was measured at multiple follow-up points in an RCT, the follow-up point that was the most similar to the time points used by the other studies for the outcome being synthesized was used.

### Risk of bias/quality ratings for individual studies

Two reviewers independently assessed risk of bias for each RCT using the Cochrane Risk of Bias Tool [54]. The Newcastle-Ottawa Scale was used to assess the quality rating for each of the observational studies [55]. A third reviewer was consulted in cases of unresolved conflicts. A separate independent reviewer checked all of the risk of bias and quality assessments to ensure accuracy. Reviewers also assessed the influence of the source of funding for each of the included studies.

### Assessment of the overall quality (or certainty) of the evidence for each outcome using GRADE

Two reviewers assessed the strength and certainty of the body of evidence for the outcomes using the GRADE approach [46]. A third reviewer was consulted in cases of disagreement that could not be resolved by discussion.

GRADE domains were assessed in the following manner for outcomes reported narratively and is consistent with previously published guidance [56]:

1. Risk of bias: Based on the risk of bias assessments for individual studies, a judgment about the overall risk of bias (across all studies) by each outcome was made using GRADE and reflects how likely or unlikely the intervention effects for that particular outcome were affected by bias.
2. Inconsistency: To assess inconsistency, the individual study point estimates and the CIs were considered. If the point estimates were close together and the CIs overlapped, then the outcome was not downgraded for inconsistency.
3. Indirectness: To assess indirectness, the applicability of the evidence to the guideline research question was considered (i.e., differences in population, intervention, and outcome measures (use of surrogate outcomes or indirect comparisons)).
4. Imprecision: To assess imprecision in systematic reviews conducted for guideline development, clinical thresholds between recommending and not recommending the intervention need to be considered. If the effect estimates in the majority of the studies cross the clinical threshold, then the outcome would be downgraded for imprecision. If the clinical decision threshold was not crossed, or could not be established, the optimal information size criterion of the body of evidence was considered only when the effect sizes seemed implausibly large and the sample size across studies was small. If the number of participants/events across studies did not meet the optimal information size, then the outcome was downgraded for imprecision.
5. Publication bias: Factors that may lead to suspected publication bias were considered and assessed: inclusion of mostly small studies, non-comprehensive search strategy, and inclusion of very few studies with negative or null findings.

A priori clinical decision thresholds could not be established after consulting with the TD working group and clinical experts. Internet and literature searches were conducted to find additional information on clinically important thresholds. A summary of the clinical decision thresholds used to assess imprecision is provided in Additional file 1.

GRADE terminology [46] was used to summarize the quality of the overall body of evidence for each outcome: the term “high certainty” was used for high-quality evidence, “moderate certainty” for moderate-quality evidence, “may/may not” for low-quality evidence, and “large uncertainty” for very low-quality evidence.

### Changes to protocol

In the original protocol, thyroid cancer was identified as an outcome of interest; however, it was not considered in the systematic review because thyroid screening tests do not detect thyroid cancer, and the majority of thyroid cancers will have normal thyroid function at the time of diagnosis.

## Results

**Fig. 2:**
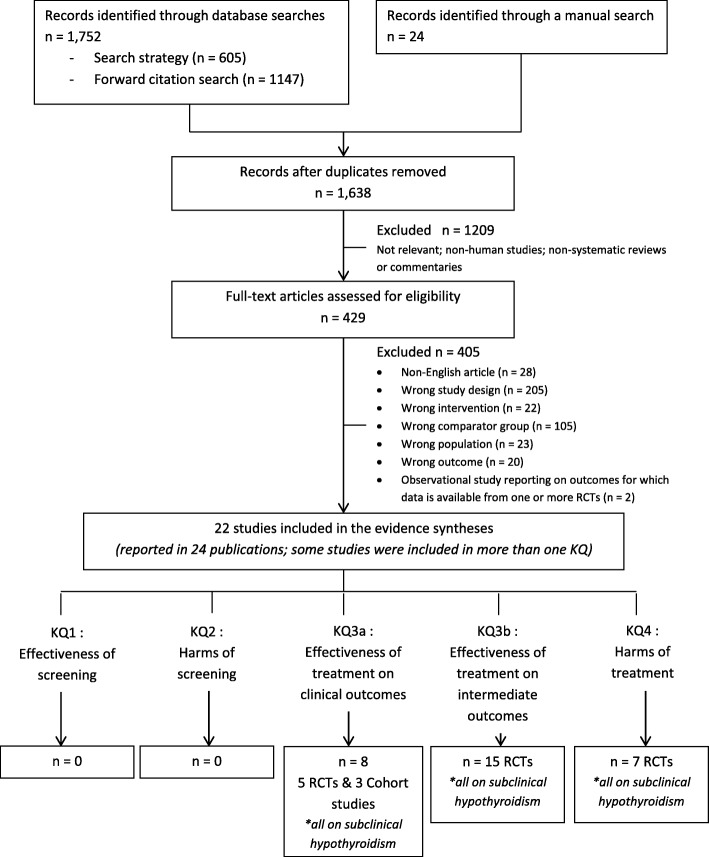
PRISMA flowchart—summary of evidence search for the benefits and harms of screening and treatment for thyroid dysfunction (KQ1-4)

**Fig. 3:**
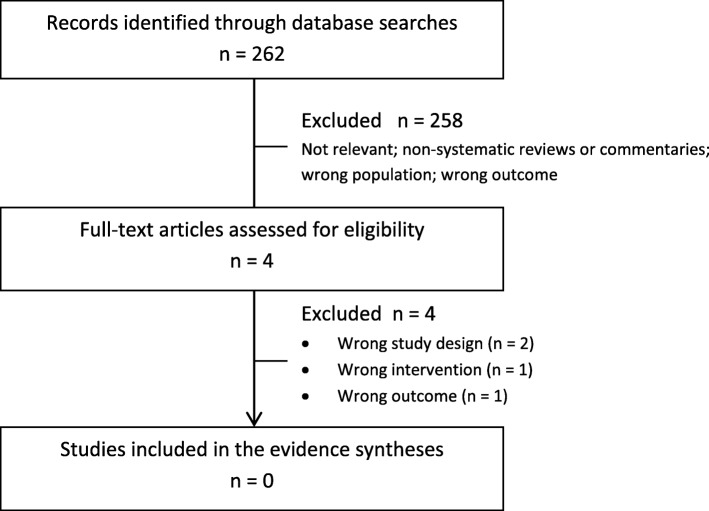
PRISMA flowchart—summary of evidence search for patient’s preferences and values towards screening for thyroid dysfunction (KQ5)

The literature search identified 1638 unique citations for the benefits and harms of screening (KQ1-2) and treatment (KQ3-4) of TD and 262 unique citations on patient’s values and preferences towards screening for TD (KQ5) for which 429 and 4 full-text articles, respectively, were assessed for eligibility (see Figs. 2 and 3 for PRISMA flowcharts). The list of studies excluded in the full-text review for KQ1-5 can be found in Additional file 1. No studies that reported on the effectiveness or harms of screening asymptomatic, non-pregnant adults for TD (KQ1-2) or on patient’s preferences and values towards screening (KQ5) were eligible for inclusion.

Twenty-two studies (reported in 24 publications) met the inclusion criteria for the key questions on the effectiveness (KQ3) or harms (KQ4) of treatment for TD (19 RCTs in 21 publications [57–77] and three cohort studies [78–80]). See Additional file 1 for study characteristics. Two publications from Iqbal et al. [62] and Jorde et al. [63] described the same trial population but reported on different outcomes, while two publications from Teixeira et al. [74, 75] reported on the same trial but at two different follow-up points. All of the included studies assessed the effects of treatment for screen-detected subclinical hypothyroidism. No studies reporting on the benefits or harms of treatment for subclinical hyperthyroidism or asymptomatic overt hypothyroidism or hyperthyroidism were found.

Eight RCTs [58, 59, 61–63, 67, 68, 71, 73] and three cohort studies [78–80] were conducted in Europe, four RCTs [60, 66, 70, 76] in the Middle East, four RCTs [57, 65, 72, 74, 75] in South America, and three RCTs [64, 69, 77] in Asia. The majority of studies were conducted in non-specified outpatient clinics, but a few were conducted in primary care clinics (one RCT [71] and two cohort studies [78, 80]), specialized outpatient clinics (four RCTs [63, 65, 67, 76];), or in both primary care and hospital outpatient clinics (one cohort study [79];).

The included studies enrolled asymptomatic non-pregnant adults and compared treatment for subclinical hypothyroidism with levothyroxine versus placebo (14 RCTs) [58, 59, 62–64, 66–76], or with levothyroxine versus no treatment (five RCTs [57, 60, 61, 65, 77] and three cohort studies [78–80]). No included RCTs or cohort studies reported on other interventions of interest (e.g., use of antithyroid medications, ablation therapy and surgery). The majority of studies (14 RCTs [57–60, 64–68, 70, 72, 74–77] and one cohort study) [78] had participants with mean ages of < 60 years. All but one RCT [60] included participants with mean TSH levels at baseline between 4.65–8.47 mIU/L. The majority of the studies (16 RCTs [57–61, 64–70, 72, 74–77] and two cohort studies [78–80]) had predominantly female participants (i.e., > 70% female). In all but one RCT [70], treatment dosage varied depending on patient baseline or follow-up TSH levels.

Three RCTs [62, 63, 71, 77] included participants who were recruited and screened positive through population-based screening. One RCT [77] from China invited all registered Chinese residents of Ningyang county who were ≥ 40 years of age to undergo a thyroid screening test. One RCT in the UK [71] recruited individuals who were participating in a community-based cross-sectional study looking at the prevalence of TD. One RCT from Norway [62, 63] included participants from the municipality of Tromsø who were recruited as part of a general health survey where recruitment included sending out invitations to whole birth cohorts and to those identified via random sampling. The rest of the RCTs and cohort studies included in this review included participants who had undergone a TSH screening test, screened positive and then were treated, but the TSH test was not administered via a population-based screening approach.

The duration of follow-up (either from start of treatment/placebo or from achievement of euthyroidism) for RCTs ranged from 3 to 36 months: 9 RCTs [58, 59, 61, 65, 67–70, 72] had follow-up durations of 6 months or less and 10 RCTs from 8 months to 3 years [57, 60, 62–64, 66, 71, 73, 74, 76, 77]. Registry-based time-to-event data from the retrospective cohorts were collected over a period of 8–14 years with median follow-up periods of 5.0–7.6 years. In one cohort study [80], 94% of patients in the treatment group continued to take levothyroxine during the 7.6 year follow-up period. Median treatment duration for those in the treatment groups were 3.6 years [79] and 3.7 years [78] in the other cohort studies. Most of the RCTs had low risk of bias for blinding of participants and study personnel [57–60, 62–64, 66–69, 71–76], a little over half had low risk of bias for blinding of outcome assessment [57–60, 62, 63, 66, 68, 71, 73–75], and the majority had unclear risk of bias for the other domains that were assessed. A majority of the RCTs had small sample sizes (< 100 participants) [57–63, 65, 67–72, 74–76]. All three cohort studies [78–80] scored well (8/9 points) on the Newcastle-Ottawa Scale; sample sizes ranged from 1192 to 12,212 participants.

Details on the characteristics of the individual RCTs and cohort studies, individual study results and risk of bias or quality assessments can be found in Additional file 1.

***KQ1: Does screening asymptomatic, non-pregnant adults for TD reduce morbidity and mortality?***

No studies reporting on the effects of screening asymptomatic, non-pregnant adults for TD on morbidity and mortality were found.

***KQ2: What are the harms of screening asymptomatic, non-pregnant adults for TD?***

We found no studies reporting on the harms of screening asymptomatic, non-pregnant adults for TD.

***KQ3a: Does treatment of screen-detected overt or subclinical TD improve morbidity or mortality?***

The summary of the findings is described below. Further details on the evidence, including summary of findings tables and GRADE evidence profile tables for outcomes for KQ3a can be found in Additional file 2: Evidence Set 1. Characteristics of the individual RCTs and cohort studies, individual study results and risk of bias/quality assessments can be found in Additional file 1.

### Mortality, cardiovascular events, and fractures

An RCT at low risk of bias by Stott et al. [73] and three cohort studies by Andersen et al. [78], Andersen et al. [79], and Razvi et al. [80] with high-quality ratings reported on the effects of treatment versus no treatment for subclinical hypothyroidism on outcomes of mortality, cardiovascular events, and fractures. All of the study participants in Andersen et al. [79] had concomitant heart disease and some (i.e., participants living in Copenhagen with concomitant heart disease and having had a TSH test done between 2000 and 2009) may have also been included in the Andersen et al. [78] cohort study.^2^ The duration of follow-up for the trial by Stott et al. was up to 3 years while the duration of observation in the cohort studies was up to 8 years for Andersen et al. [78], 9 years for Razvi et al. [80], and up to 14 years for Andersen et al. [79]. The median dose of levothyroxine at 1 year follow-up in the RCT was 50 μg/day while the estimated mean (standard deviation) or median (interquartile range) dose received by the participants in the Andersen et al. [79], Andersen et al. [78] and Razvi et al. [80] cohort studies were as follows: 76.6 ± 32.1 μg/day, 79.7 ± 30.8 μg/day, and 75 μg/day (range 12.5–175.0), respectively. Stott et al. [73] only included participants ≥ 65 years of age while the cohort studies [78, 80] included adults of all ages and reported data separately for adults ≥ 65 years or > 70 years and adults < 65 or ≤ 70 years.

Outcomes were reported as new events following start of treatment, placebo, or period of observation. Incidence rate ratios (IRR) for Andersen et al. [78] and Andersen et al. [79] were adjusted for age, sex, and Charlson Comorbidity Index. Hazard ratios (HR) for Razvi et al. [80] were adjusted for multiple variables including age, sex, BMI, socioeconomic deprivation score, total cholesterol level, index TSH levels, and comorbidity, and HR for Stott et al. [73] were adjusted for country, sex, and starting dose of levothyroxine.

#### All-cause mortality

##### Adults (18 years and older)

One RCT [73] involving 737 adults (all ≥ 65 years of age) with subclinical hypothyroidism found no statistically significant difference in the number of deaths from all causes between those treated with levothyroxine (10 deaths) versus placebo (5 deaths; HR 1.91; 95% CI 0.65–5.60). The overall quality of this body of evidence was rated as low due to downgrading for indirectness (evidence was only in older adults) and imprecision.

##### Adults (< 65 or ≤ 70 years or of age)

Two retrospective cohort studies [78, 80] including adults between 40 and 70 or < 65 years of age with subclinical hypothyroidism reported statistically significant lower all-cause mortality in the levothyroxine-treated group compared to those not treated with levothyroxine. One cohort study [78] (*n* = 12,212)^3^ reported an adjusted IRR 0.63 (95% CI 0.40–0.99) and the other [80] (*n* = 3093)^3^ reported a multivariate adjusted HR 0.36 (95% CI 0.19–0.66) (absolute value of 41 fewer deaths per 1000, ranging from 52 fewer to 21 fewer). The overall quality for this body of evidence was rated as very low due to downgrading for study design and inconsistency.

##### Adults (> 65 years of age)

One RCT [73] involving 737 older adults (all ≥ 65 years of age) with subclinical hypothyroidism found no statistically significant difference in the number of deaths from all causes between those treated with levothyroxine (10 deaths) versus placebo (5 deaths; HR 1.91; 95% CI 0.65–5.60). The overall quality for this body of evidence was rated as moderate due to downgrading for imprecision.

##### Females

Two retrospective cohort studies [78, 79] found no statistically significant difference in the number of deaths from all causes between females treated and not treated for subclinical hypothyroidism. One cohort study [78] (*n* = 9743)^4^ reported an adjusted IRR 0.99 (95% CI 0.85–1.16) and the other [79] (*n* = 760)^4^ reported an adjusted IRR 1.08 (95% CI 0.80–1.48). The overall quality for this body of evidence was rated as very low due to downgrading for study design and imprecision.

##### Males

Two retrospective cohort studies [78, 79] found no statistically significant difference in the number of deaths from all causes between males treated and not treated for subclinical hypothyroidism. One cohort study [78] (*n* = 2469)^4^ reported an adjusted IRR 1.24 (95% CI 0.89–1.16) and the other [79] (*n* = 432)^4^ reported an adjusted IRR 1.43 (95% CI 0.87–2.34). The overall quality for this body of evidence was rated as very low due to downgrading for study design and imprecision.

#### Deaths due to cardiovascular diseases

##### Adults (18 years and older)

One RCT [73] involving 737 adults (all ≥ 65 years of age) with subclinical hypothyroidism found no statistically significant difference in the number of cardiovascular deaths between those treated with levothyroxine (2 deaths) versus placebo (1 death; OR 2.01; 95% CI 0.18–22.27). The overall quality for this body of evidence was rated as very low due to downgrading for indirectness (evidence was only in older adults) and very serious concerns with imprecision.

##### Adults (< 65 or ≤ 70 years of age)

One retrospective cohort study [80] (*n* = 3093)^3^ including adults between 40 and 70 years of age with subclinical hypothyroidism found a statistically significant difference in the number of deaths due to circulatory diseases between those treated (23 deaths) and not treated (38 deaths) with levothyroxine (multivariate adjusted HR 0.54; 95% CI 0.37–0.92) (absolute value of 12 fewer deaths per 1000, ranging from 16 fewer to 2 fewer). However, the other cohort study [78] (*n* = 12,212)^3^ including adults < 65 years of age did not find a statistically significant difference in cardiovascular deaths between those treated and not treated for subclinical hypothyroidism (adjusted IRR 0.55; 95% CI 0.25–1.20). The overall quality for this body of evidence was rated as very low due to downgrading for study design and imprecision.

##### Adults (> 65 years of age)

One RCT [73] involving 737 older adults (all ≥ 65 years of age) with subclinical hypothyroidism found no statistically significant difference in the number of cardiovascular deaths between those treated with levothyroxine versus placebo (OR 2.01; 95% CI 0.18–22.27). The overall quality for this body of evidence was rated as low due to downgrading for very serious concerns with imprecision.

##### Females

One retrospective cohort study [78] (*n* = 9743)^4^ found no statistically significant difference in the number of cardiovascular deaths between females treated and not treated for subclinical hypothyroidism (adjusted IRR 0.96; 95% CI 0.77–1.21). The overall quality for this body of evidence was rated as very low due to downgrading for study design and imprecision.

##### Males

One retrospective cohort study [78] (*n* = 2469)^4^ found no statistically significant difference in the number of cardiovascular deaths between males treated and not treated for subclinical hypothyroidism (adjusted IRR 1.32; 95% CI 0.83–2.08). The overall quality for this body of evidence was rated as very low due to downgrading for study design and imprecision.

#### Fatal and non-fatal cardiovascular events (not including atrial fibrillation)

##### Adults (18 years and older)

One RCT [73] involving 737 adults (all ≥ 65 years of age) with subclinical hypothyroidism found no statistically significant difference in the number of fatal and non-fatal cardiovascular events between those treated with levothyroxine (18 events) versus placebo (20 events; HR 0.89; 95% CI 0.47–1.69). The overall quality for this body of evidence was rated as low due to downgrading for indirectness (evidence was only in older adults) and imprecision.

##### Adults (< 65 or ≤ 70 years of age)

One retrospective cohort study [80] (*n* = 3093)^3^ including adults between 40 and 70 years of age with subclinical hypothyroidism found a statistically significant difference in the number of fatal and non-fatal ischemic heart disease events between those treated (68 events) and not treated (97 events) with levothyroxine (multivariate adjusted HR 0.61; 95% CI 0.39–0.95) (absolute value of 25 fewer events per 1000, ranging from 40 fewer to 3 fewer). However, the same cohort study [80] did not find a statistically significant difference in the number of fatal and non-fatal cerebrovascular disease events between those treated (55 events) and not treated (44 events) for subclinical hypothyroidism (multivariate adjusted HR 1.03; 95% CI 0.51–2.13). In addition, the other cohort study [78] (*n* = 12,212)^3^ including adults < 65 years of age did not find a statistically significant difference in the number of myocardial infarction events (adjusted IRR of 1.11; 95% CI 0.61–2.02). The overall quality for this body of evidence was rated as very low due to downgrading for study design and imprecision.

##### Adults (> 65 years of age)

One RCT [73] involving 737 older adults (≥ 65 years of age) with subclinical hypothyroidism found no statistically significant difference in the number of fatal and non-fatal cardiovascular events between those treated with levothyroxine (18 events) versus placebo (20 events; HR 0.89; 95% CI 0.47–1.69). The overall quality for this body of evidence was rated as moderate due to downgrading for imprecision.

##### Females

Two retrospective cohort studies [78, 79] found no statistically significant difference in the number of fatal and non-fatal cardiovascular events in females treated and not treated for subclinical hypothyroidism. One cohort study [78] (*n* = 9743)^4^ reported an adjusted IRR 0.99 (95% CI 0.70–1.38) for myocardial infarction events and the other [79] (*n* = 760)^4^ reported an adjusted IRR 0.99 (95% CI 0.70–1.40) for major adverse cardiac events. The overall quality for this body of evidence was rated as very low due to downgrading for study design and imprecision.

##### Males

Two retrospective cohort studies [78, 79] found no statistically significant difference in the number of fatal and non-fatal cardiovascular events in males treated and not treated for subclinical hypothyroidism. One cohort study [78] (*n* = 2469)^4^ reported an adjusted IRR 1.41 (95% CI 0.83–2.40) for myocardial infarction events and the other [79] (*n* = 432)^4^ reported an adjusted IRR 1.36 (95% CI 0.79–2.35) for major adverse cardiac events. The overall quality for this body of evidence was rated as very low due to downgrading for study design and imprecision.

#### Atrial fibrillation

##### Adults (18 years and older)

One RCT [73] involving 737 adults (all ≥ 65 years of age) with subclinical hypothyroidism found no statistically significant difference in the number of new-onset atrial fibrillation events between those treated with levothyroxine (11 events) or placebo (13 events; HR 0.80; 95% CI 0.35–1.80). The overall quality for this body of evidence was rated as low due to downgrading for indirectness (evidence was only in older adults) and imprecision.

##### Adults (< 65 or ≤ 70 years of age)

One retrospective cohort study [80] (*n* = 3093)^3^ including adults between 40 and 70 years of age with subclinical hypothyroidism did not find a statistically significant difference in the number of atrial fibrillation events between those treated (35 events) and not treated (36 events) with levothyroxine (multivariate adjusted HR 0.76 (95% CI 0.26–1.73). The overall quality for this body of evidence was rated as very low due to downgrading for imprecision.

##### Adults (> 65 years of age)

One RCT [73] involving 737 older adults (≥ 65 years of age) with subclinical hypothyroidism found no statistically significant difference in the number of new-onset atrial fibrillation events between those treated with levothyroxine (11 events) or placebo (13 events; HR 0.80; 95% CI 0.35–1.80). The overall quality for this body of evidence was rated as moderate due to downgrading for imprecision.

##### Fractures

One RCT [73] involving 737 adults (all ≥ 65 years of age) with subclinical hypothyroidism found no statistically significant difference in the number of fractures between those treated with levothyroxine (9 fractures) or placebo (8 fractures; HR 1.06; 95% CI 0.41–2.76). The overall quality for this body of evidence was rated as low due to downgrading for indirectness (evidence was only in older adults) and imprecision.

### Quality of life

Five RCTs were included [63, 70–73]: one [73] with an assessment of low risk of bias across all domains, two [63, 71] with low risk of bias for blinding of participants and personnel and blinding of outcome assessment, and two [70, 72] with uncertain or high risk of bias for sequence generation and blinding reported on the effects of treatment versus no treatment for subclinical hypothyroidism on QoL. RCTs providing results using different measures of QoL within similar constructs were grouped into the following categories to help with the synthesis and interpretation of the results: thyroid-related QoL, fatigue/tiredness, mental well-being, physical well-being, and general QoL.

The duration of follow-up was fairly short for RCTs by Najafi et al. [70] and Reuters et al. [72] (12 weeks to 6 months) and longer for the remainder (12 months up to 3 years) [63, 71, 73]. The mean/median dose of levothyroxine administered to the treatment group close to final follow-up was 50 μg/day in two RCTs [71, 73], between 100.0 and 109.7 μg/day in two other RCTs [63, 70], and not reported in the fifth RCT [72]. Three RCTs [63, 71, 73] included participants with mean ages ≥ 60 years while the other two [70, 72] enrolled participants with mean ages of 35 or 51 years. Mean TSH levels of participants at baseline ranged from 5.3 to 8.3 mIU/L. A variety of QoL measures were used and brief descriptions of the measures are provided in Additional file 1.

### Quality of life (QoL)

#### Thyroid-related QoL (not including tiredness)

##### At 12 months

Thyroid-related QoL was assessed using the ThyPRO Hypothyroid Symptoms scale in one RCT [73] involving 638 adults (all ≥ 65 years of age) with subclinical hypothyroidism. The ThyPRO Hypothyroid Symptoms scale consists of 4 items, and scores range from 0 to 100; a difference of 9 is considered a meaningful difference (see Additional file 1). The RCT included in this review found no statistically significant difference in the ThyPRO hypothyroid symptoms score at 12 months between those treated with levothyroxine or placebo (MD 0.0; 95% CI − 2.0 to 2.1). The overall quality for this body of evidence was rated as moderate due to downgrading for indirectness (evidence was only in older adults).

##### At extended follow-up (over 12 months up to 3 years)

One RCT [73] involving between 381 and 648 adults (depending on the outcome measure and time of follow-up), all ≥ 65 years of age with subclinical hypothyroidism, found no statistically significant difference in thyroid-related QoL measures at extended follow-up between those treated with levothyroxine or placebo. The RCT reported a MD 1.0 (95% CI − 1.9 to 3.9) in the ThyPRO Hypothyroid Symptoms score and a MD − 0.5 (95% CI − 2.2 to 1.3) in the Comprehensive ThyPRO-39 score. The overall quality for this body of evidence was rated as moderate due to downgrading for indirectness (evidence was in older adults).

#### Fatigue/tiredness

##### At 12 months

Fatigue/tiredness was assessed using the ThyPRO Hypothyroid Tiredness scale in one RCT [73] involving 638 adults (all ≥ 65 years of age) with subclinical hypothyroidism. The ThyPRO Hypothyroid Tiredness scale consists of 7 items, and scores range from 0 to 100; a difference of 9 is considered a clinically meaningful difference (see Additional file 1). The RCT included in this review found no statistically significant difference in the ThyPRO Hypothyroid Tiredness score at 12 months between those treated with levothyroxine or placebo (MD 0.4; 95% CI − 2.1 to 2.9). The overall quality for this body of evidence was rated as moderate due to downgrading for indirectness (evidence was only in older adults).

##### At extended follow-up (over 12 months up to 3 years)

One RCT [73] involving 381 adults (all ≥ 65 years of age) with subclinical hypothyroidism found no statistically significant difference in the ThyPRO Hypothyroid Tiredness score at extended follow-up between those treated with levothyroxine or placebo (MD − 3.5; 95% CI − 7.0 to 0.0). The overall quality for this body of evidence was rated as moderate due to downgrading for indirectness (evidence was only in older adults).

##### Mental well-being

Four RCTs [63, 70–72] did not find statistically significant differences between those treated and not treated for subclinical hypothyroidism on measures of mental well-being. The measures included the Beck Depression Inventory, the Hamilton Scale for Anxiety and Depression, and the Hospital Anxiety and Depression Scale (see Additional file 1 for details about these scales). The findings are summarized in Table 1. The overall quality for this body of evidence was rated as moderate due to downgrading for imprecision.

**Table 1:** Summary of differences between groups on measures of mental well-being

Scale	Author (total sample size; treatment vs. control)	Difference*	95% CI
Beck Depression Inventory	Jorde et al. [63] (69; 36 vs. 33)	1.00*	− 0.80 to 2.80
	Najafi et al. [70] (60; 30 vs. 30)	0.51*	− 4.74 to 5.76
	Reuters et al. [72] (57; 25 vs. 32)	− 0.30**	− 3.12 to 2.52
Hamilton Scale for Anxiety	Reuters et al. [72] (57; 25 vs. 32)	0.50**	− 2.81 to 3.81
Hamilton Scale for Depression	Reuters et al. [72] (57; 25 vs. 32)	− 1.00**	− 2.49 to 0.49
Hospital Anxiety and Depression Scale	Parle et al. [71] (85; 49 vs. 36)	0.30*	− 0.86 to 1.46

*Value is the difference in mean scores at final follow-up between treatment and control groups

**Value is the difference in mean variation scores from baseline to follow-up between treatment and control group

### Physical well-being

One RCT [73] involving between 646 and 647 adults (depending on the outcome measure), all ≥ 65 years of age with subclinical hypothyroidism, found no statistically significant difference in measures of physical well-being between those treated with levothyroxine or placebo. The RCT reported a MD − 0.1 (95% CI − 0.3 to 1.0) in the Barthel Index, basic activities of daily living scores, and a MD − 0.1 (95% CI − 0.3 to 1.0) in the Older American Resources and Services, instrumental activities of daily living scores (see Additional file 1 for details about these scales). The overall quality for this body of evidence was rated as moderate due to downgrading for indirectness (evidence was only in older adults).

### General well-being

Three RCTs [63, 72, 73] did not find statistically significant differences between those treated and not treated for subclinical hypothyroidism on measures of general well-being. The measures included the EUROQUOL Group 5-Dimension report questionnaire descriptive and visual analog scales, the General Health Questionnaire, and the Medical Outcomes Study 36-item Short Form Health Survey (see Additional file 1 for details about these scales). The findings are summarized in Table 2. The overall quality for this body of evidence was rated as moderate due to downgrading for imprecision.

**Table 2:** Summary of differences between groups on measures of general well-being

Scale	Author (total sample size; treatment vs. control)	Difference*	95% CI
EUROQUOL Group 5-Dimension Report Questionnaire Descriptive Score	Stott et al. [73] (638; 318 vs. 320)	− 0.03*	− 0.05 to 0.00 (*p* = 0.05)
EUROQUOL Group 5-Dimension Report Questionnaire Visual Analogue Scale Score	Stott et al. [73] (638; 318 vs. 320)	− 1.3*	− 3.2 to 0.6
General Health Questionnaire	Jorde et al. [63] (69; 36 vs. 33)	0.70*	− 0.58 to 1.98
Medical Outcomes Study 36-item Short Form Health Survey	Reuters et al. [72] (57; 25 vs. 32)	0.30**	− 0.43 to 1.03

*Value is the difference in mean scores at final follow-up between treatment and control groups

**Value is the difference in mean variation scores from baseline to follow-up between treatment and control group

### Cognitive function

Three RCTs [63, 71, 73], one [73] with low risk of bias across all domains assessed and two [63, 71] with low risk of bias for blinding of participants and personnel and blinding of outcome assessment reported on the effects of treatment versus no treatment for subclinical hypothyroidism on cognitive function. The duration of follow-up was from 12 months to 3 years. The mean/median dose of levothyroxine administered to the treatment group close to final follow-up was 50 μg/day in two RCTs [71, 73] and 109.7 μg/day in one RCT [63]. All three RCTs included participants with mean age ≥ 60 years. Mean TSH levels of participants at baseline ranged from 5.3 to 6.6 mIU/L. A variety of cognitive function measures were used and brief descriptions of the measures are provided in Additional file 1.

### Cognitive function

Three RCTs [63, 71, 73] found no statistically significant differences between those treated and not treated for subclinical hypothyroidism on eighteen different measures of cognitive function. Unadjusted calculations performed for this systematic review found a statistically significant improvement in the treatment groups for the Speed and Capacity of Language Processing (SCOLP) test (MD 1.47, 95% CI 0.05–2.89, *p* = 0.04) [71] and the composite cognitive score (MD 2.4, 95% CI 0.29–4.51, *p* = 0.03) [63]. The findings for the various cognitive function tests are summarized in Table 3. See Additional file 1 for details about these scales. The overall quality for this body of evidence was rated as low due to downgrading for inconsistency and imprecision.

**Table 3:** Summary of differences between groups at final follow-up on measures of cognitive function

Test	Author (total sample size; treatment vs. control)	Difference*	95% CI
California Computerized Assessment Package	Jorde et al. [63] (68; 35 vs. 33)	− 79.00	− 229.88 to 71.88
Composite cognitive score	Jorde et al. [63] (65; 35 vs. 30)	2.40	0.29–4.51
Controlled Word Association test	Jorde et al. [63] (69; 36 vs. 33)	0.10	− 6.77 to 6.97
Letter Digit Coding test	Stott et al. [73] (600; 302 vs. 298)	− 0.1	− 0.9 to 0.7
Middlesex Elderly Assessment of Mental State	Parle et al. [71] (82; 46 vs. 36)	0.34	− 0.08 to 0.76
Mini-Mental State Examination	Parle et al. [71] (82; 46 vs. 36)	0.03	− 0.89 to 0.95
Seashore Rhythm test	Jorde et al. [63] (68; 35 vs. 33)	− 37.00	− 89.85 to 15.85
Speed and Capacity of Language Processing test	Parle et al. [71] (85; 49 vs. 36)	1.47	0.05–2.89
Trail Making Test A	Jorde et al. [63] (69; 36 vs. 33)	− 5.10	− 12.84 to 2.64
	Parle et al. [71] (84; 48 vs. 36)	− 2.45	− 11.1 to 6.2
Trail Making Test B	Jorde et al. [63] (66; 36 vs. 30)	− 9.00	− 35.79 to 17.79
	Parle et al. [71] (82; 48 vs. 34)	− 11.71	− 45.20 to 21.78
Trail Making Test B-A	Parle et al. [71] (82; 48 vs. 34)	−11.61	−37.91 to 14.69
Vocabulary – Wechsler Intelligence Scale	Jorde et al. [63] (69; 36 vs. 33)	0.10	− 1.73 to 1.93
Word List test	Jorde et al. [63] (68; 35 vs. 33)	1.10	− 2.63 to 4.83

*Value is the difference in mean scores at final follow-up between treatment and control groups

***KQ3b: Does treatment of screen-detected overt or subclinical TD improve intermediate outcomes?***

The summary of the findings are described below. Further details on the evidence, including summary of findings tables and GRADE evidence profile tables for outcomes for KQ3b can be found in Additional file 2: Evidence Set 2. Characteristics of the individual RCTs, individual study results, and risk of bias assessments can be found in Additional file 1.

### Intermediate outcomes

Fifteen RCTs [57–60, 62, 64–69, 73–77], 13 with low risk of bias for blinding of participants and personnel, 10 with low risk of bias for blinding of outcome assessment, and the majority with unclear risk of bias for the other risk of bias domains that were assessed, reported on the effects of treatment versus no treatment for subclinical hypothyroidism on intermediate outcomes. The duration of follow-up was < 6 months in five RCTs [58, 59, 65, 68, 69] and 10 RCTs [57, 60, 62, 64, 66, 67, 73, 74, 76, 77] had follow-up durations from 8 months to 3 years. Eleven RCTs [58, 59, 62, 64, 66–69, 73, 74, 76] studied the effects of treatment with levothyroxine versus placebo, while the other four [57, 60, 65, 77] compared treatment with levothyroxine to no treatment (observation). All but three RCTs [62, 69, 73] had participants with mean ages of < 60 years. The mean/median dose of levothyroxine administered to the treatment group close to final follow-up was ≤ 50 μg/day in six RCTs [57, 64, 65, 69, 73, 77] (ranging from 24 to 50 μg/day), was > 50 μg/day in eight RCTs [58–60, 62, 66–68, 76] (ranging from 64 to 100 μg/day), and the information was not provided in one RCT [74, 75], although exceeding a dosage of 75 μg/day was a reason for exclusion from the trial. Mean TSH levels of participants at baseline ranged from 4.65 to 11.0 mIU/L. As much as possible, outcomes were reported as a difference in mean results at follow-up between the treatment and control group.

#### Bone mineral density

No studies reporting on the effects of treating asymptomatic, non-pregnant adults for TD on bone mineral density were found.

#### Cholesterol/lipid levels

##### Total cholesterol

Ten RCTs [57, 58, 60, 62, 64, 66, 68, 69, 74, 77] reported the effects on total cholesterol (TC) of treatment compared to no treatment/placebo for subclinical hypothyroidism in asymptomatic non-pregnant adults. Results were mixed: six RCTs [57, 58, 60, 62, 69, 74] did not find a statistically significant difference between the two groups (*p* > 0.05), three RCTs [64, 66, 68] found that mean values for TC levels at final follow-up were less in the treatment group compared to the control group (*p* < 0.05), and one RCT [77] found the decline in mean TC levels from baseline to follow-up was statistically significantly larger in the treatment group than in the control group (*p* < 0.05) (Tables 4 and 5). Difference in mean TC levels between treatment and control groups at final follow-up ranged from − 1.07 to 0.00 mmol/L. The individual RCT findings are summarized below. The overall quality for this body of evidence was rated as moderate due to some concerns around risk of bias and inconsistency.

**Table 4:** Difference in means between treatment and control groups for TC at final follow-up

Author	Sample size (treatment group vs. control group)	Difference	95% CI
Cabral et al. [57]	32 (14 vs. 18)	− 1.07 mmol/L*	− 2.49 to 0.36
Caraccio et al. [58]	49 (24 vs. 25)	− 0.30 mmol/L*	− 0.92 to 0.32
Duman et al. [60]	39 (20 vs. 19)	0.00 mmol/L*	− 0.98 to 0.98
Iqbal et al. [62]	64 (32 vs. 32)	− 0.10 mmol/L*	− 0.59 to 0.39
Liu et al. [64]	119 (60 vs. 59)	− 0.29 mmol/L**	− 0.54 to − 0.04
Mikhail et al. [66]	120 (60 vs. 60)	− 0.30 mmol/L*	− 0.58 to − 0.30
Monzani et al. [68]	45 (23 vs. 22)	− 1.56 mmol/L*	− 2.91 to − 0.20
Nagasaki et al. [69]	95 (48 vs. 47)	− 0.14 mmol/L**	− 0.54 to 0.26
Teixeira et al. [74, 75]	26 (11 vs. 15)	− 0.32 mmol/L*	− 1.79 to 1.16

*Value is the difference in mean scores at final follow-up between treatment and control groups

**Value is the difference in mean variation scores from baseline to follow-up between treatment and control group

**Table 5:** Comparison of change from baseline values to final follow-up between treatment and control groups for TC

Author	Sample size (treatment group vs. control group)	Results*	*p* value
Zhao et al. [77]	369 (210 vs. 159)	The decline in the treatment group (− 0.41 mmol/L) was statistically significantly larger than the decline in the control group (− 0.17 mmol/L)	*p* = 0.012

*Mean difference could not be calculated with the data available

### Low-density lipoprotein

Ten RCTs [57, 58, 60, 62, 64, 66, 68, 69, 74, 77] reported the effects on low-density lipoprotein (LDL) of treatment compared to no treatment/placebo for subclinical hypothyroidism in asymptomatic non-pregnant adults. Eight [57, 58, 60, 62, 64, 66, 69, 74] RCTs did not find a statistically significant difference in LDL levels between those treated and not treated for subclinical hypothyroidism; one RCT did not report the difference between groups (Tables 6 and 7). Difference in means between treatment and control groups at final follow-up ranged from − 1.23 to 0.11 mmol/L. The individual RCT findings are summarized below. The overall quality for this body of evidence was rated as moderate due to some concerns around risk of bias and inconsistency.

**Table 6:** Difference in means between treatment and control groups for LDL at final follow-up

Author	Sample size (treatment group vs. control group)	Difference	95% CI
Cabral et al. [57]	32 (14 vs. 18)	− 0.99 mmol/L*	− 2.40 to 0.42
Caraccio et al. [58]	49 (24 vs. 25)	− 0.30 mmol/L*	− 0.83 to 0.23
Duman et al. [60]	39 (20 vs. 19)	0.11 mmol/L*	− 0.89 to 1.11
Iqbal et al. [62]	64 (32 vs. 32)	0.00 mmol/L*	− 0.47 to 0.47
Liu et al. [64]	119 (60 vs. 59)	− 0.12 mmol/L**	− 0.32 to 0.08
Mikhail et al. [66]	120 (60 vs. 60)	− 0.21 mmol/L*	− 0.46 to 0.03
Monzani et al. [68]	45 (23 vs. 22)	− 1.23 mmol/L*	− 2.32 to − 0.13
Nagasaki et al. [69]	95 (48 vs. 47)	− 0.22 mmol/L**	− 0.70 to 0.26
Teixeira et al. [74, 75]	26 (11 vs. 15)	− 0.63 mmol/L*	− 1.90 to 0.64

*Value is the difference in mean scores at final follow-up between treatment and control groups

**Value is the difference in mean variation scores from baseline to follow-up between treatment and control group

**Table 7:** Comparison of change from baseline values to final follow-up between treatment and control groups for LDL

Author	Sample size (treatment group vs. control group)	Results*	*p* value
Zhao et al. [77]	369 (210 vs. 159)	LDL levels declined by 0.09 mmol/L in the treatment group and declined by 0.10 mmol/L in the control group.	Not reported

*Mean difference could not be calculated with the data available

### High-density lipoprotein

Ten RCTs [57, 58, 60, 62, 64, 66, 68, 69, 74, 77] reported the effects on high-density lipoprotein (HDL) of treatment compared to no treatment/placebo for subclinical hypothyroidism in asymptomatic non-pregnant adults. None of the RCTs found a statistically significant difference in HDL levels between those treated and not treated for subclinical hypothyroidism; one RCT did not report the difference between groups. Difference in means between treatment and control groups at final follow-up ranged from − 0.17 to 0.26 mmol/L. The individual RCT findings are summarized in Tables 8 and 9. The overall quality for this body of evidence was rated as moderate due to some concerns around risk of bias and inconsistency.

**Table 8:** Difference in means between treatment and control groups for HDL at final follow-up

Author	Sample size (treatment group vs. control group)	Difference	95% CI
Cabral et al. [57]	32 (14 vs. 18)	0.26 mmol/L*	− 0.17 to 0.70
Caraccio et al. [58]	49 (24 vs. 25)	− 0.10 mmol/L*	− 0.27 to 0.07
Duman et al. [60]	39 (20 vs. 19)	0.004 mmol/L*	− 0.45 to 0.44
Iqbal et al. [62]	64 (32 vs. 32)	0.00 mmol/L*	− 0.22 to 0.22
Liu et al. [64]	119 (60 vs. 59)	0.03 mmol/L**	− 0.03 to 0.09
Mikhail et al. [66]	120 (60 vs. 60)	0.09 mmol/L*	− 0.01 to 0.20
Monzani et al. [68]	45 (23 vs. 22)	− 0.17 mmol/L*	− 0.49 to 0.14
Nagasaki et al. [69]	95 (48 vs. 47)	0.02 mmol/L**	− 0.12 to 0.16
Teixeira et al. [74, 75]	26 (11 vs. 15)	0.35 mmol/L*	− 0.29 to 0.99

*Value is the difference in mean scores at final follow-up between treatment and control groups

**Value is the difference in mean variation scores from baseline to follow-up between treatment and control group

**Table 9:** Comparison of change from baseline values to final follow-up between treatment and control groups for HDL

Author	Sample size (treatment group vs. control group)	Results*	*p* value
Zhao et al. [77]	369 (210 vs. 159)	HDL levels declined by 0.05 mmol/L in the treatment group and increased by 0.07 mmol/L in the control group.	Not reported

*Mean difference could not be calculated with the data available

### Triglycerides

Ten RCTs [57, 58, 60, 62, 64, 66, 68, 69, 74, 77] reported the effects on triglycerides (TG) of treatment compared to no treatment/placebo for subclinical hypothyroidism in asymptomatic non-pregnant adults. Nine [57, 58, 62, 64, 66, 68, 69, 74, 77] out of the 10 RCTs did not find a statistically significant difference in TG levels between those treated and not treated for subclinical hypothyroidism. Difference in means between treatment and control groups at final follow-up ranged from − 1.72 to 0.12 mmol/L. The individual RCT findings are summarized in Tables 10 and 11. The overall quality for this body of evidence was rated as moderate due to some concerns around risk of bias and inconsistency.

**Table 10:** Difference in means between treatment and control groups for TG at final follow-up

Author	Sample size (treatment group vs. control group)	Difference	95% CI
Cabral et al. [57]	32 (14 vs. 18)	− 1.72 mmol/L*	− 3.51 to 0.07
Caraccio et al. [58]	49 (24 vs. 25)	− 0.10 mmol/L*	− 0.46 to 0.26
Duman et al. [60]	39 (20 vs. 19)	− 1.94 mmol/L*	− 3.65 to − 0.24
Iqbal et al. [62]	64 (32 vs. 32)	− 0.10 mmol/L*	− 0.52 to 0.32
Liu et al. [64]	119 (60 vs. 59)	− 0.08 mmol/L**	− 0.26 to 0.10
Mikhail et al. [66]	120 (60 vs. 60)	− 0.11 mmol/L*	− 0.31 to 0.09
Monzani et al. [68]	45 (23 vs. 22)	− 0.81 mmol/L*	− 2.22 to 0.60
Nagasaki et al. [69]	95 (48 vs. 47)	0.12 mmol/L**	− 0.17 to 0.41
Teixeira et al. [74, 75]	26 (11 vs. 15)	− 0.98 mmol/L*	− 3.52 to 1.55

*Value is the difference in mean scores at final follow-up between treatment and control groups

**Value is the difference in mean variation scores from baseline to follow-up between treatment and control group

**Table 11:** Comparison of change from baseline values to final follow-up between treatment and control groups for TG

Author	Sample size (treatment group vs. control group)	Results*	*p* value
Zhao et al. [77]	369 (210 vs. 159)	The decline in the control group (− 0.11 mmol/L) was similar to the decline in the treatment group (− 0.17 mmol/L)	*p* ≥ 0.05

*Mean difference could not be calculated with the data available

#### Blood pressure

##### Systolic blood pressure

Eight RCTs [64, 65, 67–69, 73, 76, 77] reported the effects on systolic blood pressure (SBP) of treatment compared to no treatment/placebo for subclinical hypothyroidism in asymptomatic non-pregnant adults. None of the RCTs found a statistically significant difference in SBP readings at final follow-up between those treated and not treated for subclinical hypothyroidism. Difference in means between treatment and control groups at final follow-up ranged from − 12.25 to 0.50 mmHg. The individual RCT findings are summarized in Table 12. The overall quality for this body of evidence was rated as moderate due to some concerns around imprecision and inconsistency.

**Table 12:** Difference in means between treatment and control groups for SBP at final follow-up

Author	Sample size (treatment group vs. control group)	Difference	95% CI
Liu et al. [64]	119 (60 vs. 59)	− 1.00 mmHg**	− 3.87 to 1.87
Mainenti et al. [65]	23 (11 vs. 12)	− 12.25 mmHg*	− 29.53 to 5.03
Monzani et al. [67]	20 (10 vs. 10)	0.50 mmHg*	− 6.79 to 7.79
Monzani et al. [68]	45 (23 vs. 22)	− 2.00 mmHg*	− 10.19 to 6.19
Nagasaki et al. [69]	95 (48 vs. 47)	− 3.40 mmHg**	− 10.56 to 3.76
Stott et al. [73]	638 (318 vs. 320)	− 0.1 mmHg*	− 2.1 to 2.4
Yazici et al. [76]	45 (23 vs. 22)	0.50 mmHg*	− 5.23 to 6.23
Zhao et al. [77]	369 (210 vs. 159)	− 2.54 mmHg*	− 6.65 to 1.57

*Value is the difference in mean scores at final follow-up between treatment and control groups

**Value is the difference in mean variation scores from baseline to follow-up between treatment and control group

### Diastolic blood pressure

Eight RCTs [64, 65, 67–69, 73, 76, 77] reported the effects on diastolic blood pressure (DBP) of treatment compared to no treatment/placebo for subclinical hypothyroidism in asymptomatic non-pregnant adults. None of the RCTs found a statistically significant difference in DBP readings at final follow-up between those treated and not treated for subclinical hypothyroidism. Difference in means between treatment and control groups at follow-up ranged from − 5.4 to 3.8 mmHg. The individual RCT findings are summarized in Table 13. The overall quality for this body of evidence was rated as moderate due to some concerns around imprecision and inconsistency.

**Table 13:** Difference in means between treatment and control groups for DBP at final follow-up

Author	Sample size (treatment group vs. control group)	Difference	95% CI
Liu et al. [64]	119 (60 vs. 59)	− 1.00 mmHg**	− 3.34 to 1.34
Mainenti et al. [65]	23 (11 vs. 12)	− 5.4 mmHg*	− 11.08 to 0.28
Monzani et al. [67]	20 (10 vs. 10)	3.80 mmHg*	− 2.42 to 10.02
Monzani et al. [68]	45 (23 vs. 22)	− 3.00 mmHg*	− 7.97 to 1.97
Nagasaki et al. [69]	95 (48 vs. 47)	− 0.10 mmHg**	− 4.22 to 4.02
Stott et al. [73]	638 (318 vs. 320)	− 0.1 mmHg*	− 1.5 to 1.3
Yazici et al. [76]	45 (23 vs. 22)	− 0.50 mmHg*	− 5.53 to 4.53
Zhao et al. [77]	369 (210 vs. 159)	− 0.13 mmHg*	− 2.42 to 2.16

*Value is the difference in mean scores at final follow-up between treatment and control groups

**Value is the difference in mean variation scores from baseline to follow-up between treatment and control group

#### Weight change/body mass index

Twelve RCTs [58–60, 62, 64, 67–69, 73, 74, 76, 77] reported the effects on BMI of treatment compared to no treatment/placebo for subclinical hypothyroidism in asymptomatic non-pregnant adults. Ten [58–60, 62, 64, 67–69, 73, 76] RCTs did not find a statistically significant difference in BMI readings at final follow-up between those treated and not treated for subclinical hypothyroidism; one RCT did not report on between group differences [77]. One small RCT [74] found a statistically significant difference in BMI readings at final follow-up, with those in the treatment group having a higher mean BMI than those in the control group at final follow-up. Difference in means between treatment and control groups at follow-up ranged from − 1.20 to 2.90 kg/m^2^. The individual RCT findings are summarized in Tables 14 and 15. The overall quality for this body of evidence was rated as moderate due to downgrading for imprecision.

**Table 14:** Difference in means between treatment and control groups for BMI at final follow-up

Author	Sample size (treatment group vs. control group)	Difference	95% CI
Caraccio et al. [58]	49 (24 vs. 25)	1.30 kg/m^2^*	− 0.33 to 2.93
Caraccio et al. [59]	23 (12 vs. 11)	− 0.40 kg/m^2^*	− 2.52 to 1.72
Duman et al. [60]	39 (20 vs. 19)	− 0.70 kg/m^2^*	− 2.91 to 1.51
Iqbal et al. [62]	64 (32 vs. 32)	1.40 kg/m^2^*	− 1.06 to 3.86
Liu et al. [64]	119 (60 vs. 59)	− 0.10 kg/m^2^**	− 0.51 to 0.31
Monzani et al. [67]	20 (10 vs. 10)	0.00 kg/m^2^*	− 3.22 to 3.22
Monzani et al. [68]	45 (23 vs. 22)	− 1.20 kg/m^2^*	− 3.34 to 0.94
Nagasaki et al. [69]	95 (48 vs. 47)	− 0.30 kg/m^2^**	− 1.26 to 0.66
Stott et al. [73]	638 (318 vs. 320)	0.0 kg/m^2^*	− 0.02 to 0.02
Teixeira et al. [74, 75]	26 (11 vs. 15)	2.90 kg/m^2^*	0.38–5.42
Yazici et al. [76]	45 (23 vs. 22)	− 0.20 kg/m^2^*	− 2.10 to 1.70

*Value is the difference in mean scores at final follow-up between treatment and control groups

**Value is the difference in mean variation scores from baseline to follow-up between treatment and control group

**Table 15:** Comparison of change from baseline values to final follow-up between treatment and control groups for BMI

Author	Sample size (treatment group vs. control group)	Results*	*p* value
Zhao et al. [77]	369 (210 vs. 159)	BMI declined by 0.2 kg/m^2^ in the treatment group and declined by 0.03 kg/m^2^ in the control group.	Not reported

*Mean difference could not be calculated with the data available

***KQ4: What are the harms of treating screen-detected TD in asymptomatic, non-pregnant adults?***

The summary of the findings is described below. Further details on the evidence, including summary of findings tables and GRADE evidence profile tables for outcomes for KQ4 can be found in Additional file 2: Evidence Set 3. Characteristics of the individual RCTs, individual study results, and risk of bias assessments can be found in Additional file 1.

### Harms due to treatment

Seven RCTs [61, 64, 69, 71, 73, 74, 77], one [73] with low risk of bias across all domains, four [64, 69, 71, 74] with low risk of bias for blinding of participants and personnel and/or low risk of bias for blinding of outcome assessment, and two [61, 77] with high or uncertain risk of bias for blinding and/or incomplete outcome data reported on the harms of treatment for subclinical hypothyroidism. The duration of follow-up was fairly short (6 months or less) in two RCTs [61, 69] and five RCTs [64, 71, 73, 74, 77] had follow-up durations from 48 weeks to 3 years. Five RCTs [64, 69, 71, 73, 74] studied the effects of treatment with levothyroxine versus placebo while the other two [61, 77] compared treatment with levothyroxine to no treatment (observation). More than half of the RCTs [61, 69, 71, 73] had participants with mean ages of ≥ 60 years. The mean/median dose of levothyroxine administered to the treatment group close to final follow-up was ≤ 50 μg/day in six RCTs [61, 64, 69, 71, 73, 77] (ranging from 25 to 50 μg/day) and not reported in one RCT [74], although exceeding a dosage of 75 μg/day was a reason for exclusion from the trial. Mean/median TSH levels of participants at baseline ranged from 5.5 to 8.2 mIU/L.

The RCTs did not provide a specific definition for harms or “adverse outcomes” such as adverse events (AEs), adverse reactions, adverse symptoms, adverse effects, or side effects; therefore, it is not known whether the trials used standard definitions and terminology.^5^ Rather, the terminology used and descriptive reports of the various adverse outcomes described in each RCT are provided in the tables below. Upon further inquiry, the definition of serious AEs used by one RCT was provided [73]: a serious AE or severe adverse reaction is “any AE or adverse reaction that results in death, is life threatening, requires hospitalization or prolongation of existing hospitalization, results in persistent or significant disability or incapacity, or consists of a congenital anomaly or birth defect” [82].

### Number of individuals reporting adverse outcomes

Five RCTs [61, 64, 69, 73, 77] reported on the number of individuals reporting adverse outcomes. Two RCTs [73, 77] did not find a statistically significant difference in the odds of reporting adverse outcomes at final follow-up between those treated and not treated for subclinical hypothyroidism but one RCT [64] found the odds of reporting adverse outcomes was statistically significantly higher in the treatment group than in the control group (OR 21.87; 95% CI 1.25–383.87). The ORs could not be calculated for the other RCTs [61, 69] due to missing data or because no adverse outcomes were reported in both the treatment and control groups. The proportion of adverse outcomes ranged from 0 to 8.2% in the control groups and 0 to 26.3% in the treatment groups. Individual RCT findings are summarized in Table 16. The overall quality for this body of evidence was rated as low due to downgrading for inconsistency and imprecision.

**Table 16:** Descriptive reports of adverse outcomes

Author (sample size; treatment vs. control)	Results	Descriptive reports of adverse outcomes (results continued)
Fadeyev et al. [61] (33; 19 vs. 14)	Treatment group: 5 events; 26.3%	In the treatment group, 5/19 patients had various AEs during treatment (3 patients had more ventricular premature beats and 2 patients had an increased mean heart rate in conjunction with an increased number of ventricular premature beats). At the end of the follow-up period, one of the patients in the treatment group had an unstable episode of ventricular tachycardia. AEs in the control group were not reported.
Liu et al. [64] (136; 68 vs. 68)	Control group (0 events; 0.0%) vs. treatment group (9 events; 13.2%)	OR 21.87 (95% CI 1.25–383.87); *p* = 0.03 Absolute value (range), 0 fewer per 1000 (from 0 fewer to 0 fewer) Adverse reactions included mild insomnia, mild diarrhea, mild paroxysmal supraventricular tachycardia, and palpitations.
Nagasaki et al. [69] (95; 48 vs. 47)	Control group (0 events; 0.0%) vs. treatment group (0 events; 0.0%)	None of the patients experienced side effects such as arrhythmia, angina pectoris, or hypertension that would have required withdrawal or reduction of the dose of levothyroxine.
Stott et al. [73] (737; 368 vs. 369)	Control group (103 events, 27.9%) vs. treatment group (78 events, 21.2%)	HR of 0.94 (95% CI of 0.88–1.0); *p* = 0.053 Absolute value (range), 14 fewer per 1000 (from 0 fewer to 29 fewer) For serious AEs only
Zhao et al. [77] (369; 210 vs. 159)	Control group (13 events; 8.2%) vs. treatment group (16 events; 7.6%)	OR 0.93 (95% CI 0.43–1.99); *p* ≥ 0.05 Absolute value (range), 5 fewer per 1000 (from 45 fewer to 69 more) Adverse symptoms included palpitations, chest tightness, dizziness, perspiration, low back pain, and hunched back. No participant attempted to visit a physician due to adverse effects.

### Withdrawal due to adverse outcomes

Four RCTs [64, 71, 74, 77] reported on the number of individuals withdrawing from the trial due to adverse outcomes. Three RCTs [64, 71, 74] did not find a statistically significant difference in the odds of withdrawing from the trial due to an adverse outcome between those treated and not treated for subclinical hypothyroidism. The ORs could not be calculated for the other RCT [77] because no events were reported in both the treatment and control groups. The proportion of withdrawals due to adverse outcomes ranged from 0 to 14.3% in the control groups and 0 to 9.6% in the treatment groups. The individual RCT findings are summarized in Table 17. The overall quality for this body of evidence was rated as low due to downgrading for inconsistency and imprecision.

**Table 17:** Descriptive reports of adverse outcomes leading to withdrawal from the trial

Author (Sample size (treatment vs. control)	Results	Descriptive reports of adverse outcomes (results continued)
Liu et al. [64] (136; 68 vs. 68)	Control group (0; 0.0% vs. treatment group (1; 1.5%)	OR: 3.04 (95% CI 0.12–76.06); *p* ≥ 0.05 Absolute value (range): 0 fewer per 1000 (from 0 fewer to 0 fewer) Adverse reaction from treatment requiring withdrawal from the trial was mild paroxysmal supraventricular tachycardia.
Parle et al. [71] (94; 52 vs. 42)	Control group (6; 14.3% vs. treatment group (5; 9.6%)	OR: 0.64 (95% CI 0.18–2.26); *p* ≥ 0.05 Absolute value (range): 46 fewer per 1000 (from 114 fewer to 131 more) The side effects resulting in withdrawal from the trial were not described.
Teixeira et al. [74, 75] (60; 35 vs. 25)	Control group (0; 0.0% vs. treatment group (2; 5.7%)	OR: 3.81 (95% CI 0.17–82.80); *p* ≥ 0.05 Absolute value (range): 0 fewer per 1000 (from 0 fewer to 0 fewer) Adverse events requiring withdrawal from the trial included developing “hashitoxicosis” while on levothyroxine therapy and symptomatic tachycardia
Zhao et al. [77] (369; 210 vs. 159)	Control group (0; 0.0%) vs. treatment group (0; 0.0%)	No withdrawals due to adverse effects were reported in either the control or treatment groups.

***KQ5: What are asymptomatic, non-pregnant adults’ preferences and values concerning screening for TD?***

No studies reporting on asymptomatic, non-pregnant adults’ preferences and values concerning screening for TD were found.

***KQ6: If screening asymptomatic, non-pregnant adults for TD is clinically effective, then what is the cost-effectiveness and associated resource use?***

This systematic review did not find any studies reporting on the clinical effectiveness of screening asymptomatic, non-pregnant adults for TD. Therefore, a systematic search for evidence to answer this key question was not conducted.

## Discussion

No evidence on the benefits and harms of screening versus not screening asymptomatic non-pregnant adults for TD were found. Similarly, no studies reporting on the benefits and harms of treatment compared to no treatment for screen-detected overt thyroid disease, or subclinical hyperthyroidism, in asymptomatic non-pregnant adults were found. In addition, no studies reporting on patients’ preferences and values towards screening for TD were found. All of the included RCTs and cohort studies in this review reported on the benefits and harms of treating asymptomatic non-pregnant adults with subclinical hypothyroidism compared to no treatment (i.e., placebo or observation).

Most (if not all, depending on the outcome) of the RCTs and cohort studies that reported on the clinically important outcomes considered in this review found very small effect sizes that were not statistically significant. Although both retrospective cohort studies [78, 80] that considered the outcome of all-cause mortality among adults < 65 or 40–70 years of age reported a statistically significant reduction among those treated for subclinical hypothyroidism, the overall quality of the evidence was assessed as very low, meaning that there is large uncertainty around this effect. On the other hand, there is moderate certainty that the differences in effect estimates between those treated or not treated for subclinical hypothyroidism are not statistically significant for: all-cause mortality in older adults, occurrence of fatal and non-fatal cardiovascular events, atrial fibrillation, and measures of QoL.

There is moderate certainty that the differences in effects between those treated and not treated for subclinical hypothyroidism on intermediate outcomes are very small and not statistically significant. Although pre-determined clinically meaningful differences between the two groups were not available, the differences found in this review appear to be minimal when considering the range of possible values and what would be considered within normal range for the intermediate outcomes.

Across studies (one RCT [73] and three cohort studies [78–80]), subgroup analyses based on age and gender predominantly found no statistically significant differences, and none that were clinically important, between those treated and not treated for subclinical hypothyroidism on mortality and cardiovascular events. Although increasing age or being female are risk factors for TD, the overall evidence does not support that these high prevalence groups benefit more from treatment compared to adults < 65 or ≤ 70 years of age or males. The quality of evidence for these subgroup analyses was very low (for adults < 65 or ≤ 70 years of age, females, and males) or moderate (for adults ≥ 65 or > 70 years of age).

The findings on treatment effectiveness from RCTs that included participants from population-based screening approaches [62, 63, 71, 77] were very similar to findings from other RCTs included in this review that used alternative screening strategies. These similarities in results suggest that the population included in our treatment review closely resembles asymptomatic non-pregnant adult patients who would have been identified via population-based screening.

There are a few differences between our review and the USPSTF review from 2015 [50]. The USPSTF review included two RCTs [83, 84] that reported on the effects of treatment for subclinical hyperthyroidism. However, these RCTs were excluded from this review because all of the trial participants or all the patients in the treatment group either had Graves’ disease, multinodular goiter or autonomous nodules. Additionally, three studies on treatment for subclinical hypothyroidism that were included in the USPSTF review were excluded from this review because the majority of the trial population were symptomatic [85, 86] or because data on outcomes of interest for the treatment and placebo groups prior to cross-over were not provided in the published paper [87]. An update of the USPSTF search strategy, along with conducting a forward citation search on all of the USPSTF included studies, resulted in this review including 12 additional studies (10 RCTs [59, 61, 64, 65, 67, 70, 72, 73, 76, 77] and two [78, 79] cohort studies) that were not in the USPSTF review. An overall quality (or certainty) assessment of the body of evidence for each outcome using GRADE was also provided in this review [46].

Two recent systematic reviews with meta-analyses [88, 89] examined the effects of treatment for subclinical hypothyroidism on lipid levels. One review [89] included symptomatic and asymptomatic patients in the meta-analyses while the other review [88] included asymptomatic patients only. Unlike the findings from this review, both reviews found a small but statistically significant benefit of treatment on TC and LDL levels. In comparison, the present review included between nine to 10 RCTs for those same outcomes, risk of bias was assessed for each RCT, and an overall GRADE rating was provided for each outcome. Another recent systematic review and meta-analysis [90] examined the effects of treatment for subclinical hypothyroidism but did not find improvements in thyroid-related symptoms or quality of life.

### Limitations

Although the majority of the RCTs included in this review were at low risk of bias for blinding of participants and personnel and blinding of outcome assessment, the majority had unknown risk of bias for sequence generation, allocation concealment, and other biases which could have potentially biased the findings towards a treatment effect. However, most of the RCT outcome measures were not statistically significant. In addition, the findings from RCTs with a high risk of bias in one or more of the risk of bias domains assessed had similar findings to RCTs with either a low risk of bias across all domains or without a high risk of bias assessed in any domain. This suggests that any potential biases may not have influenced trial results. Therefore when the overall risk of bias for each outcome was assessed, no serious concerns were found that would warrant downgrading a full point for the risk of bias domain in GRADE.

Most of the studies included in this review had small sample sizes that may not have provided sufficient power to detect meaningful differences, small effect sizes or rare events. This is evident in the wide CIs. In addition, most of the RCTs had follow-up periods of 12 months or less, which may not have been sufficient to observe changes in long-term clinical outcomes. Also, all but one RCT [60] had participants with mean TSH levels at baseline that were < 10 mIU/L. Clinical recommendations suggest that only individuals with TSH levels > 10 mIU/L be treated for subclinical hypothyroidism [20] suggesting that the majority of participants in the included studies would not have been offered treatment outside of a trial if that clinical recommendation was followed. Therefore, they could be considered overtreated. The findings from this review may not be generalizable to asymptomatic subclinical hypothyroid patients with TSH levels > 10 mIU/L.

Searches only included English language articles for the key questions on the benefits and harms of screening and treatment and only English and French articles on the search for literature on patients’ preferences and values, so it is possible relevant articles written in other languages may have been missed. However, since publication bias is towards reporting of statistically significant findings in support of generally well-accepted treatment interventions (such as levothyroxine for treatment of subclinical hypothyroidism) and because the majority of our findings were not statistically significant, publication bias is unlikely for this review. A forward citation search on all of the 17 studies included in the 2014 USPSTF review, and a bibliographic search from relevant systematic reviews were conducted to identify other potential studies for inclusion. This provides assurance that the studies found in this review represent the current literature available.

### Future research

This review found no studies assessing the effectiveness and harms of screening asymptomatic, non-pregnant adults for TD, or on their preferences and values around being screened for TD. Although research on these areas would be beneficial to inform recommendations on screening, this may be unnecessary unless there are well-designed studies with sufficient power to detect clinically meaningful benefits of treating asymptomatic non-pregnant adults for TD. In particular, there is a lack of sufficiently powered RCTs examining the long-term clinical benefits of early treatment for overt or subclinical hypo- or hyperthyroidism on mortality and cardiovascular outcomes.

There are very few studies reporting on the burden of TD in Canada, each with its own limitations. Currently, limited information is available on the prevalence and incidence of the disease in the Canadian population. Without this information, it would be difficult to assess the potential impact of any population-based preventive intervention for Canadians.

## Conclusion

This review provides a synthesis of the evidence regarding the benefits and harms of screening asymptomatic non-pregnant adults for TD, the benefits and harms of treatment, and on patients’ values and preferences towards TD screening, though this review only found data on the benefits and harms of treatment for subclinical hypothyroidism. This review did not find evidence of treatment benefits for any other outcomes except for very low-quality evidence showing a reduction in all-cause mortality from treatment for the age groups < 65 years and 40–70 years. In addition, this review did not find an increase in the odds of adverse outcomes due to treatment. Given that TSH tests are widely conducted and that the use of thyroid replacement hormones is prevalent in the Canadian population, having high-quality evidence from well-designed trials (i.e., those with sufficient power to detect clinically meaningful effects and conducted over several years to observe long-term clinical outcomes) on the benefits of treatment of TD in screen-detected asymptomatic individuals is critical to inform future recommendations on screening.

## Supplementary information

**Additional file 1:** Appendices 1-11.

**Additional file 2:** Evidence Sets 1-3 contain GRADE Summary of Findings Tables and GRADE Evidence Profile Tables for KQ3a, KQ3b, and KQ4.

## Data Availability

The data analyzed during the current study are available from the corresponding author on reasonable request.

## Abbreviations

AE: Adverse events
BMI: Body mass index
CI: Confidence interval
DBP: Diastolic blood pressure
GRADE: Grading of Recommendations Assessment, Development and Evaluation
HDL: High-density lipoprotein
HR: Hazard ratio
IRR: Incidence rate ratio
KQ: Key question
LDL: Low-density lipoprotein
MD: Mean difference
mU/L: Milliunits per liter
OR: Odds ratio
QoL: Quality of life
RCT: Randomized controlled trial
SBP: Systolic blood pressure
T_3_: Serum triiodothyronine
T_4_: Thyroxine
Task Force: Canadian Task Force on Preventive Health Care
TC: Total cholesterol
TD: Thyroid dysfunction
TG: Triglycerides
TSH: Thyroid-stimulating hormone
USPSTF: US Preventive Services Task Force

## Footnotes

1 An active beneficiary is an individual with at least one claim accepted by the public drug program, either for reimbursement or applied towards a deductible. In most provinces, individuals 65 years and older account for 49–82% of all active beneficiaries of the provincial public drug program [41].

2 The extent of overlap between the cohorts in Andersen 2015 (*n* = 12,212) and Andersen 2016 (*n* = 1192) is unknown. Andersen [78] included participants with or without heart disease living in Copenhagen and had a TSH test conducted between 2000 and 2009, while Andersen [79] only included participants with known heart disease living in Copenhagen, Roskidle, and North Region and had a TSH test conducted between 1997 and 2011.

3 Represents the total number of participants in the study because *n* for the outcome was not provided in the published paper.

4 Represents the total number of female or male participants in the study because *n* for the outcome was not provided in the published paper.

5 Standard definitions are those recommended in the International Conference on Harmonization’s Harmonized Tripartite guideline on clinical safety data management [81].
