# Supplementary material for: Screening for thyroid dysfunction and treatment of screen-detected thyroid dysfunction in asymptomatic, community-dwelling adults: a systematic review
Source: Syst Rev. 2019 Nov 18;8:260. doi: 10.1186/s13643-019-1181-7 (PMC6859607; doi:10.1186/s13643-019-1181-7)
Supplement: Supplementary file 1 — Additional file 1: Appendices 1-11. [file 13643_2019_1181_MOESM1_ESM.docx]

Appendices

[Appendix 1: Search Strategy 2](#_Toc532829296)

[Appendix 2: Forward Citation Search Results 8](#_Toc532829297)

[Appendix 3. Outcome definitions and ratings 12](#_Toc532829298)

[Appendix 4. Additional information used for interpretation of findings by outcome and any clinically important thresholds used to assess the GRADE domain of imprecision for this review 14](#_Toc532829299)

[Appendix 5: List of Excluded Studies for KQ1-4 after Full-text Screening 20](#_Toc532829300)

[Appendix 6: List of Excluded Studies for KQ5 after Full-text Screening 54](#_Toc532829301)

[Appendix 7: Individual Study Characteristics 55](#_Toc532829302)

[Appendix 8. Individual Study Results 63](#_Toc532829303)

[Appendix 9. Risk of Bias Assessments 72](#_Toc532829304)

[Appendix 10. Measures of Quality of Life and Cognitive Function 74](#_Toc532829305)

Appendix 11. Author Correspondence…………………………………………………………………………………………………72

# Appendix 1: Search Strategy

**Limits:**

July 2014 to present (Searches #1-4 were executed on May 04 2017 and updated July 24 2018)

**Search strategies:**

Search #1 – KQ1, KQ2 (Screening)

- Medline and Cochrane

Search #2 – KQ3, KQ4 (Treatment)

- Medline and Cochrane

Search #3 – All key questions

- Medline and Cochrane

Search #4 – KQ5 (Values)

- Medline, Embase, ProQuest Public Health, Scopus

________________________________________________________________________

**Search #1 – KQ1, KQ2 (Screening)**

Database(s): Ovid MEDLINE(R) Epub Ahead of Print, In-Process & Other Non-Indexed Citations, Ovid MEDLINE(R) Daily, Ovid MEDLINE and Versions(R) 
Search Strategy:

| **#** | **Searches** |
| --- | --- |
| 1 | thyroid diseases/ or hyperthyroidism/ or hypothyroidism/ |
| 2 | (thyroid and disease$).mp. |
| 3 | (hypothyroid$ or hyperthyroid$).mp. |
| 4 | or/1-3 |
| 5 | Mass Screening/ |
| 6 | 4 and 5 |
| 7 | Pregnancy/ |
| 8 | (pediatric$ or newborn or neonat$ or child$ or infan$).mp. |
| 9 | 6 not (7 or 8) |
| 10 | limit 9 to (abstracts and english language and humans) |
| 11 | (201407$ or 201408$ or 201409$ or 201410$ or 201411$ or 201412$ or 2015* or 2016* or 2017* or 2018*.).dc,ed. |
| 12 | 10 and 11 |
| 13 | remove duplicates from 12 |

Cochrane Library:

ID Search Hits

#1 MeSH descriptor: [Thyroid Diseases] explode all trees

#2 (thyroid and disease$) (Word variations have been searched)

#3 (hypothyroid$ or hyperthyroid$) (Word variations have been searched)

#4 #1 or #2 or #3

#5 MeSH descriptor: [Mass Screening] this term only

#6 screen$ (Word variations have been searched)

#7 #5 or #6

#8 #4 and #7

#9 #4 and #7 Online Publication Date from Jul 2014 to Jul 2018

________________________________________________________________________

**Search #2 – KQ3, KQ4 (Treatment)**

Database(s): Ovid MEDLINE(R) Epub Ahead of Print, In-Process & Other Non-Indexed Citations, Ovid MEDLINE(R) Daily, Ovid MEDLINE and Versions(R) 
Search Strategy:

| **#** | **Searches** |
| --- | --- |
| 1 | Thyroid Diseases/ |
| 2 | Hyperthyroidism/ |
| 3 | Hypothyroidism/ |
| 4 | (hyperthyroid$ or hypothyroid$).ti. |
| 5 | (thyroid deficien* or thyroid insufficien* or thyroid failure).mp. |
| 6 | or/1-5 |
| 7 | exp Antithyroid Agents/ |
| 8 | (anti-thyroid or methimazole or propylthiouracil or radioiodine or radioactive iodine).ti,ab. |
| 9 | exp Thyronines/ |
| 10 | exp Thyroxine/ |
| 11 | (t3 or t4 or thyroxine or levothyroxine or triiodothyronine or liothyronine or thyrolar or liotrix).ti,ab. |
| 12 | or/7-11 |
| 13 | 6 and 12 |
| 14 | 6 and pc.fs. |
| 15 | 6 and dt.fs. |
| 16 | 6 and th.fs. |
| 17 | or/14-16 |
| 18 | 13 or 17 |
| 19 | Pregnancy/ |
| 20 | (child$ or pediatric$ or infan$ or newborn or neonat$ or toddler).mp. |
| 21 | grave's.ti. |
| 22 | 18 not (19 or 20 or 21) |
| 23 | limit 22 to (abstracts and english language and humans) |
| 24 | limit 23 to (clinical trial, all or comparative study or controlled clinical trial or meta analysis or randomized controlled trial or systematic reviews) |
| 25 | (201407$ or 201408$ or 201409$ or 201410$ or 201411$ or 201412$ or 2015* or 2016* or 2017* or 2018*).dc,ed. |
| 26 | 24 and 25 |
| 27 | remove duplicates from 26 |

Cochrane Library:

ID Search Hits

#1 MeSH descriptor: [Thyroid Diseases] this term only

#2 MeSH descriptor: [Hyperthyroidism] this term only

#3 MeSH descriptor: [Hypothyroidism] this term only

#4 (hyperthyroid$ or hypothyroid$):ti

#5 ("thyroid deficien*" or "thyroid insufficien*" or "thyroid failure")

#6 #1 or #2 or #3 or #4 or #5

#7 MeSH descriptor: [Antithyroid Agents] explode all trees

#8 (anti-thyroid or methimazole or propylthiouracil or radioiodine or "radioactive iodine"):ti,ab

#9 MeSH descriptor: [Thyronines] explode all trees

#10 MeSH descriptor: [Thyroxine] explode all trees

#11 (t3 or t4 or thyroxine or levothyroxine or triiodothyronine or liothyronine or thyrolar or liotrix):ti,ab

#12 #7 or #8 or #9 or #10 or #11

#13 #6 and #12

#14 MeSH descriptor: [Pregnancy] this term only

#15 (child$ or pediatric$ or infan$ or newborn or neonat$ or toddler)

#16 grave's:ti

#17 #13 not (#14 or #15 or #16)

#18 #13 not (#14 or #15 or #16) Online Publication Date from Jul 2014 to Jul 2018

________________________________________________________________________

**Search #3 – All key questions**

Database(s): Ovid MEDLINE(R) Epub Ahead of Print, In-Process & Other Non-Indexed Citations, Ovid MEDLINE(R) Daily, Ovid MEDLINE and Versions(R) 
Search Strategy:

| **#** | **Searches** |
| --- | --- |
| 1 | Thyroid Diseases/ |
| 2 | Hyperthyroidism/ |
| 3 | Hypothyroidism/ |
| 4 | (hyperthyroid$ or hypothyroid$).ti. |
| 5 | (thyroid deficien* or thyroid insufficien* or thyroid failure).mp. |
| 6 | or/1-5 |
| 7 | exp Antithyroid Agents/ |
| 8 | exp Thyronines/ |
| 9 | exp Thyroxine/ |
| 10 | (t3 or t4 or thyroxine or levothyroxine or triiodothyronine or liothyronine or thyrolar or liotrix).ti,ab. |
| 11 | or/7-10 |
| 12 | 6 and 11 |
| 13 | 12 not (pregnan$ or pediatric$ or newborn or neonat$ or child$ or infan$).ti. |
| 14 | limit 13 to systematic reviews |
| 15 | (201407$ or 201408$ or 201409$ or 201410$ or 201411$ or 201412$ or 2015* or 2016* or 2017* or 2018*).dc,ed. |
| 16 | 14 and 15 |
| 17 | remove duplicates from 16 |

Cochrane Library:

ID Search Hits

#1 (thyroid or hypothyroid$ or hyperthyroid$):ti

#2 pregnan$.ti

#3 #1 not #2 Reviews

#4 #1 not #2 Online Publication Date from Jul 2014 to Jul 2018

________________________________________________________________________

**Search #4 – KQ5 (Values)**

Database(s): Ovid MEDLINE(R) Epub Ahead of Print, In-Process & Other Non-Indexed Citations, Ovid MEDLINE(R) Daily, Ovid MEDLINE and Versions(R) 
Search Strategy:

| **#** | **Searches** |
| --- | --- |
| 1 | exp Thyroid Diseases/co, di, pc, px, us [Complications, Diagnosis, Prevention & Control, Psychology, Ultrasonography] |
| 2 | Thyroid Function Tests/ |
| 3 | (hyperthyroid* or hypothyroid* or hyperthyroxinemia or thyrotoxicos*).mp. |
| 4 | (thyroid adj3 (dysfunct* or dysgenes* or disorder* or deficien* or abnormal* or disease*)).mp. |
| 5 | or/1-4 |
| 6 | *patient satisfaction/ or *patient preference/ |
| 7 | *Patient Compliance/px, sn [Psychology, Statistics & Numerical Data] |
| 8 | exp *Attitude to Health/ |
| 9 | (patient adj3 (prefer* or value* or attitude or compliance or satisf* or response*)).mp. |
| 10 | or/6-9 |
| 11 | *Mass Screening/ |
| 12 | (thyroid adj10 (screen* or test*)).mp. |
| 13 | or/11-12 |
| 14 | 5 and 10 and 13 |
| 15 | (pregnan* or infant or newborn).mp. |
| 16 | 14 not 15 |
| 17 | limit 16 to (english or french) |
| 18 | remove duplicates from 17 |

Database(s): **Embase**1974 to 2018 July 24 
Search Strategy:

| **#** | **Searches** |
| --- | --- |
| 1 | exp thyroid disease/co, di, pc [Complication, Diagnosis, Prevention] |
| 2 | exp thyroid function test/ |
| 3 | (hyperthyroid* or hypothyroid* or hyperthyroxinemia or thyrotoxicos*).mp. |
| 4 | (thyroid adj3 (dysfunct* or dysgenes* or disorder* or deficien* or abnormal* or disease*)).mp. |
| 5 | or/1-4 |
| 6 | exp *patient satisfaction/ |
| 7 | exp *patient preference/ |
| 8 | *patient compliance/ or *mental compliance/ |
| 9 | exp *attitude to health/ |
| 10 | (patient adj3 (prefer* or value* or attitude or compliance or satisf* or response*)).mp. |
| 11 | or/6-10 |
| 12 | *mass screening/ |
| 13 | (thyroid adj10 (screen* or test*)).mp. |
| 14 | or/12-13 |
| 15 | 5 and 11 and 14 |
| 16 | (pregnan* or infant or newborn).mp. |
| 17 | 15 not 16 |
| 18 | limit 17 to (english or french) |
| 19 | remove duplicates from 18 |

**ProQuest Public Health Database**

(mesh.Exact("Thyroid Diseases") OR mesh.Exact("Thyroid Function Tests") OR ALL(hyperthyroid* OR hypothyroid* OR hyperthyroxinemia OR thyrotoxicos*) OR ALL(thyroid NEAR/3 (dysfunct* OR dysgenes* OR disorder* OR deficien* OR abnormal* OR disease*))) AND (mesh.Exact("Patient Preference" OR "Patient Satisfaction" OR "Attitude to Health" OR "Patient Compliance") OR (patient NEAR/3 (prefer* OR value* OR attitude OR compliance OR satisf* OR response*))) AND (mesh.Exact("Mass Screening") OR ALL(thyroid NEAR/10 (screen* OR test*))) NOT ALL(pregnan* OR infant OR newborn) AND PEER(yes)

**Scopus**

( ( TITLE-ABS ( thyroid W/3 ( dysfunct* OR dysgenes* OR disorder* OR deficien* OR abnormal* OR disease* OR screen* OR test* ) ) OR TITLE-ABS ( hyperthyroid* OR hypothyroid* OR hyperthyroxinemia OR thyrotoxicos* ) ) AND ALL ( inciden* OR "new* diagnosis" ) AND TITLE-ABS-KEY ( patient W/3 ( prefer* OR value* OR attitude OR compliance OR satisf* OR response* ) ) AND TITLE ( screen* OR test* ) AND NOT ( TITLE-ABS-KEY ( pregnan* OR infant OR newborn ) ) AND ( LANGUAGE ( english OR french ) ) ) AND ( LIMIT-TO ( DOCTYPE , "ar " ) OR LIMIT-TO ( DOCTYPE , "re " ) )

# Appendix 2: Forward Citation Search Results

USPSTF Review on Thyroid Dysfunction Screening and Treatment (<https://www.ncbi.nlm.nih.gov/books/NBK285869/pdf/Bookshelf_NBK285869.pdf>)

Searches executed on May 10 2017 and updated July 24 2018 in Scopus

**Included studies:**

9 documents have cited:

Abu-Helalah M, Law MR, Bestwick JP, Monson JP, Wald NJ. A randomized double-blind crossover trial to investigate the efficacy of screening for adult hypothyroidism. *J Med Screen.* 2010;17(4):164-9.

11 documents have cited:

Andersen MN, Olsen A-S, Madsen JC, Faber J, Torp-Pedersen C, Gislason GH, et al. Levothyroxine substitution in patients with subclinical hypothyroidism and the risk of myocardial infarction and mortality. PLoS ONE 2015;10(6)

8 documents have cited:

Andersen MN, Olsen A-S, Madsen JC, Kristensen SL, Faber J, Torp-Pedersen C, et al. Long-term outcome in levothyroxine treated patients with subclinical hypothyroidism and concomitant heart disease. J Clin Endocrinol Metab 2016;101(11):4170-4177

20 documents have cited:

Buscemi S, Verga S, Cottone S, Andronico G, D’Orio L, Mannino V, et al. Favorable clinical heart and bone effects of anti-thyroid drug therapy in endogenous subclinical hyperthyroidism. *J Endocrinol Invest.* 2007;30(3):230-5.

19 documents have cited:

Cabral MD, Teixeira P, Soares D, Leite S, Salles E, Waisman M. Effects of thyroxine replacement on endothelial function and carotid artery intima-media thickness in female patients with mild subclinical hypothyroidism. *Clinics (Sao Paulo, Brazil)*. 2011;66(8):1321-8.

205 documents have cited:

Caraccio N, Ferrannini E, Monzani F. Lipoprotein profile in subclinical hypothyroidism: response to levothyroxine replacement, a randomized placebo-controlled study. *J Clin Endocrinol Metab.* 2002;87(4):1533-38.

63 documents have cited:

Caraccio N, Natali A, Sironi A, et al. Muscle metabolism and exercise tolerance in subclinical hypothyroidism: A controlled trial of levothyroxine. J Clin Endocrinol Metab. 2005;90(7):4057-4062.

12 documents have cited:

Duman D, Sahin S, Esertas K, Demirtunc R. Simvastatin improves endothelial function in patents with subclinical hypothyroidism. *Heart Vessels.* 2007;22(2):88-93.

12 documents have cited:

Fadeyev VV, Sytch J, Kalashnikov V, Rojtman A, Syrkin A, Melnichenko G. Levothyroxine replacement therapy in patients with subclinical hypothyroidism and coronary artery disease. Endocrine Practice 2006;12(1):5-17

109 documents have cited:

Iqbal A, Jorde R, Figenschau Y. Serum lipid levels in relation to serum thyroid-stimulating hormone and the effect of thyroxine treatment on serum lipid levels in subjects with subclinical hypothyroidism: the Tromsø Study. *J Intern Med.* 2006;260(1):53-61.

140 documents have cited:

Jorde R, Waterloo K, Storhaug H, Nyrnes A, Sundsfjord J, Jenssen TG. Neuropsychological function and symptoms in subjects with subclinical hypothyroidism and the effect of thyroxine treatment. *J Clin Endocrinol Metab.* 2006;91(1):145-53.

155 documents have cited:

Kong WM, Sheikh MH, Lumb PJ, Naoumova RP, Freedman DB, Crook M, et al. A 6-month randomized trial of thyroxine treatment in women with mild subclinical hypothyroidism. *Am J Med.* 2002;112(5):348-54.

6 documents have cited:

Liu P, Liu R, Chen X, Chen Y, Wang D, Zhang F, et al. Can levothyroxine treatment reduce urinary albumin excretion rate in patients with early type 2 diabetic nephropathy and subclinical hypothyroidism? A randomized double-blind and placebo-controlled study. Current Medical Research & Opinion 2015 Dec;31(12):2233-2240

19 documents have cited:

Mainenti MRM, Vigário PS, Teixeira PFS, Maia MDL, Oliveira FP, Vaisman M. Effect of levothyroxine replacement on exercise performance in subclinical hypothyroidism. J Endocrinol Invest 2009;32(5):470-473

18 documents have cited:

Mikhail GS, Alshammari SM, Alenezi MY, Mansour M, Khalil NA. Increased atherogenic low-density lipoprotein cholesterol in untreated subclinical hypothyroidism. *Endocr Pract.* 2008;14(5):570-5.

221 documents have cited:

Monzani F, Di Bello V, Caraccio N, et al. Effect of levothyroxine on cardiac function and structure in subclinical hypothyroidism: A double blind, placebo-controlled study. J Clin Endocrinol Metab. 2001;86(3):1110-1115.

249 documents have cited:

Monzani F, Caraccio N, Kozàkowà M, Dardano A, Vittone F, Virdis A, et al. Effect of levothyroxine replacement on lipid profile and intima-media thickness in subclinical hypothyroidism: a double-blind, placebo-controlled study. *J Clin Endocrinol Metab.* 2004;89(5):2099-106.

24 documents have cited:

Nagasaki T, Inaba M, Yamada S, Shirakawa K, Nagata Y, Kumeda Y, et al. Decrease of brachial-ankle pulse wave velocity in female subclinical hypothyroid patients during normalization of thyroid function: a double-blind, placebo-controlled study. *Eur J Endocrinol.* 2009;160(3):409-15.

4 documents have cited:

Najafi L, Malek M, Hadian A, Ebrahim Valojerdi A, Khamseh ME, Aghili R. Depressive symptoms in patients with subclinical hypothyroidism--the effect of treatment with levothyroxine: a double-blind randomized clinical trial. Endocr Res 2015;40(3):121-126

89 documents have cited:

Parle J, Roberts L, Wilson S, Pattison H, Roalfe A, Haque MS, et al. A randomized controlled trial of the effect of thyroxine replacement on cognitive function in community-living elderly subjects with subclinical hypothyroidism: the Birmingham Elderly Thyroid study. *J Clin Endocrinol Metab.* 2010;95(8):3623-32.

263 documents have cited:

Razvi S, Ingoe L, Keeka G, Oates C, McMillan C, Weaver JU. The beneficial effect of L-thyroxine on cardiovascular risk factors, endothelial function, and quality of life in subclinical hypothyroidism: randomized, crossover trial. *J Clin Endocrinol Metab.* 2007;92(5):1715-23.

112 documents have cited:

Razvi S, Weaver JU, Butler TJ, Pearce SH. Levothyroxine treatment of subclinical hypothyroidism, fatal and nonfatal cardiovascular events, and mortality. *Arch Intern Med.* 2012;172(10):811-7.

15 documents have cited:

Reuters VS, Almeida CP, Teixeira PFS, Vigário PS, Ferreira MM, de Castro CLN, et al. Effects of subclinical hypothyroidism treatment on psychiatric symptoms, muscular complaints, and quality of life. Arq Bras Endocrinol Metabol 2012;56(2):128-136

44 documents have cited:

Stott DJ, Rodondi N, Kearney PM, Ford I, Westendorp RG, Mooijaart SP, et al. Thyroid Hormone Therapy for Older Adults with Subclinical Hypothyroidism. N Engl J Med 2017 Apr 3

48 documents have cited:

Teixeira PF, Reuters VS, Ferreira MM, Almeida CP, Reis FA, Buescu A, et al. Lipid profile in different degrees of hypothyroidism and effects of levothyroxine replacement in mild thyroid failure. *Transl Res.* 2008;151(4):224-31.

29 documents have cited:

Teixeira PF, Reuters VS, Ferreira MM, Almeida CP, Reis FA, Melo BA, et al. Treatment of subclinical hypothyroidism reduces atherogenic lipid levels in a placebo-controlled double-blind clinical trial. *Horm Metab Res.* 2008;40(1):50-5.

66 documents have cited:

Yazici M, Gorgulu S, Sertbas Y, et al. Effects of thyroxin therapy on cardiac function in patients with subclinical hypothyroidism: Index of myocardial performance in the evaluation of left ventricular function. Int J Cardiol. 2004;95(2-3):135-143.

22 documents have cited:

Yonem O, Dokmetas HS, Aslan SM, Erselcan T. Is antithyroid treatment really relevant for young patients with subclinical hyperthyroidism? *Endocrine.* 2002;49(3):307-14.

4 documents have cited:

Zhao M, Liu L, Wang F, Yuan Z, Zhang X, Xu C, et al. A Worthy Finding: Decrease in Total Cholesterol and Low-Density Lipoprotein Cholesterol in Treated Mild Subclinical Hypothyroidism. Thyroid 2016;26(8):1019-1029

**In total:**

After removing duplicates: 1147 references

# Appendix 3. Outcome definitions and ratings

| **Outcome** | **Definition** | **Mean rating score** | **Final rating** |
| --- | --- | --- | --- |
| **Clinical Outcomes** | | | |
| **Mortality** | Death | 8.75 | Critical |
| All-cause mortality | Death from any cause | 7.5 | Critical |
| Death due to cardiovascular diseases | Death from heart disease or stroke. | 8.75 | Critical |
| **Cardiovascular events** | Any event that causes damage to the heart muscle or blood vessels. This includes heart disease/failure or stroke, and atrial fibrillation. | 7.5 | Critical |
| Fatal and non-fatal cardiovascular events | Any event that causes damage to the heart muscle or blood vessels. This includes heart disease or stroke. | 7.75 | Critical |
| Atrial fibrillation | Abnormal heart beat which can increase the risk of heart disease and stroke. | 7.75 | Critical |
| **Fractures** | Major bone fracture (break). May be caused by weakening of the bones due to osteoporosis. | 7.5 | Critical |
| **Quality of life** | A person's overall well-being (includes physical and mental well-being) and ability to enjoy normal life activities. | 7 | Critical |
| Thyroid-specific QoL | Quality of life related to thyroid disease symptoms (e.g. fatigue, depression, anxiety, weight gain/loss) | 7 | Critical |
| Mental well-being | A person’s psychological and emotional well-being. | 6.75 | Important |
| Physical well-being | The general good health of the physical body (absence of disease). | 6.5 | Important |
| General well-being | A person's overall physical, mental, and social well-being | 6.75 | Important |
| Fatigue/tiredness | Tiredness or extreme tiredness (fatigue), typically resulting from mental or physical exertion or illness. | 6.67 | Important |
| **Cognitive function** | Mental functions that help us gather and process information. This includes memory, attention, language processing, decision-making and reasoning. | 7.25 | Critical |
| **Intermediate Outcomes** | | | |
| **Bone mineral density** | Bone mineral density (BMD) is a measure of the amount of minerals (e.g. calcium) in your bones. Low calcium levels may indicate osteoporosis or weakening of the bones. BMD is measured by X-ray of the hip and spine. | 5.25 | Important |
| **Cholesterol/Lipid Levels** | A measurement of the cholesterol and lipid (fat) levels in the blood. This can be used to identify your risk of coronary heart disease. Includes measures of total cholesterol, low-density lipoprotein, high-density lipoprotein, and triglyceride levels in the blood. | 4.75 | Important |
| **Blood Pressure** | Blood pressure is the strength of your blood pushing against the sides of your blood vessels. High blood pressure means that your heart is working harder to pump blood and it is a risk factor for cardiovascular disease. Includes measures for systolic and diastolic blood pressure. | 5.75 | Important |
| **Weight Change** | A change in body weight due to increased or decreased fat deposits, muscle mass or fluid, usually measured as changes in body mass index (BMI). There are many health effects associated with high weight gain and loss. | 4.5 | Important |
| **Harms** | | | |
| **Harms due to Treatment** | Experiencing unpleasant or harmful side effects or symptoms due to treatment | 7 | Critical |
| **Harms Due to Screening** | Adverse events due to the screening test and subsequent follow-up and treatment. These may include psychological effects, harms of workup, overdiagnosis and overtreatment. | 6.75 | Important |
| **Patient preferences and values towards screening** | | | |
| **Patient Values and Preferences for Screening** | Patient views of the relative benefits and harms of screening, and what factors patients consider when deciding whether to undergo screening. | N/A | N/A |
| Willingness to be screened | A patient's readiness or interest in being screened. | N/A | N/A |
| Factors considered in decision to be screened | What components of screening do patients place more value on when deciding whether to be screened or not. | N/A | N/A |
| **Cost effectiveness of screening** | | | |
| **Cost effectiveness of screening** | Cost effectiveness analysis compares the relative costs associated with screening to the health outcomes | N/A | N/A |

# Appendix 4. Additional information used for interpretation of findings by outcome and any clinically important thresholds used to assess the GRADE domain of imprecision for this review

**Clinical Outcomes**

| **Clinical outcomes** | **Unit of measurement** | **Range of possible values and/or directionality of effect** | **Clinically-important difference between treated and untreated groups** | **Reference (if available) or justification for clinically important threshold** |
| --- | --- | --- | --- | --- |
| **Mortality** |  |  |  |  |
| Includes all-cause mortality and deaths due to cardiovascular diseases | # events / 1000 | 0 to 1000 | An increase of ≥1 death | Patients will not want to increase their chances of dying |
| **Cardiovascular events:** |  |  |  |  |
| Fatal and non-fatal cardiovascular events (not including atrial fibrillation) | # events / 1000 | 0 to 1000 | An increase of ≥1 event | Patients will not want to increase their chances of dying or experiencing a non-fatal cardiovascular event |
| Atrial fibrillation | # events / 1000 | 0 to 1000 | Could not be determined | The range of severity of symptoms or adverse consequences due to atrial fibrillation events can range from mild to severe. Information on what patients and clinicians would find as a clinically meaningful difference between the treated and untreated groups is lacking. |
| **Fractures** | # events / 1000 | 0 to 1000 | Could not be determined | Information on what patients and clinicians would find as a clinically meaningful difference between the treated and untreated groups is lacking. A large majority of patients (perhaps between 95%-99%) may not want to increase their chances of having a fracture from treatment for thyroid dysfunction. |
| **Quality of life (QoL):** |  |  |  |  |
| Thyroid-related QoL (not including fatigue) and Fatigue/tiredness  *Includes the following tests: Underactive Thyroid-dependent quality of life (ThyPRO) Hypothyroid symptom and fatigue/vitality domains, ThyPRO-39* | Score | 0 to 100 ↓  *(higher scores are associated with poorer QoL)* | ± 9 points | The author of ThyPRO (T. Watt) indicated that a 9 point change would be a realistic and clinically meaningful effect size. [Stott DJ, Gussekloo J, Kearney PM, et al. Study protocol; Thyroid hormone Replacement for Untreated older adults with Subclinical hypothyroidism - a randomised placebo controlled Trial (TRUST). *BMC Endocrine Disorders*. 2017;17:6. doi:10.1186/s12902-017-0156-8.] |
| Mental well-being  *Includes the following tests: Beck Depression Inventory (BDI), Hamilton Scale for anxiety (HAM-A) and depression (HAM-D), hospital anxiety and depression scale (HADS) depression scale only* | Score | Varies by test:  BDI: 0-63 ↓  HAM-A: 0-56 ↓  HAM-B: 0-52 ↓  HADS: 0-21 ↓ | Could not be determined | Information on what patients and clinicians would find as a clinically meaningful difference between the treated and untreated groups for each test is lacking. In addition, because various test results were included in the assessment of this outcome, each with a potentially different clinically meaningful threshold, then a clinically important difference between the treated and untreated groups for this outcome as a whole could not be determined. |
| Physical well-being  *Includes the following tests: Barthel Index – basic activities of daily living (BI-ADL) and the Older American Resources and Services – instrumental activities of daily living (OARS-ADL)* | Score | Varies by test:  BI-ADL: 0-20 ↑  OARS-ADL: 0-14 ↑ | Could not be determined | Information on what patients and clinicians would find as a clinically meaningful difference between the treated and untreated groups for each test is lacking. In addition, because various test results were included in the assessment of this outcome, each with a potentially different clinically meaningful threshold, then a clinically important difference between the treated and untreated groups for this outcome as a whole could not be determined. |
| General well-being  *Includes the following tests: EUROQUOL Group-5 Dimension report questionnaire descriptive score (EQ-5D DES) and visual analogue scale score (EQ-5D VAS),General Health Questionnaire-30 (GHQ-30) and the Medical Outcomes Study 36-item Short Form Health Survey (SF-36)* | Score or standardized combined score | Varies by test:  EQ-5D DES: -0.59-1.0 ↑  EQ-5D VAS: 0-100 ↑  GHQ-30: 0-90 ↓  SF-36: mean of 50, SD of 10 ↑ | Could not be determined, however a minimally important difference for EQ-5D DES was estimated to be between 0.037±0.001 and 0.056±0.011 for Canada | Information on what patients and clinicians would find as a clinically meaningful difference between the treated and untreated groups for each test is lacking, except for EQ-5D DES [McClure NS, Sayah FA, Xie F, Luo N, Johnson JA. Instrument-defined estimates of the minimally important difference for EQ-5D-5L index scores. Value Health. 2017;20(4):644–650]. In addition, because various test results were included in the assessment of this outcome, each with a potentially different clinically meaningful threshold, then a clinically important difference between the treated and untreated groups for this outcome as a whole could not be determined. |
| **Cognitive function** |  |  |  |  |
| Overall cognitive function  *Includes the following tests: California Computerized Assessment Package (CalCAP), Composite cognitive score (CCS), Controlled word association test (CWA), Letter digit coding test (LDC), Middlesex elderly assessment of mental state (MEAMS), Mini-mental state examination (MMSE), Seashore rhythm test (SR), Speed and capacity of language processing test (SCOLP), Trail making test A (TM-A) B (TM-B) and B-A (TM-BA), Vocabulary – Wechsler intelligence scale (VWI) and Word list test (WL)* | Score, discrepancy score or standardized combined score | Varies by test:  CalCAP ↓  CCS ↑^α^  CWA↑  LDC↑  MEAMS: 0-12 ↑  MMSE: 0-30 ↑  SR ↑  SCOLP: -1-12 (≥4 is severe) ↓  TM-A ↓  TM-B ↓  TM-BA ↓  VWI ↑  WL ↑ | Could not be determined | Information on what patients and clinicians would find as a clinically meaningful difference between the treated and untreated groups for each test is lacking. In addition, because various test results were included in the assessment of this outcome, each with a potentially different clinically meaningful threshold, then a clinically important difference between the treated and untreated groups for this outcome as a whole could not be determined. |

↓ higher scores are associated with poorer QoL or cognitive function. ↑ higher scores are associated with better QoL or cognitive function. ^α^Composite score made by adding the z-scores for these 7 tests: Digit Span forward & backward, Stroop parts 1-3, Digit Symbol, verbal and visual recall.

**Intermediate Outcomes**

| **Intermediate outcomes** | **Unit of measurement** | **Standard reference ranges** | **Clinically-important difference between treated and untreated groups** | **Reference (if available)** |
| --- | --- | --- | --- | --- |
| **Cholesterol/Lipid Levels:** | N/A | Cholesterol (LCL, Total, HDL, TG) Normal ranges depend on individual patient factors | Could not be determined | Harrison's Principles of Internal Medicine, 19e, 2015 > Laboratory Values of Clinical Importance. Accessed from: https://accessmedicine.mhmedical.com/ViewLarge.aspx?figid=98720108&gbosContainerID=0&gbosid=0 |
| **Blood Pressure (BP):** |  |  |  | Harrison's Principles of Internal Medicine, 19e, 2015 > Laboratory Values of Clinical Importance. Accessed from: https://accessmedicine.mhmedical.com/content.aspx?bookid=331&sectionid=40727026#9104902 |
| Systolic BP | mm Hg | Hypertension >135 | Could not be determined | Information on what patients and clinicians would find as a clinically meaningful difference between the treated and untreated groups is lacking. |
| Diastolic BP | mm Hg | Hypertension >85 | Could not be determined | Information on what patients and clinicians would find as a clinically meaningful difference between the treated and untreated groups is lacking. |
| **BMI** | kg/m^2^ | Normal: 18.5 to 24.9 | Could not be determined | Information on what patients and clinicians would find as a clinically meaningful difference between the treated and untreated groups is lacking. [Source of standard reference values obtained from Health Canada: https://www.canada.ca/en/health-canada/services/food-nutrition/healthy-eating/healthy-weights/canadian-guidelines-body-weight-classification-adults/quick-reference-tool-professionals.html?wbdisable=true] |

**Harms due to Treatment**

| **Harms due to treatment** | **Unit of measurement** | **Range of possible values** | **Clinically-important difference between treated and untreated groups*** | **Reference (if available) for clinically important threshold** |
| --- | --- | --- | --- | --- |
| Number of individuals reporting adverse outcomes | # outcomes / 1000 | 0 to 1000 | Could not be determined | The range of severity of symptoms or adverse events due to treatment included in this outcomes range from mild to severe. Information on what patients and clinicians would find as a clinically meaningful difference between the treated and untreated groups is lacking. |
| Withdrawals due to adverse outcomes | # withdrawals / 1000 | 0 to 1000 | Could not be determined | Information on what patients and clinicians would find as a clinically meaningful difference between the treated and untreated groups is lacking. A large majority of patients (perhaps between 95%-99%) may not want to experience an adverse event serious enough to require them to withdraw from a trial or discontinue treatment for subclinical hypothyroidism. |

# Appendix 5: List of Excluded Studies for KQ1-4 after Full-text Screening

**Non-English Article : n = 28**

Baghbani-Oskouei A, Akbarpour S, Tohidi M, Amouzegar A, Mehran L, Hadaegh F, Azizi F. Subclinical thyroid dysfunction and incident cardiovascular disease: Tehran thyroid study. Iranian Journal of Endocrinology and Metabolism. 2018. 20:1

De Pedro S, Benozzi S, Becerra H, Bonacorsi SM, Jouffré G, et al. Cardiovascular risk factors in patients with subclinical hypothiroidism. Revista argentina de endocrinologia y metabolismo. 2012. 49:183

Derakhshan S, Shahsavari S. Effect of oral anti-thyroid therapy on bone density in premenopausal women with endogenous subclinical hyperthyroidism. Scientific Journal of Kurdistan University of Medical Sciences. 2014. 19:1

Dominguez LJ, Di Bella G, Damiani P, Belvedere M, Barbagallo M. Hypothyroidism in older persons: Importance of an adequate thyroid hormone replacement therapy. Giornale di gerontologia. 2013. 61:173

Gussekloo J, Van Exel E, De Craen AJM, Meinders AE, Frölich M, et al. Thyroid function, activities of daily living and survival in extreme old age: The 'Leiden 85-plus Study'. Nederlands tijdschrift voor geneeskunde. 2006. 150:90

Hong TP. Reinforcing research on the subclinical state of autoimmune thyroid disease. National Medical Journal of China. 2008. 88:2809

Krysicki M, ͆lusarczyk E, Popowicz B, Jankiewicz-Wika J, Klencki M, et al. Effect of subclinical hypothyroidism treatment on selected cardiovascular parameters. Polski Merkuriusz Lekarski. 2014. 37:17

La Viola M, Greco A, Mazzoccoli G, Carughi S, Puzzolante F, et al. Hemodynamic evaluation by bioimpedance in patients with subclinical hypothyroidism before and after levothyroxine treatment. Recenti progressi in medicina. 2003. 94:549

Lipp RW, Scherer T, Krebs M. Is Thyroxine Therapy Indicated in Any Case of Subclinical Hypothyroidism?. Austrian Journal of Clinical Endocrinology and Metabolism. 2013. 6:12

Loor M, Giet D. Subclinical primary hypothyroidism in family practice. Revue medicale de Liege. 2008. 63:600

Mainenti MRM, P. F. S. Teixeira, F. P. Oliveira, M. Vaisman. Impact of subclinical hypothyroidism in cardiopulmonary response during effort and its recovery. Arquivos Brasileiros de Endocrinologia e Metabologia. 2007. 51:1485

Merchante-Alfaro AÁ, Civera-Andrés M, Atiénzar-Herráez N, Tenías-Burillo JM, Ochoa-Ávila E, et al. Effects of levothyroxine replacement on lipid profile in patients with mild subclinical hypothyroidism. Medicina clinica. 2006. 126:246

Neumann I, -A Juan Carlos Claro G. Surrogate outcomes. Revista medica de Chile. 2012. 140:113

Noguerol álvarez M, Odriozola Sánchez J, Ávila Londoño DA, Corcuera Martínez AI, Rabanal Basalo A, et al. How we treat subclinical hypothyroidism in our daily clinical practice. Semergen. 2012. 38:483

Nyström E, B. Hallengren, G. Lindstedt, B. Winsa. Primary hypothyroidism - An increasingly widespread disease: A common and simple to treat disease in women. Lakartidningen. 2003. 100:610

Önder E, Aydin Y, Güngör A, Celbek G, Kir S, et al. Carotis intima media thickness in female patiens with subclinical hypothyroidism. Turkish Journal of Endocrinology and Metabolism. 2010. 14:89

Peng L, -J Gu M. Influence of thyroxine treatment on serum lipid levels in patients with subclinical hypothyroidism: A meta-analysis. Academic Journal of Second Military Medical University. 2007. 28:519

Rodondi N, D. Nanchen. Medical overconsumption and lipid-lowering treatments in 2018. Revue Medicale Suisse. 2018. 14:451

Rogala N, Zdrojowy-Wełna A, Zatońska K, Bednarek-Tupikowska G. Management of subclinical hypothyroidism in adults. Family Medicine and Primary Care Review. 2015. 17:55

Sampaolo G, Campanella N, Catozzo V, Ferretti M, Vichi G, et al. Relationship between hypothyroidism and cholesterol out of the records of 1756 patients. Recenti progressi in medicina. 2014. 105:79

Scherbaum W, D. Führer. TRUST trial: Differentiated consideration leads to another interpretation. Deutsches Arzteblatt International. 2017. 114:A1052

Schlienger JL, Vinzio S, Grunenberger F, Luca F, Goichot B. Is subclinical hypothyroidism a cardiovascular risk factor?. Revue de Medecine Interne. 2006. 27:927

Sönnichsen A. Replacement therapy with l-thyroxine in subclinical hypothyroidism. Zeitschrift fur Allgemeinmedizin. 2018. 94:51

Van Harten AC, Leue C, Verhey FRJ. Should depressive symptoms in patients with subclinical hypothyroidism be treated with thyroid hormone?. Tijdschrift voor Psychiatrie. 2008. 50:539

Vera-Lastra OL, Medina-García G, Guadalupe-Reséndiz-Pérez L, Ángeles-Garay U, Torres-Ambriz P, et al. Prevalence of hypothyroidism and anti-thyroglobuline antibodies among Mexican patients with systematic sclerosis. Gaceta medica de Mexico. 2007. 143:471

Vinzio S, A. Trinh, J. -L Schlienger, B. Goichot. Cardiac consequences of subclinical dysthyroidism: Experimental, clinical, and epidemiologic data. Presse Medicale. 2005. 34:1161

Yazidi M, M. Chihaoui, I. Mezghani, I. Oueslati, O. Rejeb, F. Chaker, H. Slimane. Metabolic and cardiovascular impact of uncomplicated hypothyroidism: Myth or reality?. Medecine Therapeutique. 2017. 23:288

Yetmiş M, Kazancioǧlu R, Erkoç R, Tükek T, Peru C, et al. Changes in lipid profile and body mass index in patients with subclinical hypothyroidism: Evaluation of L-Thyroxine treatment. Haseki Tip Bulteni. 2011. 49:131

**Wrong study design : n = 205**

Abdel Hamid AmrMS, Borg TamerF, Madkour WaelAI. Prevalence of hyperprolactinemia and thyroid disorders among patients with abnormal uterine bleeding. International Journal of Gynaecology & Obstetrics. 2015. 131:273

Abreu IsabelM,​ Eva Lau,​ Bernardo de Sousa Pinto,​ Davide Carvalho. Subclinical hypothyroidism: to treat or not to treat,​ that is the question! A systematic review with meta-analysis on lipid profile. Endocrine Connections. 2017. 6:188

Aggarwal N, Razvi S. Thyroid and aging or the aging thyroid? An evidence-based analysis of the literature. Journal of Thyroid Research. 2013.

Akter N, Qureshi NK, Ferdous HS. Subclinical hypothyroidism:A review on clinical consequences and management strategies. Journal of Medicine (Bangladesh). 2017. 18:30

Alghalayini K. Prevalence of hypothyroidism in a cohort of saudi women with heart failure and effect on systolic and diastolic function. Journal of the Pakistan Medical Association. 2015. 65:1300

Al-Mahdili HA, Hooper AJ, Sullivan DR, Stewart PM, Burnett JR. A mild case of abetalipoproteinaemia in association with subclinical hypothyroidism. Annals of Clinical Biochemistry. 2006. 43:516

Anagnostis P, Efstathiadou ZA, Slavakis A, Selalmatzidou D, Poulasouchidou M, et al. The effect of L-thyroxine substitution on lipid profile, glucose homeostasis, inflammation and coagulation in patients with subclinical hypothyroidism. International journal of clinical practice. 2014. 68:857

Anagnostis P, Karras SN, Gotsis E, Gouni-Berthold I. Thyroid dysfunction and arterial stiffness. does the restoration of thyroid function tests offer any benefit?. Open Hypertension Journal. 2013. 5:87

Anagnostis P, Karras SN, Katsiki N, Athyros VG, Karagiannis A. The interplay between TSH and lipids. What should clinicians know?. Current Vascular Pharmacology. 2015. 13:91

Arinzon Z, Zuta A, Peisakh A, Feldman J, Berner Y. Evaluation response and effectiveness of thyroid hormone replacement treatment on lipid profile and function in elderly patients with subclinical hypothyroidism. Archives of Gerontology and Geriatrics. 2007. 44:13

Åsvold BO, Bjøro T, Platou C, Vatten LJ. Thyroid function and the risk of coronary heart disease: 12-year follow-up of the HUNT Study in Norway. Clinical endocrinology. 2012. 77:911

Aziz Khan F, Patil SKB, Thakur AS, Fareed Khan M, Murugan K. Lipid profile in thyroid dysfunction: A study on patients of bastar. Journal of Clinical and Analytical Medicine. 2014. 5:12

Bailey ErinB, Tantravahi SrinivasK, Austin Poole, Agarwal ArchanaM, Straubhar AlliM, et al. Correlation of degree of hypothyroidism with survival outcomes in patients with metastatic renal cell carcinoma receiving vascular endothelial growth factor receptor tyrosine kinase inhibitors.. Clinical Genitourinary Cancer. 2015. 13:e131

Baycan S, D. Erdogan, M. Caliskan, B. O. Pamuk, O. Ciftci, H. Gullu, A. Yildirir, N. D. Guvener, H. Muderrisoglu. Coronary flow reserve is impaired in subclinical hypothyroidism. Clinical cardiology. 2007. 30:562

Bello VD. Use of Myocardial Imaging to Identify and Manage Subclinical Heart Disease in Thyroid and Other Endocrine Diseases. Myocardial Imaging: Tissue Doppler and Speckle Tracking. 2008. #volume#:209

Benetti-Pinto CL, Berini Piccolo VRS, Garmes HM, Teatin Juliato CR. Subclinical hypothyroidism in young women with polycystic ovary syndrome: An analysis of clinical, hormonal, and metabolic parameters. Fertility and sterility. 2013. 99:588

Beyhan Z, Ertürk K, Üçkaya G, Bolu E, Yaman H, et al. Restoration of euthyroidism does not improve cardiovascular risk factors in patients with subclinical hypothyroidism in the short term. Journal of endocrinological investigation. 2006. 29:505

Biondi B. Natural history, diagnosis and management of subclinical thyroid dysfunction. Best Practice and Research: Clinical Endocrinology and Metabolism. 2012. 26:431

Blum MR, Bauer DC, Collet TH, Fink HA, Cappola AR, et al. Subclinical thyroid dysfunction and fracture risk: a meta-analysis.. JAMA. 2015. 313:2055.

Boelaert K. Thyroid dysfunction in the elderly. Nature Reviews Endocrinology. 2013. 9:194

Boeving A, Paz-Filho G, Radominski RB, Graf H, De Carvalho GA. Low-normal or high-normal thyrotropin target levels during treatment of hypothyroidism: A prospective, comparative study. Thyroid. 2011. 21:355

Braithwaite SS. Thyroid Disorders. Critical Care Medicine: Principles of Diagnosis and Management in the Adult. 2008. #volume#:1281

Brenta G, Vaisman M, Sgarbi JA, Bergoglio LM, de Andrada NC, et al. Clinical practice guidelines for the management of hypothyroidism. Arquivos Brasileiros de Endocrinologia e Metabologia. 2013. 57:265

Burns RB, Bates CK, Hartzband P, Smetana GW. Should we treat for subclinical hypothyroidism?: Grand rounds discussion from beth Israel deaconess medical center. Annals of Internal Medicine. 2016. 164:764

Cakmak G, T. Saler, Z. A. Saglam, M. Yenigun, E. Ataoglu, T. Demir, L. U. Temiz. Pulmonary functions in patients with subclinical hypothyroidism. Journal of the Pakistan Medical Association. 2011. 61:951

Canat L, Akif Erbin, Masum Canat, Mehmet Dinek, Turhan Caskurlu. Assessment of hormonal activity in patients with premature ejaculation.. International Braz J Urol. 2017. 43:311

Ceresini G, Lauretani F, Maggio M, Ceda GP, Morganti S, et al. Thyroid function abnormalities and cognitive impairment in elderly people: Results of the invecchiare in chianti study. Journal of the American Geriatrics Society. 2009. 57:89

Chaker L, Baumgartner C, den Elzen WP, Ikram MA, Blum MR, et al. Subclinical Hypothyroidism and the Risk of Stroke Events and Fatal Stroke: An Individual Participant Data Analysis. Journal of Clinical Endocrinology & Metabolism. 2015. 100:2181.

Chen D, Y. Yan, H. Huang, Q. Dong, H. Tian. The association between subclinical hypothyroidism and erectile dysfunction. Pakistan Journal of Medical Sciences. 2018. 34:621

Chen HS, Wu T-EJ, -S Jap T, -A Lu R, -L Wang M, et al. Subclinical hypothyroidism is a risk factor for nephropathy and cardiovascular diseases in Type 2 diabetic patients. Diabetic Medicine. 2007. 24:1336

Cohen BM, B. R. Sommer, A. Vuckovic. Antidepressant-resistant depression in patients with comorbid subclinical hypothyroidism or high-normal TSH levels. American Journal of Psychiatry. 2018. 175:598

Cooper DS, Biondi B. Subclinical thyroid disease. The Lancet. 2012. 379:1142

Daniel Morell-Garcia, Josep Miquel Bauca, Miguel Angel Elorza, Antonia Barcelo. Two-step thyroid screening strategy in the critical patient. Clinical biochemistry. 2016. 49:925

Dardano A, Caraccio N, Monzani F. Evaluation of endothelial function in subclinical thyroid dysfunction [2]. Thyroid. 2006. 16:200

Dave JA, Klisiewicz A, Bayat Z, Mohamed NA, Stevens Z, et al. SEMDSA/ACE-SA guideline for the management of hypothyroidism in adults. Journal of Endocrinology, Metabolism and Diabetes of South Africa. 2015. 20:18

Dave JA, Klisiewicz A, Bayat Z, Mohamed NA, Stevens Z, et al. SEMDSA/ACE-SA guideline for the management of hypothyroidism in adults. SA Pharmaceutical Journal. 2016. 83:34

Delitala AP, Fanciulli G, Maioli M, Delitala G. Subclinical hypothyroidism, lipid metabolism and cardiovascular disease. European journal of internal medicine. 2017. 38:17

Delitala AP, Filigheddu F, Orru M, AlGhatrif M, Steri M, et al. No evidence of association between subclinical thyroid disorders and common carotid intima medial thickness or atherosclerotic plaque.Nutrition Metabolism & Cardiovascular Diseases. 2015. 25:1104

Delshad H, Mehran L, Tohidi M, Assadi M, Azizi F. The incidence of thyroid function abnormalities and natural course of subclinical thyroid disorders, Tehran, I.R. Iran. Journal of endocrinological investigation. 2012. 35:516

den Elzen WendyPJ, Lefebre-van de Fliert AnneA, Vanessa Virgini, Mooijaart SimonP, Peter Frey, et al. International variation in GP treatment strategies for subclinical hypothyroidism in older adults: a case-based survey. British Journal of General Practice. 2015. 65:e121

Dev N, Sahoo R, Kulshreshtha B, Gadpayle AK, Sharma SC. Prevalence of thyroid dysfunction and its correlation with CD4 count in newly-diagnosed HIV-positive adults – a cross-sectional study. International Journal of STD and AIDS. 2015. 26:965

Di Conza P, Affuso F, Fazio S. Thyroid Hormones and Cardiovascular System: From Bench to Bedside. Current Drug Therapy. 2010. 5:36

Diaz-Olmosi R, Nogueirai A, Penalva DQF, Lotufo PA, Benseñor IM. Frequency of subclinical thyroid dysfunction and risk factors for cardiovascular disease among women at a workplace. Sao Paulo Medical Journal. 2010. 128:18

Dietrich EA, K. Davis. Treating subclinical hypothyroidism with levothyroxine: Is there evidence of benefit?. Consultant. 2017. 57:#pages#

Díez JJ, Iglesias P, Burman KD. Spontaneous normalization of thyrotropin concentrations in patients with subclinical hypothyroidism. Journal of Clinical Endocrinology and Metabolism. 2005. 90:4124

Díez JJ, Iglesias P. Spontaneous subclinical hypothyroidism in patients older than 55 years: An analysis of natural course and risk factors for the development of overt thyroid failure. Journal of Clinical Endocrinology and Metabolism. 2004. 89:4890

Duntas LH, Biondi B. New insights into subclinical hypothyroidism and cardiovascular risk. Seminars in thrombosis and hemostasis. 2011. 37:27

Fabiana Pani, Francesco Atzori, Germana Baghino, Francesco Boi, Luciana Tanca, et al. Thyroid Dysfunction in Patients with Metastatic Carcinoma Treated with Sunitinib: Is Thyroid Autoimmunity Involved?. Thyroid. 2015. 25:1255

Fatourechi V, Lankarani M, Schryver PG, Vanness DJ, Long KH, et al. Factors influencing clinical decisions to initiate thyroxine therapy for patients with mildly increased serum thyrotropin (5.1-10.0 mIU/L). Mayo Clinic proceedings. 2003. 78:554

Ferreira MM, P. D. F. D. S. Teixeira, V. A. R. Mansur, V. S. Reuters, C. P. Almeida, M. Vaisman. Ambulatory blood pressure monitoring in normotensive patients with subclinical hypothyroidism. Arquivos Brasileiros de Cardiologia. 2010. 94:806

Fonseca RHA, P. F. S. Teixeira, M. M. Ferreira, M. M. T. Duarte, V. S. Reuters, C. P. Almeida, M. Vaisman. Echocardiographic evaluation of left and right ventricular function in subclinical hypothyroidism. Experimental and Clinical Endocrinology and Diabetes. 2009. 117:324

Fontes R, Patricia de Fatima Dos Santos Teixeira, Mario Vaisman. Screening of Undiagnosed Hypothyroidism in Elderly Persons with Diabetes according to Age-Specific Reference Intervals for Serum Thyroid Stimulating Hormone and the Impact of Antidiabetes Drugs.. Journal of Diabetes Research. 2016. 2016:1417408

Franklyn JA. The Thyroid - Too much and too little across the ages. the consequences of subclinical thyroid dysfunction. Clinical endocrinology. 2013. 78:1

Furukawa S, Yamamoto S, Todo Y, Maruyama K, Miyake T, et al. Association between subclinical hypothyroidism and diabetic nephropathy in patients with type 2 diabetes mellitus. Endocrine journal. 2014. 61:1011

Gabriela Brenta, Gabriela Berg, Veronica Miksztowicz, Graciela Lopez, Diego Lucero, et al. Atherogenic Lipoproteins in Subclinical Hypothyroidism and Their Relationship with Hepatic Lipase Activity: Response to Replacement Treatment with Levothyroxine. Thyroid. 2016. 26:365

Garber JR, Cobin RH, Gharib H, Hennessey JV, Klein I, et al. Clinical practice guidelines for hypothyroidism in adults: Cosponsored by the American Association of clinical endocrinologists and the American Thyroid Association. Thyroid. 2012. 22:1200

Gencer B, T. -H Collet, V. Virgini, D. C. Bauer, J. Gussekloo, A. R. Cappola, D. Nanchen, W. P. J. Den Elzen, P. Balmer, R. N. Luben, M. Iacoviello, V. Triggiani, J. Cornuz, A. B. Newman, K. -T Khaw, J. W. Jukema, R. G. J. Westendorp, E. Vittinghoff, D. Aujesky, N. Rodondi. Subclinical thyroid dysfunction and the risk of heart failure events an individual participant data analysis from 6 prospective cohorts. Circulation. 2012. 126:1040

Gunduz M, E. Gunduz, F. Kircelli, N. Okur, M. Ozkaya. Role of surrogate markers of atherosclerosis in clinical and subclinical thyroidism. International Journal of Endocrinology. 2012. 2012:#pages#

Gupta HR, Sheth SP, Vaishnav BS. Association of subclinical hypothyroidism with metabolic syndrome: A cross-sectional study from Western India. Asian Journal of Pharmaceutical and Clinical Research. 2016. 9:265

Gurgel MHC, R. M. Montenegro Junior, C. M. Melo Ponte, T. C. S. Sousa, P. G. B. Silva, L. De Sousa Belém, F. L. B. Furtado, L. A. De Araújo Batista, A. C. Pereira, R. D. Santos. Metabolic syndrome, diabetes and inadequate lifestyle in first-degree relatives of acute myocardial infarction survivors younger than 45 years old. Lipids in Health and Disease. 2017. 16:#pages#

Gussekloo J, Van Exel E, De Craen AJM, Meinders AE, Frölich M, et al. Thyroid status, disability and cognitive function, and survival in old age. Journal of the American Medical Association. 2004. 292:2591

Haddow JE. Preventing, identifying and managing thyroid deficiency in prenatal practice. Expert Review of Obstetrics and Gynecology. 2013. 8:213

Hassan A. Subclinical hypothyroidism. Thyroid Disorders: Basic Science and Clinical Practice. 2016. #volume#:203

Hauser AC, Gessl A, Lorenz M, Voigtländer T, Födinger M, et al. High prevalence of subclinical hypothyroidism in patients with Anderson-Fabry disease. Journal of inherited metabolic disease. 2005. 28:715

Helfand M. Screening for Subclinical Thyroid Dysfunction in Nonpregnant Adults: A Summary of the Evidence for the U.S. Preventive Services Task Force. Annals of Internal Medicine. 2004. 140:128

Hennessey JamesV, Garber JeffreyR, Woeber KennethA, Rhoda Cobin, Irwin Klein, et al. AMERICAN ASSOCIATION OF CLINICAL ENDOCRINOLOGISTS AND AMERICAN COLLEGE OF ENDOCRINOLOGY POSITION STATEMENT ON THYROID DYSFUNCTION CASE FINDING.. Endocrine Practice. 2016. 22:262

Hennessey JV, Espaillat R. Diagnosis and Management of Subclinical Hypothyroidism in Elderly Adults: A Review of the Literature. Journal of the American Geriatrics Society. 2015. 63:1663

Hennessey JV, Espaillat R. Reversible morbidity markers in subclinical hypothyroidism. Postgraduate medicine. 2015. 127:78

Hypothyroidism in adults: Levothyroxine if warranted by clinical and laboratory findings, not for simple TSH elevation. Prescrire international. 2015. 24:241

Iacoviello M, Guida P, Guastamacchia E, Triggiani V, Forleo C, et al. Prognostic role of sub-clinical hypothyroidism in chronic heart failure outpatients. Current pharmaceutical design. 2008. 14:2686

Ineck BA, Ng TMH. Effects of subclinical hypothyroidism and its treatment on serum lipids. Annals of Pharmacotherapy. 2003. 37:725

Ittermann T, S. Gläser, R. Ewert, S. Felix, H. Völzke, M. Dörr. Serum thyroid-stimulating hormone levels are not associated with exercise capacity and lung function parameters in two population-based studies. BMC Pulmonary Medicine. 2014. 14:#pages#

Jabbar A, Pingitore A, Pearce SHS, Zaman A, Iervasi G, et al. Thyroid hormones and cardiovascular disease. Nature Reviews Cardiology. 2016. 14:39

Jaiswal P, Y. Saxena, R. Gupta, R. M. Kaushik. Assessment of heart rate recovery and chronotropic incompetence in subclinical hypothyroid adults. Indian journal of physiology and pharmacology. 2018. 62:132

Jaseem T, A. Hegde, M. Chakrapani, S. Rao, P. Manjrekar, M. S. Rukmini. Lipids and ischemia-modified albumin in mild subclinical hypothyroidism: Response to levothyroxine replacement. Asian Journal of Pharmaceutical and Clinical Research. 2017. 10:336

Javed Z, Sathyapalan T. Levothyroxine treatment of mild subclinical hypothyroidism: a review of potential risks and benefits. Therapeutic Advances in Endocrinology and Metabolism. 2016. 7:12

Jia F, Tian J, Deng F, Yang G, Long M, et al. Subclinical hypothyroidism and the associations with macrovascular complications and chronic kidney disease in patients with Type 2 diabetes. Diabetic Medicine. 2015. 32:1097

Jonklaas J, Bianco AC, Bauer AJ, Burman KD, Cappola AR, et al. Guidelines for the treatment of hypothyroidism: Prepared by the American thyroid association task force on thyroid hormone replacement. Thyroid. 2014. 24:1670

Jorde R, Joakimsen O, Stensland E, Mathiesen EB. Lack of significant association between intima-media thickness in the carotid artery and serum TSH level. The Tromsø Study. Thyroid. 2008. 18:21

Kaminski G, Michalkiewicz D, Makowski K, Podgajny Z, Szalus N, et al. Prospective echocardiographic evaluation of patients with endogenous subclinical hyperthyroidism and after restoring euthyroidism. Clinical endocrinology. 2011. 74:501

Kang EW, J. Y. Nam, T. -H Yoo, S. K. Shin, S. -W Kang, D. -S Han, S. H. Han. Clinical implications of subclinical hypothyroidism in continuous ambulatory peritoneal dialysis patients. American Journal of Nephrology. 2008. 28:908

Kapoor K, Saha A, Dubey NK, Goyal P, Suresh CP, et al. Subclinical non-autoimmune hypothyroidism in children with steroid resistant nephrotic syndrome. Clinical and Experimental Nephrology. 2014. 18:113

Karabag T, S. M. Dogan, T. Bayraktaroglu, M. R. Sayin, C. Buyukuysal, I. Akpinar, M. Aydin. Assessment of left atrial mechanical functions in thyroid dysfunction. Polskie archiwum medycyny wewnetrznej. 2013. 123:596

Karabulut A, A. Doğan, A. K. Tuzcu. Myocardial performance index for patients with overt and subclinical hypothyroidism. Medical Science Monitor. 2017. 23:2519

Karimi F, Kalantarhormozi MR, Dabbaghmanesh MH, Omrani GR. Thyroid disorders and the prevalence of antithyroid antibodies in shiraz population. Archives of Iranian Medicine. 2014. 17:347

Kasagi K, Takahashi N, Inoue G, Honda T, Kawachi Y, et al. Thyroid function in japanese adults as assessed by a general health checkup system in relation with thyroid-related antibodies and other clinical parameters. Thyroid. 2009. 19:937

Khan MA, T. Ahsan, U. L. Rehman, R. Jabeen, S. Farouq. Subclinical Hypothyroidism: Frequency, clinical presentations and treatment indications. Pakistan Journal of Medical Sciences. 2017. 33:818

Khan SH, Nadeem Fazal, Aamir Ijaz, Syed Mohsin Manzoor, Naveed Asif, Tariq Rafi, Muhammad Yasir, Najmusaquib Khan Niazi. Insulin Resistance and Glucose Levels in Subjects with Subclinical Hypothyroidism.. Jcpsp, Journal of the College of Physicians & Surgeons - Pakistan. 2017. 27:329

Kiliçaslan B, M. K. Tigen, A. S. Tekin, H. Çiftçi. Cardiac changes with subclinical hypothyroidism in obese women. Turk Kardiyoloji Dernegi Arsivi. 2013. 41:471

Kim BJ, S. H. Lee, S. J. Bae, H. K. Kim, J. W. Choe, H. Y. Kim, J. -M Koh, G. S. Kim. The association between serum thyrotropin (TSH) levels and bone mineral density in healthy euthyroid men. Clinical endocrinology. 2010. 73:396

Knudsen N, Laurberg P, Rasmussen LB, Bülow I, Perrild H, et al. Small differences in thyroid function may be important for body mass index and the occurrence of obesity in the population. Journal of Clinical Endocrinology and Metabolism. 2005. 90:4019

Koren Peleg R, Efrati S, Benbassat C, Fygenzo M, Golik A. The effect of levothyroxine on arterial stiffness and lipid profile in patients with subclinical hypothyroidism. Thyroid. 2008. 18:825

Kosar F, I. Sahin, N. Turan, E. Topal, Y. Aksoy, C. Taskapan. Evaluation of right and left ventricular function using pulsed-wave tissue Doppler echocardiography in patients with subclinical hypothyroidism. Journal of endocrinological investigation. 2005. 28:704

Kowalska I, Borawski J, Nikołajuk A, Budlewski T, Otziomek E, et al. Insulin sensitivity, plasma adiponectin and sICAM-1 concentrations in patients with subclinical hypothyroidism: Response to levothyroxine therapy. Endocrine. 2011. 40:95

Lago-Sampedro AM, Gutiérrez-Repiso C, Valdés S, Maldonado C, Colomo N, et al. Changes in thyroid function with age: Results from the Pizarra population-based longitudinal study. International journal of clinical practice. 2015. 69:577

Lai CC, S. -H Tang, D. Pei, C. -Y Wang, Y. -L Chen, C. -Z Wu, F. -C Hsiao, H. -S Chen, J. -Y Wang. The prevalence of subclinical thyroid dysfunction and its association with metabolic syndrome in Taiwanese elderly. International Journal of Gerontology. 2011. 5:25

Lazarus JH. Aspects of treatment of subclinical hypothyroidism. Thyroid. 2007. 17:313

Lee YK, Kim JE, Oh HJ, Park KS, Kim SK, et al. Serum TSH level in healthy koreans and the association of TSH with serum lipid concentration and metabolic syndrome. Korean Journal of Internal Medicine. 2011. 26:432

LeFevre ML, Siu AL, Bibbins-Domingo K, Baumann LC, Karina WD, et al. Screening for thyroid dysfunction: U.S. Preventive Services Task Force recommendation statement. Annals of Internal Medicine. 2015. 162:641

Li X,​ Wang Y,​ Guan Q,​ Zhao J,​ Gao L. The lipid-lowering effect of levothyroxine in patients with subclinical hypothyroidism: A systematic review and meta-analysis of randomized controlled trials. Clinical endocrinology. 2017.

Lin HJ, T. -D Wang. Nocturnal non-dipping: An overlooked clinical manifestation of subclinical hypothyroidism linking to increased cardiovascular risk. Acta Cardiologica Sinica. 2017. 33:495

Lingad-Sayas RC, C. N. Montano, M. J. C. Isidro. Prevalence of elevated TSH and its association with dyslipidemia and NAFLD among Filipino adult executive check-up patients in a Tertiary hospital. Phillippine Journal of Internal Medicine. 2017. 55:#pages#

Lioudaki E, Mavroeidi NG, Mikhailidis DP, Ganotakis ES. Subclinical hypothyroidism and vascular risk: An update. Hormones. 2013. 12:495

Liu C, Scherbaum WA, Schott M, Schinner S. Subclinical hypothyroidism and the prevalence of the metabolic syndrome. Hormone and Metabolic Research. 2011. 43:417

Liu FH, J. -S Hwang, C. -F Kuo, Y. -S Ko, S. -T Chen, J. -D Lin. Subclinical hypothyroidism and metabolic risk factors association: A health examination-based study in northern Taiwan. Biomedical Journal. 2018. 41:52

Lu M, -B Yang C, Gao L, -J Zhao J. Mechanism of subclinical hypothyroidism accelerating endothelial dysfunction (Review). Experimental and Therapeutic Medicine. 2015. 9:3

Luboshitzky R, A. Aviv, P. Herer, L. Lavie. Risk factors for cardiovascular disease in women with subclinical hypothyroidism. Thyroid. 2002. 12:421

Mahajan RD, Singh R. Thyroid dysfunction and total cholesterol - experience in a tertiary care hospital. Research Journal of Pharmaceutical, Biological and Chemical Sciences. 2011. 2:268

Mammen JS, McGready J, Oxman R, Chia CW, Ladenson PW, et al. Thyroid Hormone Therapy and Risk of Thyrotoxicosis in Community-Resident Older Adults: Findings from the Baltimore Longitudinal Study of Aging. Thyroid. 2015. 25:979

Mañas-Martinez AB, Gimeno-Orna JA. Age-based TSH levels in the hypothyroidism treatment. Hypothyroidism: Diagnosis and Screening, Genetic Influences and Treatment Options. 2013. #volume#:35

Mansourian AR. A review on cardiovascular diseases originated from subclinical hypothyroidism. Pakistan Journal of Biological Sciences. 2012. 15:58

Marchiori RC, Pereira LAF, Naujorks AA, Rovaris DL, Meinerz DF, et al. Improvement of blood inflammatory marker levels in patients with hypothyroidism under levothyroxine treatment. BMC Endocrine Disorders. 2015. 15:#pages#

Marrakchi S, Kanoun F, Idriss S, Kammoun I, Kachboura S. Arrhythmia and thyroid dysfunction. Herz. 2015. 40:101

Masaki M, K. Komamura, A. Goda, S. Hirotani, M. Otsuka, A. Nakabo, M. Fukui, S. Fujiwara, M. Sugahara, M. Lee-Kawabata, T. Tsujino, M. Koshiba, T. Masuyama. Elevated arterial stiffness and diastolic dysfunction in subclinical hypothyroidism. Circulation Journal. 2014. 78:1494

Mayor S. Levothyroxine shows no benefit in elderly patients with subclinical hypothyroidism, trial finds. BMJ (Online). 2017. 357:#pages#

Meena CL, R. D. Meena, R. Nawal, V. K. Meena, A. Bharti, L. P. Meena. Assessment of left ventricular diastolic dysfunction in sub-clinical hypothyroidism. Acta Informatica Medica. 2012. 20:218

MehmetçIk G, Becer E, Akbey A. Serum total antioxidant status, lipid profile, malondialdehyde and erythrocyte superoxide dismutase levels in Hashimoto thyroiditis patients treated with levothyroxine. Turkiye Klinikleri Journal of Medical Sciences. 2012. 32:1241

Meuwese CL, Gussekloo J, De Craen AJM, Dekker FW, Den Elzen WPJ. Thyroid status and renal function in older persons in the general population. Journal of Clinical Endocrinology and Metabolism. 2014. 99:2689

Meyerovitch J, Rotman-Pikielny P, Sherf M, Battat E, Levy Y, et al. Serum thyrotropin measurements in the community: Five-year follow-up in a large network of primary care physicians. Archives of Internal Medicine. 2007. 167:1533

Meziou SM, Kanoun F, Marrakchi DI, Kammoun I, Kachboura S. Thyroid dysfunction and arrhythmias. Thyroid Disorders: Basic Science and Clinical Practice. 2016. #volume#:273

Mijin Kim, Tae Yong Kim, Soo Han Kim, Yunkyoung Lee, Su-yeon Park, et al. Reference interval for thyrotropin in a ultrasonography screened Korean population.. Korean Journal of Internal Medicine. 2015. 30:335

Miller M. Subclinical thyroid disorders. Clinical Geriatrics. 2005. 13:38

Minarikova Z, Gaspar L, Kruzliak P, Celecová Z, Oravec S. The effects of treatment on lipoprotein subfractions evaluated by polyacrylamide gel electrophoresis in patients with autoimmune hypothyroidism and hyperthyroidism. Lipids in Health and Disease. 2014. 13:#pages#

Mulić M, Š. Hajrović, R. Prelević, B. Milev. The frequency of metabolic syndrome in patients with the subclinical hypothyroidism. Vojnosanitetski Pregled. 2018. 75:398

Nakayama Y, M. Ohno, S. Yonemura, H. Uozumi, N. Kobayakawa, K. Fukushima, H. Takeuchi, T. Aoyagi. A case of transient 2:1 atrioventricular block, resolved by thyroxine supplementation for subclinical hypothyroidism. PACE - Pacing and Clinical Electrophysiology. 2006. 29:106

Neves C, Alves M, Medina JL, Delgado JL. Thyroid diseases, dyslipidemia and cardiovascular pathology. Revista Portuguesa de Cardiologia. 2008. 27:1211

Ng YY, S. C. Wu, H. D. Lin, F. H. Hu, C. C. Hou, Y. Y. Chou, S. M. Chiu, Y. H. Sun, S. S. -Y Cho, W. C. Yang. Prevalence of clinical and subclinical thyroid disease in a peritoneal dialysis population. Peritoneal Dialysis International. 2012. 32:86

Ngumezi C, Archambault M. When does treatment of subclinical hypothyroidism reduce cardiovascular risk?. Journal of the American Academy of Physician Assistants. 2012. 25:57

Nixon M, R. G. J. Westendorp. When subclinical hypothyroidism becomes clinically diagnosed. European journal of internal medicine. 2017. 46:e34

Novac ER, Roşu N, Cotârleţ A. Effects of levothyroxine treatment on the quality of life of women diagnosed with subclinical hypothyroidism. Archives of the Balkan Medical Union. 2015. 50:186

Nygaard B . Hypothyroidism (primary). Clinical Evidence. 2010.

Oflaz H, R. Kurt, A. Cimen, A. Elitok, I. Onur, E. Golcuk, M. Demirturk, S. Batmaz, E. Kasikcioglu. Coronary flow reserve is also impaired in patients with subclinical hypothyroidism. International journal of cardiology. 2007. 120:414

Ogunsakin A, S. S. Solomon, S. M. D. Dagogo-Jack. Thyroid hormone therapy for older adults with subclinical hypothyroidism. New England Journal of Medicine. 2017. 377:e20.1

Olsen AH, Kelsberg G, Coffey J, Hsu JT. Should we screen women for hypothyroidism?. Journal of Family Practice. 2004. 53:653

OMayerJr, Šimon J, Filipovský J, Pláškova M, Pikner R. Hypothyroidism in coronary heart disease and its relation to selected risk factors. Vascular Health and Risk Management. 2006. 2:499

Öner FA, S. Yurdakul, E. Öner, M. K. Arslantas, M. Usta, M. Ergüney. Evaluation of ventricular functions using tissue Doppler echocardiography in patients with subclinical hypothyroidism. Turk Kardiyoloji Dernegi Arsivi. 2011. 39:129

Palmeiro C, Davila MI, Bhat M, Frishman WH, Weiss IA. Subclinical hyperthyroidism and cardiovascular risk: Recommendations for treatment. Cardiology in review. 2013. 21:300

Pandrc MS, A. Ristic, V. Kostovski, M. Stankovic, V. Antic, J. Milin-Lazovic, J. Ziric. The Effect of Early Substitution of Subclinical Hypothyroidism on Biochemical Blood Parameters and the Quality of Life. Journal of Medical Biochemistry. 2017. 36:127

Parent S, B. Cujec. Subclinical Hypothyroidism and Heart Failure: Chicken or Egg?. Canadian Journal of Cardiology. 2018. 34:11

Parretti H, Onyebuchi Okosieme, Mark Vanderpump. Current recommendations in the management of hypothyroidism: developed from a statement by the British Thyroid Association Executive.. British Journal of General Practice. 2016. 66:538

Pasqualetti G, Tognini S, Polini A, Caraccio N, Monzani F. Is subclinical hypothyroidism a cardiovascular risk factor in the elderly?. Journal of Clinical Endocrinology and Metabolism. 2013. 98:2256

Pasqualetti G, Tognini S, Polini A, Caraccio N, Monzani F. Subclinical hypothyroidism and heart failure risk in older people. Endocrine, Metabolic and Immune Disorders - Drug Targets. 2013. 13:13

Patanè S. Cardiovascular system and endogenous subclinical hyperthyroidism treatment: The time has come. International journal of cardiology. 2012. 158:317

Pearce EN, Q. Yang, E. J. Benjamin, J. Aragam, R. S. Vasan. Thyroid function and left ventricular structure and function in the framingham heart study. Thyroid. 2010. 20:369

Peixoto de Miranda EJF, M. S. Bittencourt, A. C. Pereira, A. C. Goulart, I. S. Santos, P. A. Lotufo, I. M. Bensenor. Subclinical hypothyroidism is associated with higher carotid intima-media thickness in cross-sectional analysis of the Brazilian Longitudinal Study of Adult Health (ELSA-Brasil).. Nutrition Metabolism & Cardiovascular Diseases. 2016. 26:915

Pérez A, Cubero JM, Sucunza N, Ortega E, Arcelús R, et al. Emerging cardiovascular risk factors in subclinical hypothyroidism: Lack of change after restoration of euthyroidism. Metabolism: Clinical and Experimental. 2004. 53:1512

Qavi A, G. N. Khokhar, A. Zahoor, I. A. Akhund, M. Ishaq. Significance of hypercholesterolemia in hypothyroid patients. Medical Forum Monthly. 2013. 24:86

Qi Q, Q. -M Zhang, C. -J Li, R. -N Dong, J. -J Li, J. -Y Shi, D. -M Yu, J. -Y Zhang. Association of thyroid-stimulating hormone levels with microvascular complications in type 2 diabetes patients. Medical Science Monitor. 2017. 23:2715

Qian Wu, Rayman MargaretP, Hongjun Lv, Lutz Schomburg, Bo Cui, et al. Low Population Selenium Status Is Associated With Increased Prevalence of Thyroid Disease.. Journal of Clinical Endocrinology & Metabolism. 2015. 100:4037

Quan X, Y. Ji, C. Zhang, X. Guo, Y. Zhang, S. Jia, W. Ma, Y. Fan, C. Wang. Circulating MiR-146a May be a Potential Biomarker of Coronary Heart Disease in Patients with Subclinical Hypothyroidism. Cellular Physiology and Biochemistry. 2018. 45:226

Racataianu N, Nicoleta Leach, Cosmina Ioana Bondor, Smaranda Marza, Daniela Moga, Ana Valea, Cristina Ghervan. Thyroid disorders in obese patients. Does insulin resistance make a difference?.. Archives of Endocrinology & Metabolism. 2017. 61:575

Rana P, G. Sripathy, A. Varshney, P. Kumar, M. Memita Devi, R. K. Marwaha, R. P. Tripathi, S. Khushu. Phosphorous magnetic resonance spectroscopy-based skeletal muscle bioenergetic studies in subclinical hypothyroidism. Journal of endocrinological investigation. 2012. 35:129

Razvi S, Weaver JU, Pearce SHS. Subclinical thyroid disorders: Significance and clinical impact. Journal of clinical pathology. 2010. 63:379

Reuters VS, P. D. F. S. Teixeira, P. S. Vigário, C. P. Almeida, A. Buescu, M. M. Ferreira, C. L. N. De Castro, J. Gold, M. Vaisman. Functional capacity and muscular abnormalities in subclinical hypothyroidism. American Journal of the Medical Sciences. 2009. 338:259

Rhee CM, Amy S. You, Danh V. Nguyen, Steven M. Brunelli, Matthew J. Budoff, Elani Streja, Tracy Nakata, Csaba P. Kovesdy, Gregory A. Brent, Kamyar Kalantar-Zadeh. Thyroid Status and Mortality in a Prospective Hemodialysis Cohort.. Journal of Clinical Endocrinology & Metabolism. 2017. 102:1568

Rhee CM, Vanessa A. Ravel, Elani Streja, Rajnish Mehrotra, Steven Kim, Jiaxi Wang, Danh V. Nguyen, Csaba P. Kovesdy, Gregory A. Brent, Kamyar Kalantar-Zadeh. Thyroid Functional Disease and Mortality in a National Peritoneal Dialysis Cohort.. Journal of Clinical Endocrinology & Metabolism. 2016. 101:4054

Risal P, Maharjan BR, Koju R, Makaju RK, Gautam M. Variation of total serum cholesterol among the patient with thyroid dysfunction. Kathmandu University Medical Journal. 2010. 8:265

Roberts LM, Pattison H, Roalfe A, Franklyn J, Wilson S, et al. Is subclinical thyroid dysfunction in the elderly associated with depression or cognitive dysfunction?. Annals of Internal Medicine. 2006. 145:573

Robles-Osorio MaL, Zacarías-Rangel V, García-Solís P, Hernández-Montiel HL, Solís JC, et al. Prevalence of thyroid function test abnormalities and anti-thyroid antibodies an open population in central México. Revista de Investigacion Clinica. 2014. 66:113

Rosario PW, Calsolari MR. TSH reference range in older adults: A Brazilian study. Arquivos Brasileiros de Endocrinologia e Metabologia. 2014. 58:389

Ross DS. Subclinical hypothyroidism: When to treat, when to watch. Consultant. 2011. 51:769

Rugge JB,​ Bougatsos C,​ Chou R. Screening and treatment of thyroid dysfunction: An evidence review for the U.S. preventive services task force. Annals of Internal Medicine. 2015. 162:35

Saigal P, Sharma U. Does levothyroxine supplementation reduce subjective symptoms in patients with subclinical hypothyroidism?. Evidence-Based Practice. 2012. 15:9

Samuels MaryH, Irina Kolobova, Anne Smeraglio, Dawn Peters, Purnell JonathanQ, et al. Effects of Levothyroxine Replacement or Suppressive Therapy on Energy Expenditure and Body Composition.. Thyroid. 2016. 26:347

Samuels MH. Subclinical Hypothyroidism and Depression: Is There a Link?. Journal of Clinical Endocrinology and Metabolism. 2018. 103:2061

Schalin-Jäntti C, Ojala AK, Pitkälä KH, Tilvis RS, Strandberg TE. Thyroid-stimulating hormone and mortality in older people. Journal of the American Geriatrics Society. 2013. 61:1823

Sgarbi JA, Matsumura LK, Kasamatsu TS, Ferreira SR, Maciel RMB. Subclinical thyroid dysfunctions are independent risk factors for mortality in a 7.5-year follow-up: The Japanese-Brazilian thyroid study. European Journal of Endocrinology. 2010. 162:569

Sgarbi JA, Teixeira PFS, Maciel LMZ, Mazeto GMFS, Vaisman M, et al. The Brazilian consensus for the clinical approach and treatment of subclinical hypothyroidism in adults: Recommendations of the thyroid department of the Brazilian Society of Endocrinology and Metabolism. Arquivos Brasileiros de Endocrinologia e Metabologia. 2013. 57:166

Shah R. In older adults with subclinical hypothyroidism, levothyroxine did not improve symptoms or tiredness. Annals of Internal Medicine. 2017. 167:JC14

Sharma AK, R. Arya, R. Mehta, R. Sharma, A. K. Sharma. Hypothyroidism and cardiovascular disease: Factors, mechanism and future perspectives. Current medicinal chemistry. 2013. 20:4411

Sharma N, Sharma LK, Dutta D, Gadpayle AK, Anand A, et al. Prevalence and predictors of thyroid dysfunction in patients with HIV infection and acquired immunodeficiency syndrome: An Indian perspective. Journal of Thyroid Research. 2015. 2015:#pages#

Shin DH, Lee MJ, Lee HS, Oh HJ, Ko KI, et al. Thyroid hormone replacement therapy attenuates the decline of renal function in chronic kidney disease patients with subclinical hypothyroidism. Thyroid. 2013. 23:654

Sikandar HK, S. M. Manzoor, N. K. Niazi, N. Asif, A. Ijaz, N. Fazal. Association of metabolic risks with subclinical hypothyroidism: A cross-sectional analysis. Pakistan Journal of Medical Sciences. 2018. 34:357

Solini A, F. Monzani. Hypothyroidism and intermediate metabolism: A complex relationship. Thyroid. 2010. 20:837

Stern RA, Hennessey JV. Screening for thyroid dysfunction. Annals of Internal Medicine. 2015. 163:564

Surks MI, Goswami G, Daniels GH. The thyrotropin reference range should remain unchanged. Journal of Clinical Endocrinology and Metabolism. 2005. 90:5489

Surks MI, Ortiz E, Daniels GH, Sawin CT, Col NF, et al. Subclinical Thyroid Disease: Scientific Review and Guidelines for Diagnosis and Management. Journal of the American Medical Association. 2004. 291:228

Svare A, Nilsen TIL, Bjøro T, Åsvold BO, Langhammer A. Serum TSH related to measures of body mass: Longitudinal data from the HUNT Study, Norway. Clinical endocrinology. 2011. 74:769

Tagami T, Kimura H, Ohtani S, Tanaka T, Tanaka T, et al. Multi-center study on the prevalence of hypothyroidism in patients with hypercholesterolemia. Endocrine journal. 2011. 58:449

Takashima N, Niwa Y, Mannami T, Tomoike H, Iwai N. Characterization of subclinical thyroid dysfunction from cardiovascular and metabolic viewpoints - The Suita study. Circulation Journal. 2007. 71:191

Taylor PN, Iqbal A, Minassian C, Sayers A, Draman MS, et al. Falling threshold for treatment of borderline elevated thyrotropin levels - Balancing benefits and risks evidence from a large community-based study. JAMA Internal Medicine. 2014. 174:32

Tng EL. The debate on treating subclinical hypothyroidism. Singapore medical journal. 2016. 57:539

Tognini S, Pasqualetti G, Calsolaro V, Polini A, Monzani F. Cognitive function and quality of life in mild thyroid hormone deficiency.. Recent Patents on Endocrine, Metabolic & Immune Drug Discovery. 2014. 8:124.

Tognini S, Polini A, Pasqualetti G, Ursino S, Caraccio N, et al. Age and gender substantially influence the relationship between thyroid status and the lipoprotein profile: Results from a large cross-sectional study. Thyroid. 2012. 22:1096

USPSTF. Screening for thyroid dysfunction: Recommendation statement. American Family Physician. 2015. 91:790A

Uzun MK, N. Taş, J. Meray, M. Çölbay, I. Yetkin. Evaluation of muscle strength and fatigue using isokinetic testing in hypothyroid patients. Turkiye Fiziksel Tip ve Rehabilitasyon Dergisi. 2013. 59:286

Uzunlulu M, E. Yorulmaz, A. Oguz. Prevalence of subclinical hypothyroidism in patients with metabolic syndrome. Endocrine journal. 2007. 54:71

Valencia WM, Florez H. Endocrinology. Geriatrics for Specialists. 2016. #volume#:269

Veltri F, Rocha FO, Willems D, -P Praet J, Grabczan L, et al. Prevalence of thyroid dysfunction and autoimmunity in the elderly older population and implications of age-specific reference ranges. Clinica Chimica Acta. 2017. 465:34

Verburg FrederikA, Inge Grelle, Kathrin Tatschner, Christoph Reiners, Markus Luster. Prevalence of thyroid disorders in elderly people in Germany. A screening study in a country with endemic goitre.. Nuclear-Medizin. 2017. 56:9

Vitale G, M. Galderisi, G. A. Lupoli, A. Celentano, I. Pietropaolo, N. Parenti, O. De Divitiis, G. Lupoli. Left ventricular myocardial impairment in subclinical hypothyroidism assessed by a new ultrasound tool: Pulsed tissue doppler. Journal of Clinical Endocrinology and Metabolism. 2002. 87:4350

Walsh JP, Bremner AP, Bulsara MK, O'Leary P, Leedman PJ, et al. Thyroid dysfunction and serum lipids: A community-based study. Clinical endocrinology. 2005. 63:670

Wang J, Ma X, Qu S, Li Y, Han L, et al. High prevalence of subclinical thyroid dysfunction and the relationship between thyrotropin levels and cardiovascular risk factors in residents of the coastal area of China. Experimental and Clinical Cardiology. 2013. 18:e16

Werneck FZ, E. F. Coelho, J. R. P. De Lima, M. C. Laterza, M. M. Barral, P. D. F. D. S. Teixeira, M. Vaisman. Pulmonary oxygen uptake kinetics during exercise in subclinical hypothyroidism. Thyroid. 2014. 24:931

Wiersinga WM. Should we treat mild subclinical/mild hyperthyroidism? Yes. European journal of internal medicine. 2011. 22:324

Wirth CD, Blum MR, da Costa BR, Baumgartner C, Collet TH, et al. Subclinical thyroid dysfunction and the risk for fractures: a systematic review and meta-analysis. Annals of Internal Medicine. 2014. 161:189.

Xu YY, J. Liang, Y. Cao, F. Shan, Y. Liu, Q. -R Xia. High levels of Nesfatin-I in relation to the dysfunction of the hypothalamic-pituitary-adrenal and hypothalamus-pituitary-thyroid axes in depressed patients with subclinical hypothyroidism. Neuropsychiatric Disease and Treatment. 2017. 13:1647

Yao Z, X. Gao, M. Liu, Z. Chen, N. Yang, Y. -M Jia, X. -M Feng, Y. Xu, X. -C Yang, G. Wang. Diffuse Myocardial Injuries are Present in Subclinical Hypothyroidism: A Clinical Study Using Myocardial T1-mapping Quantification. Scientific Reports. 2018. 8:#pages#

Yen-Ting Chen, Fen-Yu Tseng, Pei-Lung Chen, Yu-Chao Chi, Der-Sheng Han, et al. Serum Spot 14 concentration is negatively associated with thyroid-stimulating hormone level. Medicine. 2016. 95:e5036

Yilmaz H, M. Cakmak, T. Darcin, O. Inan, O. M. Gurel, M. A. Bilgic, N. Bavbek, A. Akcay. Subclinical hypothyroidism in combination with vitamin D deficiency increases the risk of impaired left ventricular diastolic function. Endocrine regulations. 2015. 49:84

Yong Hwang, Won Kim, So Young Kwon, Hyung Min Yu, Jeong Han Kim, et al. Incidence of and risk factors for thyroid dysfunction during peginterferon alpha and ribavirin treatment in patients with chronic hepatitis C.. Korean Journal of Internal Medicine. 2015. 30:792

Zhang X, Yi Xie, Caixia Ding, Jing Xiao, Yinyan Tang, Xuemei Jiang, Hua Shan, Yuankai Lin, Yujia Zhu, Chuanyou Li, Dan Hu, Zhixiang Ling, Gelin Xu, Lei Sheng. Subclinical hypothyroidism and risk of cerebral small vessel disease: A hospital-based observational study.. Clinical endocrinology. 2017. 87:581

Zhang Y, Chang Y, Ryu S, Cho J, -Y Lee W, et al. Thyroid hormones and mortality risk in euthyroid individuals: The Kangbuk Samsung health study. Journal of Clinical Endocrinology and Metabolism. 2014. 99:2467

Zhao JV, C. M. Schooling. Thyroid function and ischemic heart disease: A Mendelian randomization study. Scientific Reports. 2017. 7:#pages#

Zhaowei Meng, Ming Liu, Qing Zhang, Li Liu, Kun Song, et al. Gender and Age Impacts on the Association Between Thyroid Function and Metabolic Syndrome in Chinese. Medicine. 2015. 94:e2193

**Wrong intervention: n = 22**

Arinc H, H. Gunduz, A. Tamer, E. Seyfeli, M. Kanat, H. Ozhan, R. Akdemir, H. Celebi, C. Uyan. Evaluation of right ventricular function in patients with thyroid dysfunction. Cardiology. 2006. 105:89

Abdel del Busto-Mesa, Julio Oscar Cabrera-Rego, Lisvan Carrero-Fernandez, Cristina Victoria Hernandez-Roca, Jorge Luis Gonzalez-Valdes, et al. Changes in arterial stiffness, carotid intima-media thickness, and epicardial fat after L-thyroxine replacement therapy in hypothyroidism. *Endocrinologia y Nutricion.* 2015. 62:270

Amati F, Dubé JJ, Stefanovic-Racic M, Toledo FG, Goodpaster BH. Improvements in insulin sensitivity are blunted by subclinical hypothyroidism. *Medicine and science in sports and exercise.* 2009. 41:265

Andreas Nearchou, Antonis Valachis, Pehr Lind, Olof Akre, Per Sandstrom. Acquired Hypothyroidism as a Predictive Marker of Outcome in Patients With Metastatic Renal Cell Carcinoma Treated With Tyrosine Kinase Inhibitors: A Literature-Based Meta-Analysis. *Clinical Genitourinary Cancer.* 2015. 13:280

Cabral MD, Teixeira PFS, Silva NAO, Morais FFC, Soares DV, et al. Normal flow-mediated vasodilatation of the brachial artery and carotid artery intima-media thickness in subclinical hypothyroidism. *Brazilian Journal of Medical and Biological Research.* 2009. 42:426

Chao Ma, Jiawei Xie, Hui Wang, Jinsong Li, Suyun Chen. Radioiodine therapy versus antithyroid medications for Graves' disease. *#journal#.* 2016. #volume#:#pages#

Eskes SA, Endert E, Fliers E, Birnie E, Hollenbach B, et al. Selenite supplementation in euthyroid subjects with thyroid peroxidase antibodies. *Clinical endocrinology.* 2014. 80:444.

Farhangi MA, P. Dehghan, S. Tajmiri. Powdered black cumin seeds strongly improves serum lipids, atherogenic index of plasma and modulates anthropometric features in patients with Hashimoto's thyroiditis. Lipids in Health and Disease. 2018. 17:#pages#

Farhangi MA, P. Dehghan, S. Tajmiri, M. M. Abbasi. The effects of Nigella sativa on thyroid function, serum Vascular Endothelial Growth Factor (VEGF) - 1, Nesfatin-1 and anthropometric features in patients with Hashimoto's thyroiditis: A randomized controlled trial. BMC Complementary and Alternative Medicine. 2016. 16:#pages#

Gawlik A, Such K, Dejner A, Zachurzok A, Antosz A, et al. Subclinical hypothyroidism in children and adolescents: Is it clinically relevant?. *International Journal of Endocrinology.* 2015. 2015:#pages#

Giuseppe Pasqualetti, Gennaro Pagano, Giuseppe Rengo, Nicola Ferrara, Fabio Monzani. Subclinical Hypothyroidism and Cognitive Impairment: Systematic Review and Meta-Analysis. *Journal of Clinical Endocrinology & Metabolism.* 2015. 100:4240

Gluvic Z, Sudar E, Tica J, Jovanovic A, Zafirovic S, et al. Effects of levothyroxine replacement therapy on parameters of metabolic syndrome and atherosclerosis in hypothyroid patients: A prospective pilot study. *International Journal of Endocrinology.* 2015. 2015:#pages#

Isabelle Herter-Aeberli, Mohamed Cherkaoui, Nawal El Ansari, Riccarda Rohner, Sara Stinca, et al. Iodine Supplementation Decreases Hypercholesterolemia in Iodine-Deficient, Overweight Women: A Randomized Controlled Trial. *Journal of Nutrition.* 2015. 145:2067

Kaminski J, Miasaki FY, Paz-Filho G, Graf H, de Carvalho GA. Treatment of hypothyroidism with levothyroxine plus liothyronine: A randomized, double-blind, crossover study. *Archives of Endocrinology and Metabolism.* 2016. 60:562

Mitchell JE, Hellkamp AS, Mark DB, Anderson J, Johnson GW, et al. Thyroid function in heart failure and impact on mortality. *JACC: Heart Failure.* 2013. 1:48

Perez AC, Jhund PS, Stott DJ, Gullestad L, Cleland JG, et al. Thyroid-stimulating hormone and clinical outcomes: the CORONA trial (controlled rosuvastatin multinational study in heart failure). *JACC Heart Failure.* 2014. 2:35.

Rankin S, Douglas H. Elder, Simon Ogston, Jacob George, Chim C. Lang, Anna Maria Choy. Population-level incidence and monitoring of adverse drug reactions with long-term amiodarone therapy.. Cardiovascular therapeutics. 2017. 35:#pages#

Razvi S, Shakoor A, Vanderpump M, Weaver JU, Pearce SHS. The influence of age on the relationship between subclinical hypothyroidism and ischemic heart disease: A metaanalysis. *Journal of Clinical Endocrinology and Metabolism.* 2008. 93:2998

Sharma AK, Indraneel Basu, Siddarth Singh. Efficacy and Safety of Ashwagandha Root Extract in Subclinical Hypothyroid Patients: A Double-Blind, Randomized Placebo-Controlled Trial.. Journal of Alternative & Complementary Medicine. 2018. 24:243

Valentina VN, Marijan B, Chedo D, Branka K. Subclinical hypothyroidism and risk to carotid atherosclerosis. *Arquivos Brasileiros de Endocrinologia e Metabologia.* 2011. 55:475

Vestergaard P, Mosekilde L. Hyperthyroidism, bone mineral, fracture risk - A meta-analysis. *Thyroid.* 2003. 13:585

Watt T, Groenvold M, Rasmussen ÅK, Bonnema SJ, Hegedüs L, et al. Quality of life in patients with benign thyroid disorders. A review. *European Journal of Endocrinology.* 2006. 154:501

**Wrong comparator group: n = 105**

Adamarczuk-Janczyszyn M, Zdrojowy-Welna A, Rogala N, Zatońska K, Bednarek-Tupikowska G. Evaluation of selected atherosclerosis risk factors in women with subclinical hypothyroidism treated with L-Thyroxine. Advances in Clinical and Experimental Medicine. 2016. 25:457

Adrees M, Gibney J, El-Saeity N, Boran G. Effects of 18 months of l-T4 replacement in women with subclinical hypothyroidism. Clinical endocrinology. 2009. 71:298

Akbar DH, Ahmed MM, Hijazi NA. Subclinical hypothyroidism in elderly women attending an outpatient clinic. Medical Science Monitor. 2004. 10:CR229

Amann BL, Joaquim Radua, Christian Wunsch, Barbara Konig, Christian Simhandl. Psychiatric and physical comorbidities and their impact on the course of bipolar disorder: A prospective, naturalistic 4-year follow-up study.. Bipolar disorders. 2017. 19:225

Arinc H, H. Gunduz, A. Tamer, E. Seyfeli, M. Kanat, H. Ozhan, R. Akdemir, C. Uyan. Tissue Doppler echocardiography in evaluation of cardiac effects of subclinical hypothyroidism. International Journal of Cardiovascular Imaging. 2006. 22:177

Aubert CE, D. C. Bauer, B. R. da Costa, M. Feller, C. Rieben, E. M. Simonsick, K. Yaffe, N. Rodondi,the Health ABC Study. The association between subclinical thyroid dysfunction and dementia: The Health, Aging and Body Composition (Health ABC) Study. Clinical endocrinology. 2017. 87:617

Bilgir O, F. Bilgir, M. Calan, O. G. Calan, A. Yuksel. Comparison of pre- and post-levothyroxine highsensitivity c-reactive protein and fetuin-a levels in subclinical hypothyroidism. Clinics. 2015. 70:97

Birk-Urovitz E, M. Elisabeth Del Giudice, Christopher Meaney, Karan Grewal. Use of thyroid-stimulating hormone tests for identifying primary hypothyroidism in family medicine patients.. Canadian Family Physician. 2017. 63:e389

Blum MR, Bauer DC, -H Collet T, Fink HA, Cappola AR, et al. Subclinical thyroid dysfunction and fracture risk a meta-analysis. JAMA - Journal of the American Medical Association. 2015. 313:2055

Blum MR, Wijsman LW, Virgini VS, Bauer DC, Den Elzen WPJ, et al. Subclinical Thyroid Dysfunction and Depressive Symptoms among the Elderly: A Prospective Cohort Study. Neuroendocrinology. 2016. 103:291

Bo Abrahamsen, Jorgensen HenrikL, Anne Sofie Laulund, Mads Nybo, Bauer DougC, et al. The excess risk of major osteoporotic fractures in hypothyroidism is driven by cumulative hyperthyroid as opposed to hypothyroid time: an observational register-based time-resolved cohort analysis. Journal of Bone & Mineral Research. 2015. 30:898

Brenta G, Berg G, Arias P, Zago V, Schnitman M, et al. Lipoprotein alterations, hepatic lipase activity, and insulin sensitivity in subclinical hypothyroidism: Response to L-T4 treatment. Thyroid. 2007. 17:453

Brenta G, L. A. Mutti, M. Schnitman, O. Fretes, A. Perrone, M. L. Matute. Assessment of left ventricular diastolic function by radionuclide ventriculography at rest and exercise in subclinical hypothyroidism, and its response to L-thyroxine therapy. American Journal of Cardiology. 2003. 91:1327

Canturk Z, Çetinarslan B, Tarkun I, Canturk NZ, Özden M. Lipid profile and lipoprotein (a) as a risk factor for cardiovascular disease in women with subclinical hypothyroidism. Endocrine research. 2003. 29:307

Cerbone M, Capalbo D, Wasniewska M, Alfano S, Raso GM, et al. Effects of L-thyroxine treatment on early markers of atherosclerotic disease in children with subclinical hypothyroidism. European Journal of Endocrinology. 2016. 175:11

Chang CH, Y. -C Yeh, J. L. Caffrey, S. -R Shih, L. -M Chuang, Y. -K Tu. Metabolic syndrome is associated with an increased incidence of subclinical hypothyroidism - A Cohort Study. Scientific Reports. 2017. 7:#pages#

Curotto Grasiosi J, B. Peressotti, R. A. Machado, E. C. Filipini, A. Angel, J. Delgado, G. A. Cortez Quiroga, C. Rus Mansilla, M. D. M. Martínez Quesada, A. Degregorio, D. J. Cordero, M. Dak, C. Izurieta, R. J. Esper. Improvement in functional capacity after levothyroxine treatment in patients with chronic heart failure and subclinical hypothyroidism. Endocrinologia y Nutricion. 2013. 60:427

De Jongh RT, Lips P, Van Schoor NM, Rijs KJ, Deeg DJH, et al. Endogenous subclinical thyroid disorders, physical and cognitive function, depression, and mortality in older individuals. European Journal of Endocrinology. 2011. 165:545

Doin FC, M. Rosa-Borges, M. R. A. Martins, V. A. Moisés, J. Abucham. Diagnosis of subclinical central hypothyroidism in patients with hypothalamic-pituitary disease by Doppler echocardiography. European Journal of Endocrinology. 2012. 166:631

Emamifar A, Inger Marie Jensen Hansen. The influence of thyroid diseases, diabetes mellitus, primary hyperparathyroidism, vitamin B12 deficiency and other comorbid autoimmune diseases on treatment outcome in patients with rheumatoid arthritis: An exploratory cohort study.. Medicine. 2018. 97:e10865

Emamifar A, Jorgen Hangaard, Inger Marie Jensen Hansen. Thyroid disorders in patients with newly diagnosed rheumatoid arthritis is associated with poor initial treatment response evaluated by disease activity score in 28 joints-C-reactive protein (DAS28-CRP): An observational cohort study.. Medicine. 2017. 96:e8357

Erem C, Suleyman AK, Civan N, Mentese A, Nuhoglu İ, et al. The effect of L-thyroxine replacement therapy on ischemia-modified albümin and malondialdehyde levels in patients with overt and subclinical hypothyroidism. Endocrine research. 2016. 41:350

Erkan G, A. F. Erkan, M. Cemri, S. Karaahmetoglu, M. Cesur, A. Cengel. The evaluation of diastolic dysfunction with tissue Doppler echocardiography in women with subclinical hypothyroidism and the effect of L-thyroxine treatment on diastolic dysfunction: A pilot study. Journal of Thyroid Research. 2011. 2011:#pages#

Faber J, L. Petersen, N. Wiinberg, S. Schifter, J. Mehlsen. Hemodynamic changes after levothyroxine treatment in subclinical hypothyroidism. Thyroid. 2002. 12:319

Ferda Bilgir, Oktay Bilgir, Mehmet Calan, Ozlem Calan, Tolgay Isikyakar. Subclinical hypothyroidism: Comparison of adhesion molecule levels before and after levothyroxine therapy. Journal of International Medical Research. 2014. 42:806

Ferrari SM, P. Fallahi, F. Di Bari, R. Vita, S. Benvenga, A. Antonelli. Myo-inositol and selenium reduce the risk of developing overt hypothyroidism in patients with autoimmune thyroiditis.. European Review for Medical & Pharmacological Sciences. 2017. 21:36

Franzoni F, Galetta F, Fallahi P, Tocchini L, Merico G, et al. Effect of l-thyroxine treatment on left ventricular function in subclinical hypothyroidism. Biomedicine and Pharmacotherapy. 2006. 60:431

Frisullo G, Massimiliano Calabrese, Carla Tortorella, Damiano Paolicelli, Paolo Ragonese, Pietro Annovazzi, Marta Radaelli, Simona Malucchi, Antonio Gallo, Valentina Tomassini, Viviana Nociti, Mariangela D'Onghia, Vincenzina Lo Re, Mariemma Rodegher, Claudio Solaro, Claudio Gasperini. Thyroid autoimmunity and dysfunction in multiple sclerosis patients during long-term treatment with interferon beta or glatiramer acetate: an Italian multicenter study.. Multiple Sclerosis. 2014. 20:1265

Galetta F, Franzoni F, Fallahi P, Rossi M, Carpi A, et al. Heart rate variability and QT dispersion in patients with subclinical hypothyroidism. Biomedicine and Pharmacotherapy. 2006. 60:425

Gao N, Zhang W, -Z Zhang Y, Yang Q, -H Chen S. Carotid intima-media thickness in patients with subclinical hypothyroidism: A meta-analysis. Atherosclerosis. 2013. 227:18

Garces-Arteaga A, N. Nieto-Garcia, F. Suarez-Sanchez, H. R. Triana-Reina, R. Ramírez-Vélez. Influence of a medium-impact exercise program on health-related quality of life and cardiorespiratory fitness in females with subclinical hypothyroidism: An open-label pilot study. Journal of Thyroid Research. 2013. 2013:#pages#

Gencer B, -H Collet T, Virgini V, Auer R, Rodondi N. Subclinical thyroid dysfunction and cardiovascular outcomes among prospective cohort studies. Endocrine, Metabolic and Immune Disorders - Drug Targets. 2013. 13:4

Giri A, Edwards TL, LeGrys VA, Lorenz CE, Funk MJ, et al. Subclinical hypothyroidism and risk for incident ischemic stroke among postmenopausal women. Thyroid. 2014. 24:1210.

Godinjak A, Z. Velija-Asimi, A. Burekovic, A. Dizdarevic-Bostandzic, S. Jusufovic, A. Iglica, S. Gicic, A. Kukuljac. Subclinical hypothyroidism is associated with atherogenic lipid profile in postmenopausal women. Turkish Journal of Endocrinology and Metabolism. 2017. 21:37

Golledge J, G. J. Hankey, O. P. Almeida, L. Flicker, P. E. Norman, B. B. Yeap. Plasma free thyroxine in the upper quartile is associated with an increased incidence of major cardiovascular events in older men that do not have thyroid dysfunction according to conventional criteria. International journal of cardiology. 2018. 254:316

Gopinath B, Gerald Liew, Annette Kifley, Paul Mitchell. Thyroid Dysfunction and Ten-Year Incidence of Age-Related Macular Degeneration.. Investigative ophthalmology & visual science. 2016. 57:5273

Gulhan Akbaba, Dilek Berker, Serhat Isik, Mazhar Muslum Tuna, Suha Koparal, et al. Changes in the before and after thyroxine treatment levels of adipose tissue, leptin, and resistin in subclinical hypothyroid patients. Wiener klinische Wochenschrift. 2016. 128:579

Gullu S, Sav H, Kamel N. Effects of levothyroxine treatment on biochemical and hemostasis parameters in patients with hypothyroidism. European Journal of Endocrinology. 2005. 152:355

Handisurya A, G. Pacini, A. Tura, A. Gessl, A. Kautzky-Willer. Effects of T4 replacement therapy on glucose metabolism in subjects with subclinical (SH) and overt hypothyroidism (OH). Clinical endocrinology. 2008. 69:963

Ilic S, M. Tadic, B. Ivanovic, Z. Caparevic, B. Trbojevic, V. Celic. Left and right ventricular structure and function in subclinical hypothyroidism: The effects of one-year levothyroxine treatment. Medical Science Monitor. 2013. 19:960

Inoue K, Tetsuro Tsujimoto, Jun Saito, Takehiro Sugiyama. Association Between Serum Thyrotropin Levels and Mortality Among Euthyroid Adults in the United States.. Thyroid. 2016. 26:1457

Iqbal A, H. Schirmer, P. Lunde, Y. Figenschau, K. Rasmussen, R. Jorde. Thyroid stimulating hormone and left ventricular function. Journal of Clinical Endocrinology and Metabolism. 2007. 92:3504

Ito M, Takamatsu J, Sasaki I, Hiraiwa T, Fukao A, et al. Disturbed metabolism of remnant lipoproteins in patients with subclinical hypothyroidism. American Journal of Medicine. 2004. 117:696

Jiang F, A. Liu, Y. Lai, X. Yu, C. Li, C. Han, Y. Zhang, X. Wang, Z. Wang, S. Bao, N. Lv, M. Jin, F. Yang, Y. Fan, T. Jin, W. Zhao, Z. Shan, W. Teng. Change in serum TSH levels within the reference range was associated with variation of future blood pressure: A 5-year follow-up study. Journal of human hypertension. 2017. 31:244

Kebapcilar L, Comlekci A, Tuncel P, Solak A, Secil M, et al. Effect of levothyroxine replacement therapy on paraoxonase-1 and carotid intima-media thickness in subclinical hypothyroidism. Medical Science Monitor. 2010. 16:CR41

Kim H, T. H. Kim, H. I. Kim, S. Y. Park, Y. N. Kim, S. Kim, M. -J Kim, S. -M Jin, K. Y. Hur, J. H. Kim, M. -K Lee, Y. -K Min, J. H. Chung, M. Kang, S. W. Kim. Subclinical thyroid dysfunction and risk of carotid atherosclerosis. PLoS ONE. 2017. 12:#pages#

Kim SK, -H Kim S, -S Park K, -W Park S, -W Cho Y. Regression of the increased common carotid artery-intima media thickness in subclinical hypothyroidism after thyroid hormone replacement. Endocrine journal. 2009. 56:753

Lambert CG, Aurelien J. Mazurie, Nicolas R. Lauve, Nathaniel G. Hurwitz, S. Stanley Young, Robert L. Obenchain, Nicolas W. Hengartner, Douglas J. Perkins, Mauricio Tohen, Berit Kerner. Hypothyroidism risk compared among nine common bipolar disorder therapies in a large US cohort.. Bipolar disorders. 2016. 18:247

Langén VL, T. J. Niiranen, P. Puukka, A. O. Lehtonen, J. A. Hernesniemi, J. Sundvall, V. Salomaa, A. M. Jula. Thyroid-stimulating hormone and risk of sudden cardiac death, total mortality and cardiovascular morbidity. Clinical endocrinology. 2018. 88:105

Lee Y, Y. -H Lim, J. -H Shin, J. Park, J. Shin. Impact of subclinical hypothyroidism on clinical outcomes following percutaneous coronary intervention. International journal of cardiology. 2018. 253:155

Lima Carvalho MDF, J. S. De Medeiros, M. M. Valença. Headache in recent onset hypothyroidism: Prevalence, characteristics and outcome after treatment with levothyroxine. Cephalalgia. 2017. 37:938

Lo JC, G. J. Beck, G. A. Kaysen, C. T. Chan, A. S. Kliger, M. V. Rocco, M. Li, G. M. Chertow,for the FHN Study. Thyroid function in end stage renal disease and effects of frequent hemodialysis. Hemodialysis International. 2017. 21:534

Madathil A, Hollingsworth KG, Blamire AM, Razvi S, Newton JL, et al. Levothyroxine improves abnormal cardiac bioenergetics in subclinical hypothyroidism: A cardiac magnetic resonance spectroscopic study. Journal of Clinical Endocrinology and Metabolism. 2015. 100:E607

Mahto M, Chakraborthy B, Gowda SH, Kaur H, Vishnoi G, et al. Are hsCRP levels and LDL/HDL ratio better and early markers to unmask onset of dyslipidemia and inflammation in asymptomatic subclinical hypothyroidism?. Indian Journal of Clinical Biochemistry. 2012. 27:284

Malhotra Y, R. M. Kaushik, R. Kaushik. Echocardiographic evaluation of left ventricular diastolic dysfunction in subclinical hypothyroidism: A case–control study. Endocrine research. 2017. 42:198

Mammen JS, J. McGready, P. W. Ladenson, E. M. Simonsick. Unstable Thyroid Function in Older Adults Is Caused by Alterations in Both Thyroid and Pituitary Physiology and Is Associated with Increased Mortality. Thyroid. 2017. 27:1370

Mariotti S, S. Zoncu, F. Pigliaru, C. Putzu, V. M. Cambuli, S. Vargiu, M. Deidda, G. Mercuro. Cardiac effects of l-thyroxine administration in borderline hypothyroidism. International journal of cardiology. 2008. 126:190

Martin SS, Natalie Daya, Pamela L. Lutsey, Kunihiro Matsushita, Anna Fretz, John W. McEvoy, Roger S. Blumenthal, Josef Coresh, Philip Greenland, Anna Kottgen, Elizabeth Selvin. Thyroid Function, Cardiovascular Risk Factors, and Incident Atherosclerotic Cardiovascular Disease: The Atherosclerosis Risk in Communities (ARIC) Study.. Journal of Clinical Endocrinology & Metabolism. 2017. 102:3306

Milionis HJ, Efstathiadou Z, Tselepis AD, Bairaktari ET, Tsironis LD, et al. Lipoprotein (a) levels and apolipoprotein (a) isoform size in patients with subclinical hypothyroidism: Effect of treatment with levothyroxine. Thyroid. 2003. 13:365

Mu G, Mu X, Xing H, Xu R, Sun G, et al. Subclinical hypothyroidism as an independent risk factor for colorectal neoplasm. Clinics and Research in Hepatology and Gastroenterology. 2015. 39:261

Nacar AB, Acar G, Yorgun H, Akçay A, Özkaya M, et al. The effect of antithyroid treatment on atrial conduction times in patients with subclinical hyperthyroidism. Echocardiography. 2012. 29:950

Nagasaki T, Inaba M, Yamada S, Kumeda Y, Hiura Y, et al. Changes in brachial-ankle pulse wave velocity in subclinical hypothyroidism during normalization of thyroid function. Biomedicine and Pharmacotherapy. 2007. 61:482

Nordio M, S. Basciani. Myo-inositol plus selenium supplementation restores euthyroid state in Hashimoto's patients with subclinical hypothyroidism.. European Review for Medical & Pharmacological Sciences. 2017. 21:51

Oflaz H, R. Kurt, F. Sen, I. Onur, A. O. Cimen, A. Elitok, K. Turkmen, B. Pamukcu, E. Kasikcioglu, Z. Bugra, F. Mercanoglu, N. Ozbey. Coronary flow reserve after L-thyroxine therapy in Hashimoto's thyroiditis patients with subclinical and overt hypothyroidism. Endocrine. 2007. 32:264

Oner FA, S. Yurdakul, E. Oner, A. Kubat Uzum, M. Erguney. Evaluation of the eff ect of l-thyroxin therapy on cardiac functions by using novel tissue Doppler-derived indices in patients with subclinical hypothyroidism. Acta Cardiologica. 2011. 66:47

Oner FA, Yurdakul S, Oner E, Uzum AK, Erguney M. Evaluation of the effect of L-thyroxin therapy on endothelial functions in patients with subclinical hypothyroidism. Endocrine. 2011. 40:280

Rhee CM, Y. Chen, A. S. You, S. M. Brunelli, C. P. Kovesdy, M. J. Budoff, G. A. Brent, K. Kalantar-Zadeh, D. V. Nguyen. Thyroid status, quality of life, and mental health in patients on hemodialysis. Clinical Journal of the American Society of Nephrology. 2017. 12:1274

Ripoli A, A. Pingitore, B. Favilli, A. Bottoni, S. Turchi, N. F. Osman, D. De Marchi, M. Lombardi, A. L'Abbate, G. Iervasi. Does subclinical hypothyroidism affect cardiac pump performance? Evidence from a magnetic resonance imaging study. Journal of the American College of Cardiology. 2005. 45:439

Rodondi N, D. C. Bauer, A. R. Cappola, J. Cornuz, J. Robbins, L. P. Fried, P. W. Ladenson, E. Vittinghoff, J. S. Gottdiener, A. B. Newman. Subclinical Thyroid Dysfunction, Cardiac Function, and the Risk of Heart Failure. The Cardiovascular Health Study. Journal of the American College of Cardiology. 2008. 52:1152

Rodondi N, Den Elzen WPJ, Bauer DC, Cappola AR, Razvi S, et al. Subclinical hypothyroidism and the risk of coronary heart disease and mortality. JAMA - Journal of the American Medical Association. 2010. 304:1365

Romano MMD, L. M. Z. MacIel, O. C. Almeida-Filho, A. Pazin-Filho, A. Schmidt, B. C. MacIel. Myocardial ultrasonic tissue characterization in patients with thyroid dysfunction. Cardiovascular Ultrasound. 2010. 8:#pages#

Rosario PW, Carvalho M, Calsolari MR. Symptoms of thyrotoxicosis, bone metabolism and occult atrial fibrillation in older women with mild endogenous subclinical hyperthyroidism. Clinical endocrinology. 2016. 85:132

Rosario PWS, M. R. Calsolari. Impact of subclinical hypothyroidism with TSH =10 mIU/L on glomerular filtration rate in adult women without known kidney disease. Endocrine. 2018. 59:694

Rotondi M, Leporati P, Rizza MI, Clerici A, Groppelli G, et al. Raised serum TSH in morbid-obese and non-obese patients: effect on the circulating lipid profile. Endocrine. 2014. 45:92.

Sato Y, A. Yoshihisa, Y. Kimishima, T. Kiko, S. Watanabe, Y. Kanno, S. Abe, M. Miyata, T. Sato, S. Suzuki, M. Oikawa, A. Kobayashi, T. Yamaki, H. Kunii, K. Nakazato, T. Ishida, Y. Takeishi. Subclinical Hypothyroidism Is Associated With Adverse Prognosis in Heart Failure Patients. Canadian Journal of Cardiology. 2018. 34:80

Sayin I, Erkan AF, Ekici B, Kutuk U, Corakci A, et al. Thickening of the epicardial adipose tissue can be alleviated by thyroid hormone replacement therapy in patients with subclinical hypothyroidism. Kardiologia polska. 2016. 74:1492

Schultheiss UT, N. Daya, M. E. Grams, J. Seufert, M. Steffes, J. Coresh, E. Selvin, A. Köttgen. Thyroid function, reduced kidney function and incident chronic kidney disease in a community-based population: The Atherosclerosis Risk in Communities study. Nephrology Dialysis Transplantation. 2017. 32:1874

Segna D, Mean M, Limacher A, Baumgartner C, Blum MR, et al. Association between thyroid dysfunction and venous thromboembolism in the elderly: a prospective cohort study. Journal of Thrombosis & Haemostasis. 2016. 14:685

Serter R, Demirbas B, Korukluoglu B, Culha C, Cakal E, et al. The effect of L-thyroxine replacement therapy on lipid based cardiovascular risk in subclinical hypothyroidism. Journal of endocrinological investigation. 2004. 27:897

Shatynska-Mytsyk I, Rodrigo L, Cioccocioppo R, Petrovic D, Lakusic N, et al. The impact of thyroid hormone replacement therapy on left ventricular diastolic function in patients with subclinical hypothyroidism. Journal of endocrinological investigation. 2016. 39:709

Sommat K, Whee Sze Ong, Ashik Hussain, Yoke Lim Soong, Terence Tan, Joseph Wee, Kam Weng Fong. Thyroid V40 Predicts Primary Hypothyroidism After Intensity Modulated Radiation Therapy for Nasopharyngeal Carcinoma.. International journal of radiation oncology, biology, physics. 2017. 98:574

Surkovic I, I. Suljevic, A. Kudumovic. Therapy effect on ejection fraction in patients with hypo- and hyperthyroidism. HealthMED. 2010. 4:610

Taddei S, Caraccio N, Virdis A, Dardano A, Versari D, et al. Impaired endothelium-dependent vasodilatation in subclinical hypothyroidism: Beneficial effect of levothyroxine therapy. Journal of Clinical Endocrinology and Metabolism. 2003. 88:3731

Tadic M, S. Ilic, B. Ivanovic, V. Celic. Left atrial phasic function and mechanics in women with subclinical hypothyroidism: The effects of levothyroxine therapy. Echocardiography. 2014. 31:1221

Tadic M, S. Ilic, N. Kostic, Z. Caparevic, V. Celic. Subclinical hypothyroidism and left ventricular mechanics: A three-dimensional speckle tracking study. Journal of Clinical Endocrinology and Metabolism. 2014. 99:307

Tadic M, S. Ilic, V. Celic. Right ventricular and right atrial function and deformation in patients with subclinical hypothyroidism: A two- and three-dimensional echocardiographic study. European Journal of Endocrinology. 2014. 170:77

Tanase DM, Vulpoi C, Ionescu SD, Ouatu A, Ambarus V, et al. Effects of subclinical and overt primary hypothyroidism on the cardiac function and their reversibility under treatment using tissue doppler echocardiography. Acta Endocrinologica. 2014. 10:640

Tohidi M, A. Derakhshan, S. Akbarpour, A. Amouzegar, L. Mehran, A. Baghbani-Oskouei, F. Azizi, F. Hadaegh. Thyroid Dysfunction States and Incident Cardiovascular Events: The Tehran Thyroid Study. Hormone and Metabolic Research. 2018. 50:37

Traub-Weidinger T, Graf S, Beheshti M, Ofluoglu S, Zettinig G, et al. Coronary vasoreactivity in subjects with thyroid autoimmunity and subclinical hypothyroidism before and after supplementation with thyroxine. Thyroid. 2012. 22:245

Tseng FY, -Y Lin W, -C Lin C, -T Lee L, -C Li T, et al. Subclinical hypothyroidism is associated with increased risk for all-cause and cardiovascular mortality in adults. Journal of the American College of Cardiology. 2012. 60:730

Tseng FY, -Y Lin W, -I Li C, -C Li T, Lin C-C, et al. Subclinical hypothyroidism is associated with increased risk for cancer mortality in adult taiwanese-A 10 years population- Based cohort. PLoS ONE. 2015. 10:#pages#

Tuliani TA, M. Shenoy, K. Belgrave, A. Deshmukh, S. Pant, A. Hilliard, L. Afonso. Role of Microalbuminuria in Predicting Cardiovascular Mortality in Individuals With Subclinical Hypothyroidism. American Journal of the Medical Sciences. 2017. 354:285

Turhan S, C. Tulunay, M. O. Cin, A. Gursoy, M. Kilickap, I. Dincer, B. Candemir, S. Gullu, C. Erol. Effects of thyroxine therapy on right ventricular systolic and diastolic function in patients with subclinical hypothyroidism: A study by pulsed wave tissue doppler imaging. Journal of Clinical Endocrinology and Metabolism. 2006. 91:3490

Virgini VS, L. W. Wijsman, N. Rodondi, D. C. Bauer, P. M. Kearney, J. Gussekloo, W. P. J. Den Elzen, J. W. Jukema, R. G. J. Westendorp, I. Ford, D. J. Stott, S. P. Mooijaart. Subclinical thyroid dysfunction and functional capacity among elderly. Thyroid. 2014. 24:208

Virgini VS, Wijsman LW, Rodondi N, Bauer DC, Kearney PM, et al. Subclinical thyroid dysfunction and functional capacity among elderly. Thyroid. 2014. 24:208.

Vishnoi G, Chakraborty B, Garda H, Gowda SH, Goswami B. Low mood and response to Levothyroxine treatment in Indian patients with subclinical hypothyroidism. Asian Journal of Psychiatry. 2014. 8:89

Viswanathan G, Balasubramaniam K, Hardy R, Marshall S, Zaman A, et al. Blood thrombogenicity is independently associated with serum TSH levels in post-non-ST elevation acute coronary syndrome. Journal of Clinical Endocrinology and Metabolism. 2014. 99:E1050

Walsh JP, A. P. Bremner, M. K. Bulsara, P. O'Leary, P. J. Leedman, P. Feddema, V. Michelangeli. Subclinical thyroid dysfunction as a risk factor for cardiovascular disease. Archives of Internal Medicine. 2005. 165:2467

Wang H, A. Liu, Y. Zhou, Y. Xiao, Y. Yan, T. Zhao, X. Gong, T. Pang, C. Fan, J. Zhao, W. Teng, Z. Shan, Y. Lai. The correlation between serum free thyroxine and regression of dyslipidemia in adult males: A 4.5-year prospective study. Medicine (United States). 2017. 96:#pages#

Xiangwen Cheng, Zixiao Wei, Guangde Zhang, Xin Shao, Bo Li, et al. A systematic review of combinatorial treatment with warming and invigorating drugs and levothyroxine for hypothyroidism caused by Hashimoto disease.. Annals of Translational Medicine. 2016. 4:459

Xu G, Y. Yan, Y. Liu. The cardiovascular disease risks of nonthyroidal illness syndrome and inflammatory responses on patients with chronic kidney disease: From the association to clinical prognosis. Cardiovascular Therapeutics. 2014. 32:257

Yang GR, -K Yang J, Zhang L, -H An Y, -K Lu J. Association between subclinical hypothyroidism and proliferative diabetic retinopathy in type 2 diabetic patients: A case-control study. Tohoku Journal of Experimental Medicine. 2010. 222:303

Yazıcı D, Özben B, Toprak A, Yavuz D, Aydın H, et al. Effects of restoration of the euthyroid state on epicardial adipose tissue and carotid intima media thickness in subclinical hypothyroid patients. Endocrine. 2015. 48:909

Yin JJ, -M Liao L, -X Luo D, Xu K, -H Ma S, et al. Spatial working memory impairment in subclinical hypothyroidism: An fMRI study. Neuroendocrinology. 2013. 97:260

Zhou Y, Chen Y, Cao X, Liu C, Liu C, et al. Association between plasma homocysteine status and hypothyroidism: A meta-analysis. International Journal of Clinical and Experimental Medicine. 2014. 7:4544

**Wrong population: n = 23**

Aghili R, Khamseh ME, Malek M, Hadian A, Baradaran HR, et al. Changes of subtests of Wechsler Memory Scale and cognitive function in subjects with subclinical hypothyroidism following treatment with levothyroxine. Archives of Medical Science. 2012. 8:1096

Avais Jabbar, Lorna Ingoe, Simon Pearce, Azfar Zaman, Salman Razvi. Thyroxine in acute myocardial infarction (ThyrAMI) - levothyroxine in subclinical hypothyroidism post-acute myocardial infarction: study protocol for a randomised controlled trial.. Trials [Electronic Resource]. 2015. 16:115

Bestwick JP, John R, Maina A, Guaraldo V, Joomun M, et al. Thyroid stimulating hormone and free thyroxine in pregnancy: Expressing concentrations as multiples of the median (moms). Clinica Chimica Acta. 2014. 430:33

Birte Nygaard. Hyperthyroidism (primary). Clinical Evidence. 2008.

Birte Nygaard. Hyperthyroidism (primary). Clinical Evidence. 2010.

Buscemi S, Verga S, Cottone S, Andronico G, D'Orio L, et al. Favorable clinical heart and bone effects of anti-thyroid drug therapy in endogenous subclinical hyperthyroidism. Journal of endocrinological investigation. 2007. 30:230

Cappelli C,​ Pirola I,​ Daffini L,​ Formenti A,​ Iacobello C,​ et al. A Double-Blind Placebo-Controlled Trial of Liquid Thyroxine Ingested at Breakfast: Results of the TICO Study. Thyroid. 2016. 26:197

Chen Y, G. Wu, M. Xu. The effect of l-thyroxine substitution on oxidative stress in early-stage diabetic nephropathy patients with subclinical hypothyroidism: a randomized double-blind and placebo-controlled study. International urology and nephrology. 2018. 50:97

Christ-Crain M, C. Meier, P. R. Huber, J. -J Staub, B. Müller. Effect of L-thyroxine replacement therapy on surrogate markers of skeletal and cardiac function in subclinical hypothyroidism. Endocrinologist. 2004. 14:161

Christ-Crain M, Meier C, Guglielmetti M, Huber PR, Riesen W, et al. Elevated C-reactive protein and homocysteine values: cardiovascular risk factors in hypothyroidism? A cross-sectional and a double-blind, placebo-controlled trial. Atherosclerosis. 2003. 166:379

Christ-Crain M, Morgenthaler NG, Meier C, Muller C, Nussbaumer C, et al. Pro-A-type and N-terminal pro-B-type natriuretic peptides in different thyroid function states. Swiss medical weekly. 2005. 135:549

Coceani M, Molinaro S, Scalese M, Landi P, Carpeggiani C, et al. Thyroid hormone, amiodarone therapy, and prognosis in left ventricular systolic dysfunction.. Journal of endocrinological investigation. 2011. 34:e144.

Drechsler C, Schneider A, Gutjahr-Lengsfeld L, Kroiss M, Carrero JJ, et al. Thyroid function, cardiovascular events, and mortality in diabetic hemodialysis patients. American Journal of Kidney Diseases. 2014. 63:988.

Kong WM, Sheikh MH, Lumb PJ, Naoumova RP, Freedman DB, et al. A 6-month randomized trial of thyroxine treatment in women with mild subclinical hypothyroidism. The American Journal of Medicine. 2002. 112:348

Koroglu BK, Bagci O, Ersoy IH, et al. Effects of levothyroxine treatment on cardiovascular risk profile and carotid intima media thickness in patients with subclinical hypothyroidism. Acta Endocrinologica. 2012;VIII(3):433-442.

Lillevang-Johansen M, B. Abrahamsen, H. L. Jørgensen, T. H. Brix, L. Hegedüs. Over-and Under-Treatment of Hypothyroidism Is Associated with Excess Mortality: A Register-Based Cohort Study. Thyroid. 2018. 28:566

Lu Y, H. Guo, D. Liu, Z. Zhao. Preservation of renal function by thyroid hormone replacement in elderly persons with subclinical hypothyroidism. Archives of Medical Science. 2016. 12:772

Meier C, J. -J Staub, C. -B Roth, M. Guglielmetti, M. Kunz, A. R. Miserez, J. Drewe, P. Huber, R. Herzog, B. Müller. TSH-controlled L-thyroxine therapy reduces cholesterol levels and clinical symptoms in subclinical hypothyroidism: A double blind, placebo-controlled trial (basel thyroid study). Journal of Clinical Endocrinology and Metabolism. 2001. 86:4860

Mitchell JE, Hellkamp AS, Mark DB, Anderson J, Johnson GW, et al. Thyroid function in heart failure and impact on mortality.. JACC Heart Failure. 2013. 1:48.

Razvi S, Ingoe L, Keeka G, Oates C, McMillan C, et al. The beneficial effect of L-thyroxine on cardiovascular risk factors, endothelial function, and quality of life in subclinical hypothyroidism: randomized, crossover trial. The Journal of clinical endocrinology and metabolism. 2007. 92:1715

Seo C, S. Kim, M. Lee, M. -U Cha, H. Kim, S. Park, H. -R Yun, J. H. Jhee, Y. K. Kee, S. H. Han, T. -H Yoo, S. -W Kang, J. T. Park. THYROID HORMONE REPLACEMENT REDUCES the RISK of CARDIOVASCULAR DISEASES in DIABETIC NEPHROPATHY PATIENTS with SUBCLINICAL HYPOTHYROIDISM. Endocrine Practice. 2018. 24:265

Yonem O, Dokmetas HS, Aslan SM, Erselcan T. Is antithyroid treatment really relevant for young patients with subclinical hyperthyroidism?. Endocrine journal. 2002. 49:307

Zimmermann MB, Aeberli I, Melse-Boonstra A, Grimci L, Bridson J, et al. Iodine treatment in children with subclinical hypothyroidism due to chronic iodine deficiency decreases thyrotropin and c-peptide concentrations and improves the lipid profile. Thyroid. 2009. 19:1099

**Wrong outcome: n = 20**

Abu-Helalah M,​ Law MR,​ Bestwick JP,​ Monson JP,​ Wald NJ. A randomized double-blind crossover trial to investigate the efficacy of screening for adult hypothyroidism. *Journal of medical screening.* 2010. 17:164

Andries A, Isaakidis P, Das M, Khan S, Paryani R, et al. High rate of hypothyroidism in multidrug-resistant tuberculosis patients co-infected with HIV in Mumbai, India. *PLoS ONE [Electronic Resource].* 2013. 8:e78313.

Bilgir O, Bilgir F, Calan M, Calan OG, Yuksel A. Comparison of pre- and post-levothyroxine high-sensitivity c-reactive protein and fetuin-a levels in subclinical hypothyroidism. *Clinics (Sao Paulo, Brazil).* 2015. 70:97.

Carvalho GA, Perez CL, Ward LS. The clinical use of thyroid function tests.. *Arquivos Brasileiros de Endocrinologia e Metabologia.* 2013. 57:193.

Ilic S, Tadic M, Ivanovic B, Caparevic Z, Trbojevic B, et al. Left and right ventricular structure and function in subclinical hypothyroidism: the effects of one-year levothyroxine treatment. *Medical Science Monitor.* 2013. 19:960.

Kaptein EM, LoPresti JS, Kaptein MJ. Is an isolated TSH elevation in chronic nonthyroidal illness "subclinical hypothyroidism"?. *Journal of Clinical Endocrinology & Metabolism.* 2014. 99:4015.

Kozielewicz D, Zalesna A, Dybowska D. Can pegylated interferon alpha 2a cause development of thyroid disorders in patients with chronic hepatitis B?. *Expert Opinion on Drug Safety.* 2014. 13:1009.

Kyritsi EM, Yiakoumis X, Pangalis GA, Pontikoglou C, Pyrovolaki K, et al. High Frequency of Thyroid Disorders in Patients Presenting With Neutropenia to an Outpatient Hematology Clinic STROBE-Compliant Article.. *Medicine.* 2015. 94:e886.

Lankhaar JA, de Vries WR, Jansen JA, Zelissen PM, Backx FJ. Impact of overt and subclinical hypothyroidism on exercise tolerance: a systematic review. *Research Quarterly for Exercise & Sport.* 2014. 85:365.

Liu L, Yu Y, Zhao M, Zheng D, Zhang X, Guan Q, et al. Benefits of levothyroxine replacement therapy on nonalcoholic fatty liver disease in subclinical hypothyroidism patients. Intl J Endocrinol 2017;2017.

Lupoli R, Di Minno MN, Tortora A, Scaravilli A, Cacciapuoti M, et al. Primary and Secondary Hemostasis in Patients With Subclinical Hypothyroidism: Effect of Levothyroxine Treatment. *Journal of Clinical Endocrinology & Metabolism.* 2015. 100:2659.

Madathil A, Hollingsworth KG, Blamire AM, Razvi S, Newton JL, et al. Levothyroxine improves abnormal cardiac bioenergetics in subclinical hypothyroidism: a cardiac magnetic resonance spectroscopic study. *Journal of Clinical Endocrinology & Metabolism.* 2015. 100:E607.

Mainenti MRM, P. F. S. Teixeira, F. P. Oliveira, M. Vaisman. Effect of hormone replacement on exercise cardiopulmonary reserve and recovery performance in subclinical hypothyroidism. Brazilian Journal of Medical and Biological Research. 2010. 43:1095

Martins RM,​ Fonseca RH,​ Duarte MM,​ Reuters VS,​ Ferreira MM,​ et al. Impact of subclinical hypothyroidism treatment in systolic and diastolic cardiac function. *Arquivos Brasileiros de Endocrinologia e Metabologia.* 2011. 55:460

McNamee T, Hyland T, Harrington J, Cadogan S, Honari B, et al. Haematinic deficiency and macrocytosis in middle-aged and older adults. *PLoS ONE [Electronic Resource].* 2013. 8:e77743.

Pirola I,​ Gandossi E,​ Agosti B,​ Delbarba A,​ Cappelli C. Selenium supplementation could restore euthyroidism in subclinical hypothyroid patients with autoimmune thyroiditis. *Endokrynologia Polska.* 2016. 67:567

Shinkov A, Borissova AM, Vlahov J, Dakovska L, Blajeva E. Male gender differences in the thyroid ultrasound features, thyroid peroxidase antibodies and thyroid hormone levels: a large population-based study. *Journal of endocrinological investigation.* 2014. 37:269.

Tadic M, Ilic S, Ivanovic B, Celic V. Left atrial phasic function and mechanics in women with subclinical hypothyroidism: the effects of levothyroxine therapy.. *Echocardiography.* 2014. 31:1221.

Tomella C, Catanzaro R, Illuzzi N, Cabeca A, Zerbinati N, et al. The hidden phenomenon of oxidative stress during treatment of subclinical-mild hypothyroidism: a protective nutraceutical intervention. *Rejuvenation Research.* 2014. 17:180.

Zhou Y, Chen Y, Cao X, Liu C, Xie Y. Association between plasma homocysteine status and hypothyroidism: a meta-analysis. *International journal of clinical and experimental medicine.* 2014. 7:4544.

**Observation study reporting on outcomes for which data is available from one or more RCTs: n = 2**

Grossman A, Feldhamer I, Meyerovitch J. Treatment with levothyroxin in subclinical hypothyroidism is associated with increased mortality in the elderly. Eur J Intern Med 2018;50:65-68.

Shin DH,​ Lee MJ,​ Kim SJ,​ Oh HJ,​ Kim HR,​ et al. Preservation of renal function by thyroid hormone replacement therapy in chronic kidney disease patients with subclinical hypothyroidism. *Journal of Clinical Endocrinology and Metabolism.* 2012. 97:2732

# Appendix 6: List of Excluded Studies for KQ5 after Full-text Screening

**Wrong study design: n = 2**

Schwartz E, Holtorf K. Hormone Replacement Therapy in the Geriatric Patient: Current State of the Evidence and Questions for the Future. Estrogen, Progesterone, Testosterone, and Thyroid Hormone Augmentation in Geriatric Clinical Practice: Part 1. *Clinics in geriatric medicine.* 2011. 27:541

Werhun A, Hamilton W. Are we overusing thyroid function tests?. *British Journal of General Practice.* 2013. 63:404

**Wrong intervention: n = 1**

Pollock AM, Sturrock A, Marshall K, Davidson KM, et al. Thyroxine treatment in patients with symptoms of hypothyroidism but thyroid function tests within the reference range: Randomised double blind placebo controlled crossover trial. *British medical journal.* 2001. 323:891

**Wrong outcome: n = 1**

Verburg FA, Grelle I, Tatschner K, Reiners C, Luster M. Prevalence of thyroid disorders in elderly people in Germany: A screening study in a country with endemic goitre. *Nuklearmedizin.* 2017. 56:9

# Appendix 7: Individual Study Characteristics

| **Lead author, Publication Year, Country, number of participants (% females)** | **Study Design , comparison, follow-up, clinic setting** | **Participants** | **Intervention** | **Outcomes** |
| --- | --- | --- | --- | --- |
| Stott, 2017,  UK,  n=737 (54% female) | RCT,  Placebo,  1-3 years,  outpatient clinic | *Inclusion criteria:* Age ≥ 65 years; persistent SCHo as defined by an elevated TSH (4.60 to 19.99 mIU/L) measured on ≥ 2 occasions that were 3 months to 3 years apart, with FT_4_ WNL  *Exclusion criteria:* Current prescription for LT_4_, antithyroid drugs, amiodarone, or lithium; thyroid surgery or receipt of radioactive iodine within previous 12 months; dementia; hospitalization for a major illness or an elective surgery within previous 4 weeks; acute coronary syndrome within previous 4 weeks; terminal illness | LT_4_ treatment (initial: 25-50 µg/day, titrated to achieve TSH WNL) | *Primary:* Change in ThyPRO measure Hypothyroid Symptoms score and the Tiredness score  *Secondary:* Changes in generic health-related QoL; comprehensive thyroid-related QoL, handgrip strength, executive cognitive function, BP, weight, BMI, waist circumference, activities of daily living, fatal and non-fatal CV events  SAEs, AEs of special interest, WDAEs |
| Zhao, 2016, China  n=378 (73% female) | RCT,  No treatment,  15 months | *Inclusion criteria:* Chinese residents of Ningyang County, Shandong Province of China; age ≥ 40 years; mild SCHo as defined by TSH of 4.2-10.0 mIU/L and FT_4_ WNL based on two measurements taken 3 months apart  *Exclusion criteria:* Pregnant or lactating females; complications or conditions that affect thyroid status or lipid metabolism; taking medications within previous 3 months that affect thyroid or lipid metabolism; poor compliance. | LT_4_ treatment (initial: 25 µg/day, titrated to achieve clinical euthyroidism) | *Primary:* Change in serum total cholesterol  *Secondary*: Change in other serum lipid parameters  SBP, DBP, AEs |
| Liu, 2015,  China  n=136 (74% female) | RCT,  Placebo,  48 weeks, outpatient clinic | *Inclusion criteria:* Age <65 years; type 2 diabetes per WHO diagnostic criteria (1999); diabetic nephropathy; SCHo due to Hashimoto’s thyroiditis; TSH 4.0– 7.0 mIU/L; TPO-Ab >50 U/ml; FT_3_, FT_4_ WNL; urinary albumin excretion rate 20–199 mg/min; adequate glycemic control; stable illness condition; no concurrent treatment with angiotensin converting enzyme inhibitors, angiotensin receptor blockers, uric acid synthesis inhibitors, uricosuric agents, or lipid-lowering drugs for ≥ 3 months  *Exclusion criteria:* Primary renal diseases; previous history of thyroid diseases and use of drugs affecting thyroid function; association with hypertension, severe dyslipidemia, acute metabolic disorders in diabetes, malignancy, or severe cardiac, hepatic, and cerebral diseases | LT_4_ treatment (initial: 12.5 µg/day, titrated to achieve TSH WNL) | *Primary:* change in urinary albumin excretion rate  *Secondary (of interest only)*: change in SBP, DBP, lipids  BMI, AEs |
| Najafi, 2015,  Iran  n=60 (85% female) | RCT,  Placebo,  12 weeks, outpatient clinic | *Inclusion criteria:* SCHo as defined by TSH > 4.5 mIU/L, FT_4_ WNL, positive anti-TPO-Ab  *Exclusion criteria:* History of endocrine or autoimmune diseases other than SCHo; history of thyroid hormone or corticosteroid replacement in previous 2 months; co-morbid diabetes mellitus, heart failure, chronic liver or pulmonary disorder; history of head trauma or seizure; known psychological or mental disorders; pregnancy | LT_4_ treatment (100 µg/day) | *Main outcome:* Depression score |
| Reuters, 2012, Brazil  n=71 (87% female) | RCT,  Placebo,  6 months, outpatient clinic | *Inclusion criteria:* SCHo as defined by TSH > 4.0 µUI/ml and FT_4_ WNL measured twice at least six weeks apart; if SCHo developed after hyperthyroidism treatment, confirmation of euthyroidism for ≥ 1 year beforehand  *Exclusion criteria:* Chronic diseases or medications that could affect thyroid or neuromuscular function; severe psychiatric disturbances; school attendance for < 3 years | LT_4_ treatment (initial: 25-75 µg/day titrated to achieve TSH WNL) | Primary and secondary outcomes not explicitly stated; however, generic health-related QoL, psychiatric symptoms, and muscular function appear to be the main outcomes |
| Cabral, 2011, Brazil  n=32 (100% female) | RCT,  No treatment,  1 year, outpatient clinic | *Inclusion criteria:* Female patients with mild SCHo; elevated TSH of 4.0 to 12 mIU/L and FT_4_ WNL documented at least twice and at  least six weeks apart; no previous history of thyroid disease  *Exclusion criteria:* History of alcohol use; any cardiovascular disease or concomitant non-thyroid illnesses | LT_4_ treatment (initial: 0.75 µg/kg/day titrated to achieve TSH, FT_4_ WNL | Primary and secondary outcomes not explicitly stated; however, endothelial function and carotid artery intima-media thickness appear to be the main outcomes.  Lipid levels also reported |
| Parle, 2010,  UK  n=94 (61% female) | RCT,  Placebo,  12 months, primary care clinic | *Inclusion criteria:* Age ≥ 65 years; SCHo with TSH > 5.5 mU/L and FT_4_ WNL; registered with one of 20 family practices in the greater Birmingham area of the UK  *Exclusion criteria:* Current LT_4_ or antithyroid treatment or recent treatment for hyperthyroidism | LT_4_ treatment (initial: 25 µg/day titrated to achieve TSH WNL) | Main outcome: cognitive function, depression score  WDAEs |
| Mainenti,  2009, Brazil  n=23 (100% female) | RCT,  No treatment, 6 months, university hospital outpatient endocrine clinic | *Inclusion criteria*: Untreated females, aged between 30 and 60 years old, presenting elevated serum TSH levels (>4.0 mU/l) and FT4 values within the normal range (0.8-1.9 ng/dL) in two blood samples (interval between samples: ≥ 4 weeks).  *Exclusion criteria*: Use of drugs that could influence thyroid function, heart rate and blood pressure; diagnosed cardiac diseases or systemic arterial hypertension; presence  of pain or other physical problems that could interfere with walking | LT_4_ treatment (initial: 0.75 µg/kg/day titrated to achieve TSH WNL) | Outcomes not pre-specified; the following relevant outcomes were presented: SBP, DBP |
| Nagasaki, 2009, Japan  n=95 (100% female) | RCT,  Placebo,  5 months, outpatient clinic | *Inclusion criteria:* Consecutively recruited females with SCHo due to autoimmune chronic thyroiditis; TSH > upper limit of normal with FT_3_ and FT_4_ WNL;  *Exclusion criteria:* Hypertension; hyperlipidemia; diabetes mellitus; concurrent treatment with hormone replacement therapy, lipid-lowering, antihypertensive, or anti-platelet drugs, or bisphosphonates or etidronate | LT_4_ treatment (initial: 12.5 µg/day titrated to achieve TSH WNL) | Main outcome: arterial stiffness  Lipid profile, SBP, DBP, BMI also reported  WDAEs also reported |
| Mikhail, 2008, Kuwait  n=120 (98% female) | RCT,  Placebo,  52 weeks, outpatient clinic | *Inclusion criteria:* Age between 15 and 60 years; TSH > 4.0 mIU/L and < 10 mIU/L on 2 separate tests with FT_4_ WNL  *Exclusion criteria:* History of thyroid disease; history of radioiodine treatment or any thyroid medication; dyslipidemia or use of lipid-lowering agents during the year before enrollment; cigarette smoking; coronary artery disease; diabetes; renal or hepatic failure; other systemic diseases | LT_4_ treatment (initial: 25 µg/day titrated to achieve TSH WNL) | Main outcome: lipid profile |
| Texeira (a), 2008, Brazil  n=60 (95% female) | RCT,  Placebo,  6 months, outpatient clinic | *Inclusion criteria*: TSH > 4.0 µUI/ml on two occasions within a minimal interval of 6 weeks and FT_4_ WNL; patients with a history of hyperthyroidism needed to be clinically euthyroid for at least one year prior to developing SCHo.  *Exclusion criteria*: ‘Other diseases’; use of drugs that affect thyroid function or lipid profile; BMI ≥ 30 kg/m^2^ | LT_4_ treatment (25-75 µg/day titrated to achieve TSH, FT_4_ WNL) | Primary outcome: lipid profile  BMI, WDAEs |
| Texeira (b) 2008, Brazil  n=60 (95% female) | RCT,  Placebo,  12 months, outpatient clinic |  |  |  |
| Duman, 2007, Turkey  n=63 (100% female) | RCT,  No treatment, 8 months, outpatient clinic | *Inclusion criteria:* Criteria not specified; however, study enrolled pre-menopausal, non-pregnant females with newly diagnosed SCHo (TSH > 4.2 µU/ml) and positive antithyroid antibodies; Hashimoto’s thyroiditis  *Exclusion criteria:* Smoking; obesity (BMI >30 kg/m^2^); diabetes mellitus; hypertension; coronary artery disease; renal or hepatic failure; familial hypercholesterolemia | LT_4_ treatment (initial: 25 µg/day, titrated to achieve euthyroidism) | Main outcomes: endothelial function, lipid profile |
| Fadeyev, 2006, Russia  n=33 (88% female) | RCT,  No treatment, 6 months, outpatient clinic | *Inclusion criteria:* SCHo as defined by TSH > 4.0 mIU/L and FT_4_ WNL measured twice; clinically-proven cardiovascular disease  *Exclusion criteria:* Diabetes or impaired glucose tolerance; myocardial infarction within previous 6 months; history of amiodarone use; cardiac insufficiency; severe oncologic or other somatic disease; liver or kidney insufficiency | LT_4_ treatment (initial: 25 µg/day, titrated to achieve TSH WNL) | *Main outcomes:* No relevant efficacy outcomes  AEs also reported |
| Iqbal, 2006, Norway  n=64 (48% female) | RCT,  Placebo,  12 months, outpatient clinic | *Inclusion criteria:* Age ≥ 30 years; living in the municipality of Tromsø; serum TSH between 3.5 and 10 mIU/L and FT_3_ and FT_4_ WNL; no obvious clinical symptoms of hypothyroidism  *Exclusion criteria:* Age > 80 years; history of coronary infarction, angina pectoris or stroke; using thyroid or lipid-lowering medication | LT_4_ treatment (initial: 50 µg/day, titrated to achieve TSH WNL) | Main outcome: lipid profile  BMI also reported |
| Jorde, 2006, Norway  n=70 (46% female) | RCT,  Placebo,  12 months,  university clinic setting | *Inclusion criteria*: Age ≥ 29 years; living in municipality of Trømso; either participated in the second phase of the 4^th^ Trømso study or became 30, 40, 45, 60, or 75 years old during 2001; completed a health questionnaire; with TSH between 3.5-10.0 mIU/L (FT_3_, FT_4_ WNL) and no obvious symptoms of hypothyroidism.  *Exclusion criteria*: Self-reported history of coronary infarction, angina pectoris, or stroke; participation in other follow-up studies; use of thyroid medication; age > 80 years; other serious diseases reported in hospital records. | thyroxine tablets (in 25-, 50-, and 100- µg strength) or matching placebo  (set doses in first 12 weeks followed by individual dose adjustments thereafter to achieve a TSH between 0.5-1.5 mIU/L). | *Primary*: Depressed mood; mental health status/psychological distress; cognitive function; hypothyroid symptoms |
| Caraccio, 2005, Italy  n=23 (91% female) | RCT,  Placebo,  6 months,  outpatient clinic | *Inclusion criteria:* Patients with SCHo as documented by TSH > 3.6 mIU/L and free thyroid hormone levels within normal range; positive antithyroid antibodies; Hashimoto’s thyroiditis  *Exclusion criteria*: neurological, cardiovascular, respiratory, and other systemic diseases | LT_4_ treatment (initial: 25 µg/day, titrated to achieve TSH WNL) | Primary outcomes: energy and metabolic outcomes  BMI also reported |
| Yazici, 2004, Turkey  n=45 (84% female) | RCT,  Placebo,  1 year, cardiology clinic | *Inclusion criteria:* Patients with SCHo and stable elevated serum TSH and normal thyroid hormone levels for ≥ 1 year before enrollment  *Exclusion criteria*: Cardiovascular disease | LT_4_ treatment (initial: 50 µg/day, titrated to achieve TSH WNL) | Primary and secondary outcomes not explicitly stated; however, myocardial function and structure appear to be the main outcomes.  BMI, SBP, and DBP also reported |
| Monzani, 2004, Italy  n=45 (82% female) | RCT,  Placebo,  6 months, outpatient clinic | *Inclusion criteria:* Documented TSH > 3.6 mIU/L for ≥ 6 months before enrollment  *Exclusion criteria:* Age > 55 years; obesity (BMI > 30 kg/m^2^); smoking; hypertension; diabetes mellitus; renal or hepatic failure; other systemic disease | LT_4_ treatment (initial: 25 µg/day, titrated to achieve TSH WNL) | Main outcomes: carotid artery intima-media thickness, lipid profile  BMI also reported |
| Caraccio, 2002, Italy  n=49 (86% female) | RCT,  Placebo,  6 months, outpatient clinic | *Inclusion criteria:* Patients with stable SCHo as documented by TSH > 3.6 mIU/L for ≥ 6 months before the study; positive antithyroid antibody titers  *Exclusion criteria*: Obesity (BMI > 30 kg/m^2^); smoking; primary or secondary dyslipidemia; serum total cholesterol ≥ 7.8 mmol/L; serum triglycerides ≥ 4.6 mmol/L; diabetes mellitus; renal and hepatic failure; other systemic diseases | LT_4_ treatment (initial: 25 µg/day, titrated to achieve TSH WNL) | Main outcome: lipid profile.  BMI also reported |
| Monzani, 2001, Italy  n=20 (90% female) | RCT,  Placebo,  6 months, outpatient internal medicine clinic | *Inclusion criteria:* TSH > 3.6 mIU/L (range: 3.8-12.0 mIU/L) and FT_3_ and FT_4_ WNL; stable elevated serum TSH and normal thyroid hormone levels for ≥ 1 year before enrollment.  *Exclusion criteria*: Cardiovascular and respiratory diseases | LT_4_ treatment (initial: 50 µg/day, titrated to achieve TSH WNL) | Primary and secondary outcomes not explicitly stated; however, myocardial function and structure appear to be the main outcomes.  BMI, SBP, and DBP also reported |
| Andersen,  2016, Denmark  n=1,192 (64% female) | Registry-based retrospective cohort study, time-to-event (median follow-up of 5.6 years), primary care and hospital outpatients | *Inclusion criteria:* Primary care and hospital outpatients with SCHo (TSH >5.0 mIU/L and FT_4_ WNL) aged ≥ 18 years; known heart disease; underwent thyroid function tests between 1997 and 2011; citizens of three distinct regions of Denmark (Copenhagen region; Roskilde; north region of Denmark)  *Exclusion criteria:* History of thyroid disease or thyroid medication or use of medication affecting thyroid function. | Treated: those who initiated LT_4_ during follow-up | *Primary*: all-cause mortality  *Secondary*: all-cause hospital admissions and major CV events |
| Andersen,  2015, Denmark  n=12,212 (80% female) | Registry-based retrospective cohort study, time-to-event (median follow-up of 5.0 years), primary care | *Inclusion criteria:* Primary care patients with SCHo (TSH >5.0 mIU/L and FT_4_ WNL) aged ≥ 18 years; had been referred by their general practitioner for thyroid function tests between January 1 2000 and December 31 2009; citizens of Copenhagen  *Exclusion criteria:* History of thyroid disease or thyroid medication or use of medication affecting thyroid function. | Treated: those who initiated LT_4_ ≤ 6 months from the date of first thyroid function test | *Primary*: all-cause mortality  *Secondary*: myocardial infarction (fatal, non-fatal), CV death |
| Razvi, 2012,  UK  n=4,735 (83% female) | Registry-based retrospective cohort study, time-to-event (median follow-up of 7.6 years), primary care patients | *Inclusion criteria:* Primary care patients with SCHo within the UK General Practitioner Research Database aged ≥ 40 years with first-ever increased serum TSH of 5.01 to 10.00 mIU/L and FT4 WNL  *Exclusion criteria:* History of IHD or cerebrovascular disease; individuals registered with practices that did not fulfill ≥12 months of predefined data quality criteria leading up to their index elevated TSH; poor-quality records (i.e., lack of continuous follow-up or with incomplete or inaccurate data); individuals treated at any time before their index elevated TSH with amiodarone hydrochloride or lithium carbonate or for ≤ 1 year with an oral corticosteroid. | Treated: those who initiated LT4 during follow-up | *Primary:* composite of incident fatal and nonfatal first-recorded diagnosis of ischemic heart disease  *Secondary*: first fatal and nonfatal cerebrovascular disease, all-cause and cause-specific mortality, and new-onset atrial fibrillation. |

**AE**=adverse event; **BMI**=body mass index; **BP**=blood pressure; **CAD**=coronary artery disease; **CV**=cardiovascular; **DBP**=diastolic blood pressure; **FT_3_**=free triiodothyronine; **FT_4_**= free thyroxine; **HDL**=high-density lipoprotein cholesterol; **LDL**=low-density lipoprotein cholesterol; **LT_4_**=levothyroxine; **QoL**=quality of life; **RCT**=randomized controlled trial; **SAE**=serious adverse event; **SBP**=systolic blood pressure; **SCHo**=subclinical hypothyroidism; **TG**=triglyceride; **ThyPRO**=Thyroid-Related Quality-of-Life Patient-Reported Outcome; **TPO-Ab**=thyroid peroxidase antibody; **TSH**=thyroid-stimulating hormone; **WDAE**=withdrawal due to adverse event; **WNL**=within normal limits

# Appendix 8. Individual Study Results

| **Lead author, Publication Year, Country, Number of participants, Comparison** | **Intervention**  **Length of follow-up** | **Between-Group Results(placebo/no treatment vs. treatment )** | **Adverse Events** |
| --- | --- | --- | --- |
| Stott, 2017, UK  n=737  Placebo | LT_4_ treatment (initial: 25-50 µg/day, titrated to achieve TSH WNL)  follow-up of 1-3 years | **All-cause mortality:** HR of 1.91 (95% CI: 0.65-5.60); p=0.236  **Deaths due to CVD:** HR could not be calculated due to small number of events [Placebo group: 1 death (0.3%) vs treatment group: 2 deaths (0.5%)]  **Fatal or non-fatal CV event:** HR of 0.89 (95% CI: 0.47-1.69); p=0.728  **Atrial fibrillation (new-onset):** HR of 0.80 (95% CI: 0.35-1.80); p=NS  **Fractures:** HR of 1.06 (95% CI: 0.41-2.76); p=NS  **ThyPRO hypothyroid symptoms score:**   - At extended follow-up: DF of 1.0 (95% CI: -1.9 to 3.9); p=0.50 - At 12 months: DF of 0.0 (95% CI: -2.0 to 2.1); p=0.99   **ThyPRO tiredness score:**   - At extended follow-up: DF of -3.5 (95% CI: -7.0 to 0.0); p=0.05 - At 12 months: DF of 0.4 (95% CI: -2.1 to 2.9); p=0.77   **EQ-5D descriptive score:**   - At extended follow-up: DF of 0.040 (95% CI: 0.005 to 0.075); p=0.03 - At 12 months: DF of -0.025 (95% CI: -0.050 to 0.000); p=0.05   **EQ-5D visual analogue scale score:**   - At extended follow-up: DF of -0.8 (95% CI: -3.2 to 1.7); p=0.056 - At 12 months: DF of -1.3 (95% CI: -3.2 to 0.6); p=0.018   **Comprehensive ThyPRO-39 score:** DF of -0.5 (95% CI: -2.2 to 1.3); p=NS  **Basic activities of daily living (Barthel Index):** DF of -0.1 (95% CI: -0.3 to 0.1); p=NS  **Instrumental activities of daily living (OARS):** DF of -0.1 (95% CI: -0.3 to 0.1); p=NS  **Letter Digit Coding Test:** DF of -0.1 (95% CI: -0.9 to 0.7); p=NS  **SBP:**   - At extended follow-up: DF of 1.1 mmHg (95% CI: -4.1 to 2.1); p=0.51 - At 12 months: DF of 0.1 mmHg (95% CI: -2.1 to 2.4); p=0.90   **DBP:**   - At extended follow-up: DF of 0.5 mmHg (95% CI: -1.4 to 2.4); p=0.59 - At 12 months: DF of -0.1 mmHg (95% CI: -1.5 to 1.3); p=0.93   **BMI:**   - At extended follow-up: DF of 0.2 kg/m^2^ (95% CI: -0.1 to 0.5); p=0.30 - At 12 months: DF of 0.0 kg/m^2^ (95% CI: -0.2 to 0.2); p=0.89 | **≥ 1 Serious AE:** HR of 0.94 (95% CI: 0.88-1.00); p=0.049  “The number of patients with at least one serious adverse event was slightly higher in the placebo group than in the levothyroxine group (P = 0.049)”  Stott et al. 2017 also report p=0.05 for the number of patients with ≥1 serious adverse event (Table 3 page 2543).  Personal communication with author indicated that the p-value in Table 3 should have read p<0.05, and additionally, due to a statistical program error, the correct p-value is p=0.053. |
| Zhao, 2016, China  n=378  No treatment | LT_4_ treatment (initial: 25 µg/day, titrated to achieve clinical euthyroidism),  follow-up of 15 months | **TC:** Control (-0.17 mmol/L) vs treatment (-0.41mmol/L); p=0.012  **TG:** Control (-0.11mmol/L) vs treatment (-0.17 mmol/L); p=0.406  **SBP:** Control (138.55±19.80 mmHg) vs treatment (136.01±20.13 mmHg); p=0.240  **DBP:** Control (79.56±11.23 mmHg) vs treatment (79.43±10.96 mmHg); p=0.917 | **AE or symptoms:** Control (13[8.18%]) vs treatment (16[7.62%]); p=0.844  No participant attempted to visit a physician due to adverse effects or withdrew from the trial due to adverse effects. |
| Liu, 2015, China  n=136  Placebo | LT_4_ treatment (initial: 12.5 µg/day, titrated to achieve TSH WNL)  follow-up of 48 weeks | **TC:** Control (0.22±0.28 mmol/L) vs treatment (-0.15±0.23 mmol/L); p<0.001  **LDL:** Control (0.08±0.24 mmol/L) vs treatment (-0.05±0.20 mmol/L); p=0.003  **HDL:** Control (-0.01±0.06 mmol/L) vs treatment (0.00±-0.07 mmol/L); p=0.412  **TG:** Control (0.01±0.10 mmol/L) vs treatment (-0.03±-0.16 mmol/L); p=0.132  **SBP:** Control (2±4 mmHg) vs treatment (1±4 mmHg); p=0.602  **DBP:** Control (2±3 mmHg) vs treatment (1±3 mmHg); p=0.386  **BMI:** Control (0.15±0.14 kg/m^2^) vs treatment (0.10±0.14 kg/m^2^); p=0.101 | In the treatment group, 9/60 experienced adverse events: 2 had mild insomnia during the treatment; 1 had mild diarrhea with mild symptoms lasting less than 2 days; 1 had mild paroxysmal supraventricular tachycardia at the beginning of the treatment; 5 had symptoms of palpitation after treatment. |
| Najafi, 2015, Iran  n=60  Placebo | LT_4_ treatment (100 µg/day),  follow-up of 12 weeks | **BDI:** Control (11.86±10.71) vs treatment (12.37±10.01); p≥0.05 | **NR** |
| Reuters, 2012, Brazil  n=71  Placebo | LT_4_ treatment (initial: 25-75 µg/day titrated to achieve TSH WNL),  follow-up of 6 months | **BDI:** Control (-2.1±4.8) vs treatment (-2.4±5.8); p=0.834  **Hamilton scale for anxiety:** Control (-4.1±7.6) vs treatment (-3.6±5.1); p=0.832  **Hamilton scale for depression:** Control (-0.6±2.9) vs treatment (-1.6±2.8); p=0.245  **SF-36:** Control (-0.8±1.6) vs treatment (-0.5±1.2); p=0.939 | **NR** |
| Cabral, 2011, Brazil  n=32  No treatment | LT_4_ treatment (initial: 0.75 µg/kg/day titrated to achieve TSH, FT_4_ WNL,  follow-up of 1 year | **TC:** Control (227.6±36.9 mg/dL) vs treatment (208.4±36.7 mg/dL); p≥0.05  **LDL:** Control (150.6±37.74 mg/dL) vs treatment (132.8±37.5 mg/dL); p≥0.05  **HDL:** Control (49.61±9.74 mg/dL) vs treatment (54.36±12.3 mg/dL); p≥0.05  **TG:** Control (137.0±56.06 mg/dL) vs treatment (106.0±36.7 mg/dL); p≥0.05 | **NR** |
| Parle, 2010, UK  n=94  Placebo | LT_4_ treatment (initial: 25 µg/day titrated to achieve TSH WNL),  follow-up of 12 months | **HADS:** Control (3.31(SEM=0.47)) vs treatment (3.61(SEM=0.36)); p=0.61  **TM test A:** Control (46.97(SEM=3.55)) vs treatment (44.52(SEM=2.62)); p=0.57  **TM test B:** Control (108.38(SEM=14.12)) vs treatment (96.67(SEM=9.62)); p=0.48  **TM test B-A:** Control (63.76(SEM=11.72)) vs treatment (52.15(SEM=6.53)); p=0.36  **MEAMS:** Control (11.44(SEM=0.20)) vs treatment (11.78(SEM=0.07)); p=0.08  **SCOLP**: Control (0.22(SEM=0.58)) vs treatment (1.69(SEM=0.43)); p=0.04  **MMSE:** Control (28.25(SEM=0.37)) vs treatment (28.28(SEM=0.29)); p=0.95 | 11 patients withdrew during the trial due to side effects (6 from the placebo group and 5 from the treatment group). |
| Mainenti,  2009, Brazil  n=23  No treatment | LT_4_ treatment (initial: 0.75 µg/kg/day titrated to achieve TSH WNL),  follow-up of 6 months | **SBP:** Control (154.55±23.9 mm/Hg) vs treatment (142.3±18.2 mm/Hg); p=0.17  **DBP:** Control (88.6±7.5 mm/Hg) vs treatment (83.2±6.4 mm/Hg); p=0.28 | **NR** |
| Nagasaki, 2009, Japan  n=95  Placebo | LT_4_ treatment (initial: 12.5 µg/day titrated to achieve TSH WNL),  follow-up of 5 months | **TC:** Control (5.33±SEM 0.24 mmol/L) vs treatment (5.19±SEM 0.16 mmol/L); p=0.50  **LDL:** Control (3.36±SEM 0.19 mmol/L) vs treatment (3.14±SEM 0.29 mmol/L); p=0.38  **HDL:** Control (1.39±SEM 0.06 mmol/L) vs treatment (1.41±SEM 0.08 mmol/L); p=0.79  **TG:** Control (1.38±SEM 0.14 mmol/L) vs treatment (1.50±SEM 0.16 mmol/L); p=0.43  **SBP:** Control (132.2±SEM 3.5 mmHg) vs treatment (128.8±SEM 3.8 mmHg); p=0.50  **DBP:** Control (72.8±SEM 2.0 mmHg) vs treatment (72.7±SEM 2.2 mmHg); p=0.05  **BMI:** Control (22.1±SEM 0.05 kg/m^2^) vs treatment (21.8±SEM 0.48 kg/m^2^); p=0.55 | None of the patients experienced side effects such as arrhythmia, angina pectoris, or hypertension that would have required withdrawal or reduction of the dose of levothyroxine. |
| Mikhail, 2008, Kuwait  n=120  Placebo | LT_4_ treatment (initial: 25 µg/day titrated to achieve TSH WNL),  follow-up of 52 weeks | **TC:** Control (5.0468±SEM 0.6723 mmol/L) vs treatment (4.7427± SEM 0.8731 mmol/L); p<0.029  **LDL:** Control (3.1106± SEM 0.7716 mmol/L) vs treatment (2.8957± SEM 0.5946 mmol/L); p<0.0001  **HDL:** Control (1.1035± SEM 0.2502 mmol/L) vs treatment (1.1957± SEM 0.3228 mmol/L); p=0.084  **TG:** Control (1.0642± SEM 0.5946 mmol/L) vs treatment (0.9538± SEM 0.5354 mmol/L); p=0.295 | **NR** |
| Teixeira, 2008, Brazil  n=60  Placebo | LT_4_ treatment (25-75 µg/day titrated to achieve TSH, FT_4_ WNL)  follow-up of 6 and 12 months | **TC:**   - At 12 months: Control (202.7±40.5 mg/dL) vs treatment (197.0±28.7 mg/dL); p=0.032 - At 6 months: Control (188.3±48.2 mg/dL) vs treatment (214.1±46.9 mg/dL); p=0.070   **LDL:**   - At 12 months: Control (129.7±35.2 mg/dL) vs treatment (118.3±24.2 mg/dL); p=0.024 - At 6 months: Control (118.1±51.8 mg/dL) vs treatment (138.2±44.7 mg/dL); p=0.119   **HDL:**   - At 12 months: Control (48.5±9.9 mg/dL) vs treatment (54.8±17.5 mg/dL); p=0.180 - At 6 months: Control (55.1±15.0 mg/dL) vs treatment (55.6±9.8 mg/dL); p=0.0965   **TG:**   - At 12 months: Control (122.7±58.5 mg/dL) vs treatment (105.0±58.7 mg/dL); p=0.384 - At 6 months: Control (98.1±38.5 mg/dL) vs treatment (102.3±50.8 mg/dL); p=0.988   **BMI:**   - At 12 months: Control (24.5±3.3 kg/m^2^) vs treatment (27.4±3.2 kg/m^2^); p=0.02 - At 6 months: Control (23.5±3.1 kg/m^2^) vs treatment (26.7±1.9 kg/m^2^); p=0.001 | One patient in the treatment group developed hashitoxicosis and 1 patient had symptomatic tachycardia. |
| Duman, 2007, Turkey  n=63  No treatment | LT_4_ treatment (initial: 25 µg/day, titrated to achieve euthyroidism),  follow-up of 8 months | **TC:** Control (202±28 mg/dL) vs treatment (202±28 mg/dL); p=1.00  **LDL:** Control (128±25 mg/dL) vs treatment (130±32 mg/dL); p=0,84  **HDL:** Control (53.08±8.6 mg/dL) vs treatment (53±16 mg/dL); p=1.00  **TG:** Control (128±58 mg/dL) vs treatment (93±37 mg/dL); p=0.02  **BMI:** Control (25.5±3.8 kg/m^2^) vs treatment (24.8±3.2 kg/m^2^); p=0.55 | **NR** |
| Fadeyev, 2006, Russia  n=33  No treatment | LT_4_ treatment (initial: 25 µg/day, titrated to achieve TSH WNL)  follow-up of 6 months | N/A | In the treatment group, 5/19 patients had various adverse events during treatment : 3 had more ventricular premature beats (VPB) and 2 had an increased mean heart rate in conjunction with an increased number of VPB.  At the end of the follow-up period, one of the patients in the treatment group had an unstable episode of ventricular tachycardia. |
| Iqbal, 2006, Norway  n=64  Placebo | LT_4_ treatment (initial: 50 µg/day, titrated to achieve TSH WNL),  follow-up of 12 months | **TC:** Control (5.8±0.9 mmol/L) vs treatment (5.7±1.1 mmol/L); p=0.70  **LDL:** Control (3.6±1.0 mmol/L) vs treatment (3.6±0.9 mmol/L); p=1.00  **HDL:** Control (1.5±0.5 mmol/L) vs treatment (1.5±0.4 mmol/L); p=1.00  **TG:** Control (1.6±0.7 mmol/L) vs treatment (1.5±1.0 mmol/L); p=0.65  **BMI:** Control (27.0±4.1 kg/m^2^) vs treatment (28.4±5.8 kg/m^2^); p=0.27 | **NR** |
| Jorde, 2006, Norway  n=70  Placebo | T_4_ tablets (in 25-, 50-, and 100- µg strength) (set doses in first 12 weeks followed by individual dose adjustments thereafter to achieve a TSH between 0.5-1.5 mIU/L),  follow-up of 12 months | **BDI:** Control (3.3±4.0) vs treatment (4.3±3.6); p≥0.05^6^  **GHQ-30:** Control (1.2±2.0) vs treatment (1.9±3.3); p≥0.05^6^  **Composite cognitive score:** Control (-0.9±4.8) vs treatment (1.5±3.7); p≥0.05^6^  **TM test A:** Control (44.1±17.7) vs treatment (39.0±14.8); p≥0.05^6^  **TM test B:** Control (103±49) vs treatment (94±62); p≥0.05^6^  **Seashore Rhythm test:** Control (409±114) vs treatment (372±108); p≥0.05^6^  **Word List test:** Control (25.2±9.5) vs treatment (26.3±5.6); p≥0.05^6^  **Controlled Word Association test:** Control (41.6±14.1) vs treatment (41.7±15.0); p≥0.05^6^  **CalCAP:** Control (1562±331) vs treatment (1483±302); p≥0.05^6^  **Vocabulary (WAIS):** Control (19.7±4.2) vs treatment (19.8±3.5); p≥0.05^6^ | **NR** |
| Caraccio, 2005, Italy  n=23  Placebo | LT_4_ treatment (initial: 25 µg/day, titrated to achieve TSH WNL),  follow-up of 6 months | **BMI:** Control (22.7± SEM 0.6 kg/m^2^) vs treatment (22.3± SEM 0.9 kg/m^2^); p=NS | **NR** |
| Yazici, 2004, Turkey  n=45  Placebo | LT_4_ treatment (initial: 50 µg/day, titrated to achieve TSH WNL),  follow-up of 1 year | **SBP:** Control (123.1±9.8 mm/Hg) vs treatment (123.6±9.8 mm/Hg); p≥0.05  **DBP:** Control (77.2±8.4 mm/Hg) vs treatment (76.7±8.9 mm/Hg); p≥0.05  **BMI:** Control (23.0±3.1 kg/m^2^) vs treatment (22.8±3.4 kg/m^2^); p≥0.05 | **NR** |
| Monzani, 2004, Italy  n=45  Placebo | LT_4_ treatment (initial: 25 µg/day, titrated to achieve TSH WNL),  follow-up of 6 months | **TC:** Control (219.6±48.9 mg/dL) vs treatment (191.6±32.5 mg/dL); p=0.03  **LDL:** Control (141.3±38.6 mg/dL) vs treatment (119.2±27.8 mg/dL); p=0.03  **HDL:** Control (57.8±11.6 mg/dL) vs treatment (54.7±7.4 mg/dL); p≥0.05  **TG:** Control (102.7±53.1 mg/dL) vs treatment (88.1±30.0 mg/dL); p≥0.05  **SBP:** Control (114±13 mmHg) vs treatment (112±15 mmHg); p≥0.05  **DBP:** Control (72±8 mmHg) vs treatment (69±9 mmHg); p≥0.05  **BMI:** Control (24.9±3.8 kg/m^2^) vs treatment (23.7±3.5 kg/m^2^); p≥0.05 | **NR** |
| Caraccio, 2002, Italy  n=49  Placebo | LT_4_ treatment (initial: 25 µg/day, titrated to achieve TSH WNL),  follow-up of 6 months | **TC:** Control (5.3±1.1 mmol/L) vs treatment (5.0±1.1 mmol/L); p=NS  **LDL:** Control (3.4±0.9 mmol/L) vs treatment (3.1±1.0 mmol/L); p=NS  **HDL:** Control (1.5±0.3 mmol/L) vs treatment (1.4±0.3 mmol/L); p=NS  **TG:** Control (1.3±0.7 mmol/L) vs treatment (1.2±1.6 mmol/L); p=NS  **BMI:** Control (22.8±2.7 kg/m^2^) vs treatment (24.1±3.1 kg/m^2^); p=NS | **NR** |
| Monzani, 2001, Italy  n=20  Placebo | LT_4_ treatment (initial: 50 µg/day, titrated to achieve TSH WNL),  follow-up of 1 year | **SBP:** Control (116.8±9.6 mmHg) vs treatment (117.3±6.8 mmHg); p≥0.05  **DBP:** Control (71.0±7.0 mmHg) vs treatment (74.8±7.2 mmHg); p≥0.05  **BMI:** Control (23.1±2.2 kg/m^2^) vs treatment (23.1±4.7 kg/m^2^); p≥0.05 | **NR** |
| Andersen,  2016, Denmark  n=1,192  Untreated | Treated: those who initiated LT_4_ during follow-up,  Observation period varied (between 1997-2011) | **All-cause mortality:**  By sex (Females): adjusted IRR of 1.08 (95% CI: 0.80-1.48); p≥0.05  By sex (Males): adjusted IRR of 1.43 (95% CI: 0.87-2.34); p≥0.05  **MACE:**  By sex (Females): adjusted IRR of 0.99 (95% CI: 0.70-1.40); p≥0.05  By sex (Males): adjusted IRR of 1.36 (95% CI: 0.79-2.35); p≥0.05 | **NR** |
| Andersen,  2015, Denmark  n=12,212  Untreated | Treated: those who initiated LT_4_ ≤ 6 months from the date of first TFT  Observation period varied (between 2000-2009) | **All-cause mortality:**  By age (<65 years): adjusted IRR of 0.63 (95% CI: 0.40-0.99); p<0.05  By sex (Females): adjusted IRR of 0.99 (95% CI: 0.85-1.16); p≥0.05  By sex (Males): adjusted IRR of 1.24 (95% CI: 0.89-1.71); p≥0.05  **Deaths due to CVD:**  By age (<65 years): adjusted IRR of 0.55 (95% CI: 0.25-1.20); p≥0.05  By sex (Females): adjusted IRR of 0.96 (95% CI: 0.77-1.21); p≥0.05  By sex (Males): adjusted IRR of 1.32 (95% CI: 0.83-2.08); p≥0.05  **MI:**  By age (<65 years): adjusted IRR of 1.11 (95% CI: 0.61-2.02); p≥0.05  By sex (Females): adjusted IRR of 0.98 (95% CI: 0.70-1.38); p≥0.05  By sex (Males): adjusted IRR of 1.41 (95% CI: 0.83-2.40); p≥0.05 | **NR** |
| Razvi, 2012, UK  n=4,735  Untreated | Treated: those who initiated LT4 during follow-up  Observation period varied (between 2000-2009) | **All-cause mortality:**  By age (40-70 years): multivariate adjusted HR of 0.36 (95% CI: 0.19-0.66); p<0.0001  **Deaths due to circulatory diseases:**  By age (40-70 years): multivariate adjusted HR of 0.54 (95% CI: 0.37-0.92); p=0.01  **Fatal or non-fatal cerebrovascular disease:**  By age (40-70 years): multivariate adjusted HR of 1.03 (95% CI: 0.51-2.13); p=0.94  **Atrial Fibrillation:**  By age (40-70 years): multivariate adjusted HR of 0.76 (95% CI: 0.26-1.73); p0.58  **Fatal or non-fatal ischemic heart disease events:**  By age (40-70 years): multivariate adjusted HR of 0.61 (95% CI: 0.39-0.95); p=0.03 | **NR** |

**AE**=adverse event; **BDI**=Beck Depression Inventory; **BMI**=body mass index; **CalCAP**=California Computerized Assessment Package; **CV**=cardiovascular; **CVD**=cardiovascular diseases; **DF**=difference; **DBP**=diastolic blood pressure; **EQ-5D**=Euroquol group 5-dimension self-report questionnaire; **GHQ-30**=General Health Questionnaire; **HADS**=Hospital anxiety and depression scale; **HDL**=high-density lipoprotein; **HR**=hazard ratio; **IHD**=ischemic heart disease; **IRR**=incidence rate ratio; **LDL**=low-density lipoprotein; **LT_4_**=levothyroxine; **MACE**=Major adverse cardiac event; **MEAMS**=Middlesex Elderly Assessment of Mental State; **MI**=myocardial infarction; **MMSE**=Mini-Mental State Examination; **N** =number of participants; **N/A**=not applicable; **NR**=not reported; **NS**=not significant; **OARS**=Older American Resources and Services tool; **P**=p-value; **SBP**=systolic blood pressure; **SCH**=subclinical hypothyroidism; **SCOLP**=Speed and Capacity of Language Processing test; **SE**=standard error; **SF-36**=Medical outcomes study 36-item short-form health survey; **T_4_**=thyroxine; **TFT**=thyroid function test; **TC**=total cholesterol; **TG**=triglyceride; **ThyPRO**=Thyroid-Related Quality-of-Life Patient-Reported Outcome; **TM**=Trail Making test; **TSH**=thyroid-stimulating hormone; **UK**=United Kingdom; **WAIS**= Wechsler Intelligence Scales; **WNL**=within normal limits

# Appendix 9. Risk of Bias Assessments

**Risk of bias assessments – Randomized controlled trials** (using the Cochrane Risk of Bias Tool)

| **Author** | **Sequence Generation** | **Allocation Concealment** | **Blinding of Participants and Personnel** | **Blinding of Outcome Assessment** | **Incomplete Outcome Data** | **Selective Reporting** | **Other Bias** |
| --- | --- | --- | --- | --- | --- | --- | --- |
| Cabral 2011 | - | + | - | - | ? | ? | ? |
| Caraccio 2002 | ? | ? | - | - | ? | ? | ? |
| Caraccio 2005 | ? | ? | - | - | - | ? | ? |
| Duman 2007 | ? | ? | - | - | - | ? | ? |
| Fadeyav 2006 | - | + | + | ? | ? | ? | + |
| Iqbal 2006 | ? | ? | - | - | - | - | ? |
| Jorde 2006 | ? | ? | - | - | ? | - | ? |
| Liu 2015 | - | ? | - | ? | ? | ? | ? |
| Mainenti 2009 | ? | ? | + | ? | ? | ? | ? |
| Mikhail 2008 | ? | ? | - | - | ? | ? | ? |
| Monzani 2001 | ? | ? | - | ? | ? | ? | ? |
| Monzani 2004 | ? | ? | - | - | ? | ? | ? |
| Nagasaki 2009 | ? | ? | - | - | ? | ? | ? |
| Najafi 2015 | + | ? | ? | ? | ? | ? | ? |
| Parle 2010 | - | ? | - | - | + | ? | + |
| Reuters 2012 | ? | ? | - | ? | + | ? | ? |
| Stott 2017 | - | - | - | - | - | - | - |
| Teixeira 2008a | ? | ? | - | - | + | ? | ? |
| Teixeira 2008b | ? | ? | - | - | + | ? | + |
| Yazici 2004 | ? | ? | - | ? | ? | ? | ? |
| Zhao 2016 | - | ? | + | ? | - | ? | ? |

**Risk of bias assessments – Cohort studies** (using the Newcastle-Ottawa Scale for Cohort Studies)

| **Category** | **Andersen 2015** | **Andersen 2017** | **Razvi 2012** |
| --- | --- | --- | --- |
| **Selection** |  |  |  |
| Representativeness of the exposed cohort | * | * | * |
| Selection of the non-exposed cohort | * | * | * |
| Ascertainment of exposure | * | * | * |
| Demonstration that outcome of interest was not present at start of study | * | * | * |
| **Comparability** – Comparability of cohorts on the basis of design or analysis |  |  |  |
| Study controls for an important factor | * | * | * |
| Study controls for an additional factor | * | * | * |
| **Outcome** |  |  |  |
| Assessment of outcome | * | * | * |
| Was follow-up long enough for outcomes to occur |  |  |  |
| Adequacy of follow-up of cohorts | * | * | * |
| **Total number of stars (out of 9):** | **8** | **8** | **8** |

# Appendix 10. Measures of Quality of Life and Cognitive Function

**Quality of Life**

**36-Item Short Form Health Survey (SF-36)** [^[[1]](#endnote-1)^] – a quality of life survey that looks at eight health concepts: physical functioning, bodily pain, role limitations due to physical health problems, role limitations due to personal or emotional problems, emotional well-being, social functioning, energy/fatigue, and general health perceptions.

**Beck Depression Inventory (BDI)** [^[[2]](#endnote-2)^] - is a 21-item, self-report rating inventory that measures characteristic attitudes and symptoms of depression and includes the following subscales: (a) Affective subscale to measure affective components (i.e. pessimism, past failures, guilty feelings, punishment feelings, self-dislike, self-criticalness, suicidal thoughts or wishes, and worthlessness) and (b) Somatic subscale to measure somatic components (i.e. sadness, loss of pleasure, crying, agitation, loss of interest, indecisiveness, loss of energy, change in sleep patterns, irritability, change in appetite, concentration difficulties, tiredness and/or fatigue, and loss of interest in sex)

**Barthel index** [^[[3]](#endnote-3)^] – measures the extent to which somebody can function independently and has mobility in their activities of daily living (i.e. feeding, bathing, grooming, dressing, etc.).

**Comprehensive Thyroid Quality of Life Assessment ThyPRO-39 score** [^[[4]](#endnote-4)^] – an abbreviated version of the Thyroid-related Quality of Life Patient-reported outcome measure (ThyPRO)

**EuroQuol Group 5-Dimension Self Report Questionnaire (EQ-5D)** [^[[5]](#endnote-5)^] – standardized instrument for measuring generic health status and consists of two components. The first is a descriptive score which comprises five dimensions (mobility, self-care, usual activities, pain/discomfort and anxiety/depression) and the second is a visual analogue score to measure current health status.

**General Health Questionnaire (GHQ-30)** [^[[6]](#endnote-6)^] - is a screening device for identifying minor psychiatric disorders in the general population and within community or non-psychiatric clinical settings such as primary care or general medical out-patients

**Hamilton Scale for Anxiety and Depression** [^[[7]](#endnote-7)^,^[[8]](#endnote-8)^] - a 14 item scale that determine the levels of anxiety and depression that a patient is experiencing and includes two subscales: anxiety scale and depression scale

**Hospital Anxiety and Depression Scale (HADS)** [^[[9]](#endnote-9)^] – measures anxiety and depression in a general medical population of patients

**Older American Resources and Services (OARS) Instrumental Activities of Daily Living** [^[[10]](#endnote-10)^] - The Activities of Daily Living (ADL) questionnaire is a component of the OARS Multidimensional Functional Assessment Questionnaire. It measures an individual’s functioning related to independent living and one’s interaction with one’s physical and social environment

**Thyroid-related Quality of Life Patient-reported outcome measure (ThyPRO)** [^[[11]](#endnote-11)^] - a questionnaire for measurement of quality of life in patients with benign thyroid diseases. Includes the following sub-measures: Hypothyroid Symptoms Score Hypothyroid Tiredness Score.

**Cognitive Function**

**California Computerized Assessment Package (CalCAP)** [^[[12]](#endnote-12)^] – assesses speed of information processing

**Composite Cognitive Score** [^[[13]](#endnote-13)^] – Used in one study and was made by adding together the Z-scores for the following tests: Digit span forward and backward (to assess attention and working memory), Stroop tests parts 1 & 2 (psychomotor/cognitive speed), Verbal and visual recall (memory), and the Stroop test part 3(cognitive flexibility/executive function)

**Controlled Word Association test** [^[[14]](#endnote-14)^] – assesses language/word fluency

**Letter Digit Coding Test** [^[[15]](#endnote-15)^] – to assess executive cognitive function whereby the test would indicate the speed of processing

**Middlesex Elderly Assessment of Mental State (MEAMS)** [^[[16]](#endnote-16)^] – screens for gross impairment of cognitive skills in the elderly

**Mini-Mental State Examination (MMSE)** [^[[17]](#endnote-17)^] – measures cognitive impairment

**Seashore Rhythm test** [^[[18]](#endnote-18)^] – assesses attention and working memory

**Speed and Capacity of Language Processing test (SCOLP)** [^[[19]](#endnote-19)^] - measures the slowing in cognitive processes

**Trail Making Test** [^[[20]](#endnote-20)^] – is a neuropsychological test of visual attention and task switching. Test A assesses psychomotor/cognitive speed and Test B assesses cognitive flexibility/executive function. Trail making test B-A, or the difference in score, can be used to control for the effect of motor speed on performance to give a more accurate measure of executive function than the performance on either part alone.

**Wechsler Intelligence Scales (WAIS) Vocabulary scale** [^[[21]](#endnote-21)^] – assesses intelligence, verbal knowledge and concept formation

**Word List test** [^[[22]](#endnote-22)^] – assesses memory and is a sub-test from the California Verbal Learning Test

1. [] Montazeri, Ali, et al. "The Short Form Health Survey (SF-36): translation and validation study of the Iranian version." *Quality of life research* 14.3 (2005): 875-882. [↑](#endnote-ref-1)
2. [] Beck, Aaron T., Robert A. Steer, and Gregory K. Brown. "Beck depression inventory-II." *San Antonio* 78.2 (1996): 490-8. [↑](#endnote-ref-2)
3. [] Collin, C., et al. "The Barthel ADL Index: a reliability study." *International disability studies* 10.2 (1988): 61-63. [↑](#endnote-ref-3)
4. [] Watt Torquil, Bjorner Jakob Bue, Groenvold Mogens, Cramon Per, Winther Kristian Hillert, Hegedüs Laszlo, Bonnema Steen Joop, Rasmussen Åse Krogh, Ware John E. Jr., and Feldt-Rasmussen Ulla. Thyroid. September 2015, 25(10): 1069-1079.  [↑](#endnote-ref-4)
5. [] The EuroQol Group (1990). EuroQol-a new facility for the measurement of health-related quality of life. Health Policy 16(3):199-208. [↑](#endnote-ref-5)
6. [] Goldberg D, Williams P. A user’s guide to the general health questionnaire. Windsor: NFER-Nelson, 1988 [↑](#endnote-ref-6)
7. [] Euan Thompson; Hamilton Rating Scale for Anxiety (HAM-A), Occupational Medicine, Volume 65, Issue 7, 1 October 2015, Pages 601 [↑](#endnote-ref-7)
8. [] Rachel Sharp; The Hamilton Rating Scale for Depression, Occupational Medicine, Volume 65, Issue 4, 1 June 2015, Pages 340 [↑](#endnote-ref-8)
9. [] Snaith, R. Philip. "The hospital anxiety and depression scale." *Health and quality of life outcomes* 1.1 (2003): 29. [↑](#endnote-ref-9)
10. [] Doble, S. E., and A. G. Fisher. "The dimensionality and validity of the Older Americans Resources and Services (OARS) Activities of Daily Living (ADL) scale." *Journal of Outcome Measurement* 2.1 (1998): 4-24. [↑](#endnote-ref-10)
11. [] Watt, Torquil, et al. "The thyroid-related quality of life measure ThyPRO has good responsiveness and ability to detect relevant treatment effects." The Journal of Clinical Endocrinology & Metabolism 99.10 (2014): 3708-3717. [↑](#endnote-ref-11)
12. [] Miller, Eric N. "California computerized assessment package." Encyclopedia of Clinical Neuropsychology. Springer New York, 2011. 473-475. [↑](#endnote-ref-12)
13. [] Jorde R, Waterloo K, Storhaug H, Nyrnes A, Sundsfjord J, Jenssen TG. Neuropsychological function and symptoms in subjects with subclinical hypothyroidism and the effect of thyroxine treatment. J Clin Endocrinol Metab. 2006;91(1):145-53. [↑](#endnote-ref-13)
14. [] Spreen O, Strauss E 1998 A compendium of neuropsychological tests. 2nd ed. New York: Oxford University Press [↑](#endnote-ref-14)
15. [] Houx PJ, Shepherd J, Blauw GJ, et al. Testing cognitive function in elderly populations: the PROSPER study: PROspective Study of Pravastatin in the Elderly at Risk.J Neurol Neurosurg Psychiatry 2002; 73: 385-9. [↑](#endnote-ref-15)
16. [] Golding, E. "Middlesex Elderly Assessment of Mental State (MEAMS)." Thames Valley Test Company, Bury St. Edmunds (1989). [↑](#endnote-ref-16)
17. [] Folstein, Marshal F., Susan E. Folstein, and Paul R. McHugh. "“Mini-mental state”: a practical method for grading the cognitive state of patients for the clinician." Journal of psychiatric research 12.3 (1975): 189-198. [↑](#endnote-ref-17)
18. [] Meyers, John E. "Seashore Rhythm Test." *Encyclopedia of Clinical Neuropsychology*. Springer New York, 2011. 2219-2219. [↑](#endnote-ref-18)
19. [] Baddeley, A., Emslie, H., & Smith, I. N. (1992). The Speed and Capacity of Language-Processing Test. Bury St. Edmunds, England: Thames Valley Test Company [↑](#endnote-ref-19)
20. [] Reitan RM, Wolfson D 1993 The Halstead-Reitan Neuropsychological Test Battery. 2nd ed. Tucson, AZ: Neuropsychology Press [↑](#endnote-ref-20)
21. [] Wechsler D 1987 Wechsler Memory Scale-Revised. Manual. New York: The Psychological Corp. [↑](#endnote-ref-21)
22. [] Delis DC, Kramer JH, Kaplan E, Ober BA 1987 California Verbal Learning Test: Adult Version. San Antonio, TX: The Psychological Corp.

    # Appendix 11. Author Correspondence

    | Study Author | Date of Contact | Information Sought | Response |
    | --- | --- | --- | --- |
    | Jorde 2006 | 2018 10 15 | Is composite cognitive score adjusted for baseline? | Yes |
    | Mikhail 2008 | 2018 10 01 (via Embassy of Kuwait) | Lipid SEM appears to be SD | Could not locate contact information. |
    | Stott 2017 | 2018 12 04 | Attrition at extended follow-up | Pending |
    |  | 2018 10 30 | Adverse Event P-value clarification | Error in stats program. New p-value provided. |
    |  | 2017 09 06 | Definition for what constitutes a "serious adverse event" in the TRUST trail. | We used the legal definition of SAEs as required by our regulatory authorities. |
    | Andersen 2015/2016 | 2018 10 02  2018 10 18 | The total number of participants who were aged < 65 years? And of these, how many were “treated” and “untreated”? | No response |
    | Monzani 2004 | 2017 06 26 | Are the patients from the 2 studies above the same? Or were these 2 separate studies for which there were 2 separate patient populations used? | The data of the two studies belonged to 2 different databases obviously, some patients were registered in both the databases. |
    | Zhao 2016 | 2019 06 10 | Mean, standard deviation, and p-value for the between group comparison of Mild SCH-Control to Mild SCH-Intervention for: 1) Body Mass Index, 2) Weight, 5) HDL, and 6) LDL. | Auto-acknowledgement of email receipt; no data provided. |

    [↑](#endnote-ref-22)
