# Supplementary material for: Screening for thyroid dysfunction and treatment of screen-detected thyroid dysfunction in asymptomatic, community-dwelling adults: a systematic review
Source: Syst Rev. 2019 Nov 18;8:260. doi: 10.1186/s13643-019-1181-7 (PMC6859607; doi:10.1186/s13643-019-1181-7)
Supplement: Supplementary file 2 — Additional file 2: Evidence Sets 1-3 contain GRADE Summary of Findings Tables and GRADE Evidence Profile Tables for KQ3a, KQ3b, and KQ4. [file 13643_2019_1181_MOESM2_ESM.docx]

**Evidence Set 1**

**KQ3a: Does treatment of screen-detected overt or subclinical thyroid dysfunction improve morbidity or mortality?**

**Table 1.1 GRADE Summary of Findings Table – morbidity and mortality outcomes**

| **3a. Does treatment of screen-detected overt or subclinical thyroid dysfunction improve morbidity or mortality?** | | | | | |  |
| --- | --- | --- | --- | --- | --- | --- |
| **Outcomes.**  **Follow-up** | **№ of participants (№ of studies)** | **Certainty of the evidence (GRADE)** | **Relative effect (95% CI)** | **Anticipated absolute effects** | | **Comments** |
|  |  |  |  | **Risk with no treatment or placebo** | **Risk difference with treatment** |  |
| All-cause mortality - RCT follow up: range over 12 months to 3 years | 737 (1 RCT) ^a^ | ⨁⨁◯◯ LOW ^b,c,d,e^ | **HR 1.91** (0.65 to 5.60) | 14 per 1,000 | **12 more per 1,000** (5 fewer to 60 more) | There may be no statistically significant difference between those treated and not treated for subclinical hypothyroidism on all-cause mortality. |
| All-cause mortality by AGE ((Adults (<65 years or 40-70 years)) - Cohort  follow-up: median of 5.0-7.6 years | Range: 3,093 to 12,212^[[1]](#footnote-1)^ per study  (2 OBS studies) ^f^ | ⨁◯◯◯ VERY LOW ^g,h,i,j^ | Both studies showed a beneficial effect of treatment for subclinical hypothyroidism in adults <65 years or between 40-70 years of age for all-cause mortality. Andersen 2015 reported an adjusted IRR of 0.63 (95% CI: 0.40-0.99) and Razvi 2012 reported a multivariate adjusted HR of 0.36 (95% CI: 0.19-0.66) [absolute value of 41 fewer deaths per 1,000, ranging from 52 fewer to 21 fewer). | | | There is large uncertainty over a potential beneficial effect of treatment for subclinical hypothyroidism among younger adults. |
| All-cause mortality by AGE (Adults(>65 years)) - RCT follow up: range over 12 months to 3 years | 737 (1 RCT) ^a^ | ⨁⨁⨁◯ MODERATE ^b,c,e,i^ | **HR 1.91** (0.65 to 5.60) | 14 per 1,000 | **12 more per 1,000** (5 fewer to 60 more) | There is moderate certainty that no statistically significant difference exists between older adults treated and not treated for subclinical hypothyroidism on all-cause mortality. |
| All-cause mortality by SEX (Females) - Cohort  follow-up: median of 5.0-5.6 years | Range: 760^[[2]](#footnote-2)^ to 9,743^ii^ per study  (2 OBS studies) ^k^ | ⨁◯◯◯ VERY LOW ^g,i,l,m^ | Both studies found no benefit of treatment in females for subclinical hypothyroidism on all-cause mortality. Andersen 2015 reported an adjusted IRR of 0.99 (95% CI: 0.85-1.16) and Andersen 2016 reported an adjusted IRR of 1.08 (95% CI: 0.80-1.48). | | | There is large uncertainty over the effects of treatment for subclinical hypothyroidism on all-cause mortality in females. |
| All-cause mortality by SEX (Males) - Cohort  follow-up: median of 5.0-5.6 years | Range: 432^ii^ to 2,469^ii^ per study  (2 OBS studies) ^k^ | ⨁◯◯◯ VERY LOW ^g,i,m,n^ | Both studies found no benefit of treatment for subclinical hypothyroidism in males on all-cause mortality. Andersen 2015 reported an adjusted IRR of 1.24 (95% CI: 0.89-1.16) and Andersen 2016 reported an adjusted IRR of 1.43 (95% CI: 0.87-2.34). | | | There is large uncertainty over the effects of treatment for subclinical hypothyroidism on all-cause mortality in males. |
| Deaths due to cardiovascular diseases - RCT follow up: range over 12 months to 3 years | 737 (1 RCT) ^a^ | ⨁◯◯◯ VERY LOW ^b,c,d,o^ | **OR 2.01** (0.18 to 22.27) | 3 per 1,000 | **3 more per 1,000** (2 fewer to 54 more) | There is large uncertainty over the effects of treatment for subclinical hypothyroidism on deaths due to cardiovascular diseases. |
| Deaths due to cardiovascular diseases by AGE (Adults (<65 years or 40-70 years)) - Cohort  follow-up: median of 5.0-7.6 years | Range: 3,093 to 12,212^i^ per study  (2 OBS studies) ^f^ | ⨁◯◯◯ VERY LOW ^g,i,m,p^ | Razvi 2012 showed a beneficial effect of treatment for subclinical hypothyroidism in patients between 40-70 years on deaths due to circulatory diseases only (multivariate adjusted HR of 0.54 (95% CI: 0.37-0.92); absolute value of 12 fewer deaths per 1,000 (ranged from 16 fewer to 2 fewer)). However, Andersen 2015 found no beneficial effect of treatment on cardiovascular deaths (adjusted IRR of 0.55 (95% CI: 0.25-1.20)). | | | There is large uncertainty over the effects of treatment for subclinical hypothyroidism on deaths due to cardiovascular diseases in younger adults |
| Deaths due to cardiovascular diseases by AGE (Adults (>65 years)) - RCT follow up: range over 12 months to 3 years | 737 (1 RCT) ^a^ | ⨁⨁◯◯ LOW ^c,g,i,o^ | **OR 2.01** (0.18 to 22.27) | 3 per 1,000 | **3 more per 1,000** (2 fewer to 54 more) | There may be no statistically significant difference between those treated and not treated for subclinical hypothyroidism on deaths due to cardiovascular diseases among older adults |
| Deaths due to cardiovascular diseases by SEX (Females) – Cohort  follow-up: median of 5.0 years | 9,743^ii^  (1 OBS study) ^q^ | ⨁◯◯◯ VERY LOW ^b,c,i,m^ | There were no differences found in females treated and not treated for subclinical hypothyroidism on cardiovascular-related deaths (adjusted IRR of 0.96, 95% CI: 0.77-1.12). | | | There is large uncertainty over the effects of treatment for subclinical hypothyroidism on deaths due to cardiovascular diseases in females. |
| Deaths due to cardiovascular diseases by SEX (Males) - Cohort  follow-up: median of 5.0 years | 2,469^ii^  (1 OBS study) ^q^ | ⨁◯◯◯ VERY LOW ^b,c,i,m^ | There were no differences found in males treated and not treated for subclinical hypothyroidism on cardiovascular-related deaths (adjusted IRR of 1.32, 95% CI: 0.83-2.08). | | | There is large uncertainty over the effects of treatment for subclinical hypothyroidism on deaths due to cardiovascular diseases in males. |
| Fatal and non-fatal cardiovascular events (not including atrial fibrillation) - RCT follow up: range over 12 months to 3 years | 737 (1 RCT) ^a^ | ⨁⨁◯◯ LOW ^b,c,d,e^ | **HR 0.89** (0.47 to 1.69) | 54 per 1,000 | **6 fewer per 1,000** (28 fewer to 36 more) | There may be no statistically significant difference between those treated and not treated for subclinical hypothyroidism on the occurrence of fatal and non-fatal cardiovascular events. |
| Fatal and non-fatal cardiovascular events (not including atrial fibrillation) - by AGE (Adults (<65 years or 40-70 years)) - Cohort  follow-up: median of 5.0-7.6 years | Range: 3,093 to 12,212^i^ per study  (2 OBS studies) ^f^ | ⨁◯◯◯ VERY LOW ^g,i,r,s^ | Razvi 2012 showed a beneficial effect of treatment for subclinical hypothyroidism in patients between 40-70 years on the occurrence of fatal and non-fatal ischemic heart disease events (multivariate adjusted HR of 0.61, 95% CI: 0.39-0.95; absolute value of 25 fewer per 1,000 (ranged from 3 fewer to 40 fewer)) but not on the occurrence of fatal and non-fatal cerebrovascular disease events (multivariate adjusted HR of 1.03, 95% CI: 0.51-2.13; absolute value of 1 more per 1,000 (ranged from 15 fewer to 27 more)). Andersen 2015 found no benefit of treatment on the occurrence of myocardial infarction (adjusted IRR of 1.11, 95% CI: 0.61-2.02). | | | There is large uncertainty over the effects of treatment for subclinical hypothyroidism on the occurrence of fatal and non-fatal cardiovascular events in younger adults. |
| Fatal and non-fatal cardiovascular events (not including atrial fibrillation) - by AGE (Adults (>65 years)) - RCT follow up: range over 12 months to 3 years | 737 (1 RCT) ^a^ | ⨁⨁⨁◯ MODERATE ^b,c,e,i^ | **HR 0.89** (0.47 to 1.69) | 54 per 1,000 | **6 fewer per 1,000** (28 fewer to 36 more) | There is moderate certainty that no statistically significant difference exists between older adults treated and not treated for subclinical hypothyroidism on the occurrence of fatal and non-fatal cardiovascular events. |
| Fatal and non-fatal cardiovascular events (not including atrial fibrillation) - by SEX (Females) - Cohort  follow-up: median of 5.0-5.6 years | Range: 760^ii^ to 9,743^ii^ per study  (2 OBS studies) ^k^ | ⨁◯◯◯ VERY LOW ^g,i,m,t^ | Both studies found no benefit of treatment for subclinical hypothyroidism in females on the occurrence of fatal and non-fatal cardiovascular events. Andersen 2015 reported an adjusted IRR of 0.99 (95% CI: 0.70-1.38) for myocardial infarction events and Andersen 2016 reported an adjusted IRR of 0.99 (95% CI: 0.70-1.40) for major adverse cardiac events. | | | There is large uncertainty over the effects of treatment for subclinical hypothyroidism on the occurrence of fatal and non-fatal cardiovascular events in females. |
| Fatal and non-fatal cardiovascular events (not including atrial fibrillation) - by SEX (Males) – Cohort  follow-up: median of 5.0-5.6 years | Range: 432^ii^ to 2,469^i^ per study  (2 OBS studies) ^k^ | ⨁◯◯◯ VERY LOW ^g,i,m,t^ | Both studies found no benefit of treatment for subclinical hypothyroidism in males on the occurrence of fatal and non-fatal cardiovascular events. Andersen 2015 reported an adjusted IRR of 1.41 (95% CI: 0.83-2.4) for myocardial infarction events and Andersen 2016 reported an adjusted IRR of 1.36 (95% CI: 0.79-2.35) for major adverse cardiac events. | | | There is large uncertainty over the effects of treatment for subclinical hypothyroidism on the occurrence of fatal and non-fatal cardiovascular events in males. |
| Atrial fibrillation - RCT follow up: range over 12 months to 3 years | 737 (1 RCT) ^a^ | ⨁⨁◯◯ LOW ^b,c,d,u^ | **HR 0.80** (0.35 to 1.80) | 35 per 1,000 | **7 fewer per 1,000** (23 fewer to 27 more) | There may be no statistically significant difference between those treated and not treated for subclinical hypothyroidism on the occurrence of new-onset atrial fibrillation events. |
| Atrial fibrillation - by AGE (Adults (40-70 years)) - Cohort  follow-up: median of 7.6 years | 3093 (1 OBS study) ^v^ | ⨁◯◯◯ VERY LOW ^b,c,i,u^ | **HR 0.76** (0.26 to 1.73) | 25 per 1,000 | **6 fewer per 1,000** (18 fewer to 18 more) | There is large uncertainty over the effects of treatment for subclinical hypothyroidism on the occurrence of atrial fibrillation events in younger adults. |
| Atrial fibrillation - by AGE (Adults (>65 years)) - RCT  follow up: range over 12 months to 3 years | 737 (1 RCT) ^a^ | ⨁⨁⨁◯ MODERATE ^b,c,i,u^ | **HR 0.80** (0.35 to 1.80) | 35 per 1,000 | **7 fewer per 1,000** (23 fewer to 27 more) | There is moderate certainty that no statistically significant difference exists between older adults treated and not treated for subclinical hypothyroidism on the occurrence of new-onset atrial fibrillation events. |
| Fractures follow up: range over 12 months to 3 years | 737 (1 RCT) ^a^ | ⨁⨁◯◯ LOW ^b,c,d,w^ | **HR 1.06** (0.41 to 2.76) | 22 per 1,000 | **1 more per 1,000** (13 fewer to 37 more) | There may be no statistically significant difference between those treated and not treated for subclinical hypothyroidism on the occurrence of fractures. |
| Thyroid-related Quality of Life - at 12 months | 638 (1 RCT) ^a^ | ⨁⨁⨁◯ MODERATE ^ab,b,c,d^ | MD of **0** (2 lower to 2.1 higher) on the ThyPRO Hypothyroid Symptoms scores | | | There is moderate certainty that no statistically significant difference exists between those treated and not treated for subclinical hypothyroidism on thyroid-related quality of life measures at 12 months follow-up. |
| Thyroid-related Quality of Life - at extended follow-up (over 12 months up to 3 years) | Range: 381 to 648 depending on outcome measure  (1 RCT) ^a^ | ⨁⨁⨁◯ MODERATE ^ab,b,c,d^ | There were no differences found between those treated and not treated for subclinical hypothyroidism on thyroid-specific measures of quality of life at extended follow-up. Stott 2017 reported a mean difference of 1.0 (95%CI: -1.9-3.9) on the ThyPRO Hypothyroid Symptoms scores and a mean difference of -0.5 (95%CI: -2.2-1.3) on the Comprehensive ThyPRO-39 scores between the treatment and control groups. | | | There is moderate certainty that no statistically significant difference exists between those treated and not treated for subclinical hypothyroidism on thyroid-related quality of life measures at extended follow-up. |
| Fatigue/Tiredness - at 12 months | 638 (1 RCT) ^a^ | ⨁⨁⨁◯ MODERATE ^ab,b,c,d^ | MD of **0.4 higher** (2.1 lower to 2.9 higher) on the ThyPRO Hypothyroid Tiredness scores at 12 months | | | There is moderate certainty that no statistically significant difference exists between those treated and not treated for subclinical hypothyroidism on fatigue/tiredness scores at 12 months follow-up. |
| Fatigue/Tiredness - at extended follow-up (over 12 months up to 3 years) | 381 (1 RCT) ^a^ | ⨁⨁⨁◯ MODERATE ^ab,b,c,d^ | MD of **3.5 lower** (7 lower to 0 ) on the ThyPRO Hypothyroid Tiredness scores at extended follow-up | | | There is moderate certainty that no statistically significant difference exists between those treated and not treated for subclinical hypothyroidism on fatigue/tiredness scores at extended follow-up. |
| Mental well-being follow up: range 12 weeks to 12 months | Range: 57 to 85 per study  (4 RCTs) ^ac^ | ⨁⨁⨁◯ MODERATE ^ad,ae,af,i^ | There were no differences found between those treated and not treated for subclinical hypothyroidism on measures of mental well-being.   \| Author \| Difference \| 95% CI \| \| --- \| --- \| --- \| \| Beck Depression Inventory \| \| \| \| Jorde 2006 \| 1.00* \| -0.80-2.80 \| \| Najafi 2015 \| 0.51* \| -4.74-5.76 \| \| Reuters 2012 \| -0.30** \| -3.12-2.52 \| \| Hamilton Scale for Anxiety \| \| \| \| Reuters 2012 \| 0.50** \| -2.81-3.81 \| \| Hamilton Scale for Depression \| \| \| \| Reuters 2012 \| -1.00** \| -2.49-0.49 \| \| Hospital Anxiety and Depression Scale (HADS) \| \| \| \| Parle 2010 \| 0.30* \| -0.86-1.46 \|   * Value is the difference in mean scores at final follow up between treatment and control groups except where noted.  ** Value is the difference in mean variation scores from baseline to follow-up between treatment and control group | | | There is moderate certainty that no statistically significant difference exists between those treated and not treated for subclinical hypothyroidism on measures of mental well-being. |
| Physical well-being follow up: range 12 months to 3 years | Range: 646 to 647 depending on outcome measure  (1 RCT) ^a^ | ⨁⨁⨁◯ MODERATE ^ag,ah,b,d^ | There were no differences found between those treated and not treated for subclinical hypothyroidism on measures of physical well-being. Stott 2017 reported a -0.1 difference (95%CI: -0.3-1.0) in both the Barthel Index basic activities of daily living and the OARS instrumental activities of daily living scores. | | | There is moderate certainty that no statistically significant difference exists between those treated and not treated for subclinical hypothyroidism on measures of physical well-being. |
| General well-being follow up: range 6 months to 12 months | Range: 57 to 371 per study  (3 RCTs) ^ai^ | ⨁⨁⨁◯ MODERATE ^aj,ak,al,i^ | There were no differences found between those treated and not treated for subclinical hypothyroidism on measures of general well-being.   \| Author \| Sample size (treatment group vs. control group) \| Difference* \| 95% CI \| \| --- \| --- \| --- \| --- \| \| EUROQUOL Group 5-Dimension Report Questionnaire Descriptive Score \| \| \| \| \| Stott 2017 \| 638 participants (318 vs. 320) \| -0.03* \| -0.05-0.00 (p=0.05) \| \| EUROQUOL Group 5-Dimension Report Questionnaire Visual Analogue Scale Score \| \| \| \| \| Stott 2017 \| 638 participants (318 vs. 320) \| -1.3* \| -3.2-0.6 \| \| General Health Questionnaire \| \| \| \| \| Jorde 2006 \| 69 participants (36 vs. 33) \| 0.70* \| -0.58-1.98 \| \| Medical Outcomes Study 36-item Short Form Health Survey \| \| \| \| \| Reuters 2012 \| 57 participants (25 vs. 32) \| 0.30** \| -0.43-1.03 \|   * Value is the difference in mean scores at final follow up between treatment and control groups except where noted.  ** Value is the difference in mean variation scores from baseline to follow-up between treatment and control group | | | There is moderate certainty that no statistically significant difference exists between those treated and not treated for subclinical hypothyroidism on measures of general well-being. |
| Overall Cognitive Function  follow-up: 12 months to 3 years | Range: 65 to 600 per study  (3 RCTs) ^x,y^ | ⨁⨁◯◯ LOW ^aa,i,u,z^ | One study showed a significant difference between those treated and not treated for subclinical hypothyroidism on the composite cognitive score, with those in the treatment group scoring slightly better than those in the control group. One study showed a statistically significant improvement among those treated for the SCOLP test. There were no differences found between those treated and not treated for subclinical hypothyroidism on the other measures of cognitive function.   \| Test \| Author \| Difference \| 95% CI \| \| --- \| --- \| --- \| --- \| \| California Computerized Assessment Package (CalCAP) \| Jorde 2006 \| -79.00 \| -229.88-71.88 \| \| Composite cognitive score \| Jorde 2006 \| 2.40 \| 0.29-4.51 \| \| Controlled Word Association test \| Jorde 2006 \| 0.10 \| -6.77-6.97 \| \| Letter Digit Coding test \| Stott 2017 \| -0.1 \| -0.9-0.7 \| \| Middlesex Elderly Assessment of Mental State (MEAMS) \| Parle 2010 \| 0.34 \| -0.08-0.76 \| \| Mini-Mental State Examination (MMSE) \| Parle 2010 \| 0.03 \| -0.89-0.95 \| \| Seashore Rhythm test \| Jorde 2006 \| -37.00 \| -89.85-15.85 \| \| Speed and Capacity of Language Processing test (SCOLP) \| Parle 2010 \| 1.47 \| 0.05-2.89 \| \| Trail Making Test A \| Jorde 2006 \| -5.10 \| -12.84-2.64 \| \| Parle 2010 \| -2.45 \| -11.10-6.20 \| \| Trail Making Test B \| Jorde 2006 \| -9.00 \| -35.79-17.79 \| \| Parle 2010 \| -11.71 \| -45.20-21.78 \| \| Trail Making Test B-A \| Parle 2010 \| -11.61 \| -37.91-14.69 \| \| Vocabulary – Wechsler Intelligence Scale \| Jorde 2006 \| 0.10 \| -1.73-1.93 \| \| Word List test \| Jorde 2006 \| 1.10 \| -2.63-4.83 \| | | | There may be no statistically significant differences between those treated and not treated for subclinical hypothyroidism on measures of cognitive function. |
| ***The risk in the intervention group** (and its 95% confidence interval) is based on the assumed risk in the comparison group and the **relative effect** of the intervention (and its 95% CI).   **CI:** Confidence interval; **HR:** Hazard Ratio; **OR:** Odds ratio; **MD:** Mean difference | | | | | |  |
| **GRADE Working Group grades of evidence** **High certainty:** We are very confident that the true effect lies close to that of the estimate of the effect **Moderate certainty:** We are moderately confident in the effect estimate: The true effect is likely to be close to the estimate of the effect, but there is a possibility that it is substantially different **Low certainty:** Our confidence in the effect estimate is limited: The true effect may be substantially different from the estimate of the effect **Very low certainty:** We have very little confidence in the effect estimate: The true effect is likely to be substantially different from the estimate of effect | | | | | |  |

#### Explanations

a. Stott 2017

b. The information is from a single study with a low risk of bias. Therefore we will not downgrade for risk of bias.

c. In this situation, the assessment of inconsistency is based on one study. Therefore, we will not downgrade for inconsistency.

d. These findings are from a study where all of the participants were >65 years of age. Since adults <65 years of age were not included in the study and because this information is meant to provide linked evidence to inform the CTFPHC's recommendation on screening for thyroid dysfunction in adults <65 years of age as well, then we will rate down for indirectness.

e. We will downgrade for imprecision because the upper confidence limit of the estimate crosses a clinically important threshold (an increase in mortality of >=1 death). In addition, the study was underpowered to detect an effect of treatment on this outcome.

f. Andersen 2015, Razvi 2012

g. The information is from cohort studies with a low risk of bias. Therefore we will not downgrade for risk of bias.

h. Assuming a constant hazard over time, the IRR would be similar to the HR and can be assessed for inconsistency. Although the confidence intervals of the 2 studies overlap; the point estimates are not very close to each other. Therefore we will downgrade for inconsistency.

i. These findings are from studies addressing the key question on treatment. Although this is meant to provide linked evidence to inform the CTFPHC's recommendation on screening for thyroid dysfunction, indirectness of the evidence to the question on screening will be addressed in the Evidence-to-Decision framework. Therefore we will not rate down for indirectness in the GRADE tables.

j. The upper confidence limit of the estimates do not cross a clinically important threshold (an increase in mortality of >=1 death) and the effect sizes are not implausibly large. Therefore we will not downgrade for imprecision.

k. Andersen 2015, Andersen 2016

l. The point estimates are the same and the confidence intervals overlap; therefore we will not downgrade for inconsistency.

m. We will downgrade for imprecision because the upper confidence limit of the estimates cross a clinically important threshold (an increase in mortality of >=1 death).

n. Although the confidence intervals of the 2 studies overlap; the point estimates are not very close to each other. Therefore we will downgrade for inconsistency.

o. We will downgrade for imprecision due to very few events for which comparisons between the treatment and control groups could not be made and because the study was underpowered to detect an effect of treatment on this outcome.

p. Assuming a constant hazard over time, the IRR would be similar to the HR and can be assessed for inconsistency. The point estimates are similar and the confidence intervals overlap; therefore we will not downgrade for inconsistency.

q. Andersen 2015

r. Assuming a constant hazard over time, the IRR would be similar to the HR and can be assessed for inconsistency. Although the confidence intervals overlap; the point estimates are not close together. However, the estimates are for different types of fatal and non-fatal cardiovascular events which may explain the variability. Therefore we will not downgrade for inconsistency.

s. We will downgrade for imprecision because upper confidence limit of 2/3 estimates cross a clinically important threshold (an increase in mortality of >=1 death).

t. The point estimates are similar and the confidence intervals overlap; therefore we will not downgrade for inconsistency.

u. We will downgrade for imprecision because the optimal information size was not met for this outcome.

v. Razvi 2012

w. We will downgrade for imprecision because the upper confidence limit of the estimate likely crosses a clinically important threshold and because the optimal information size was not met for this outcome.

x. Jorde 2006, Parle 2010, Stott 2017

y. Includes the following measures: Composite Cognitive Score (Used in one study and was made by adding together the Z-scores for the following tests: Digit span forward and backward, Stroop tests parts 1, 2 and 3, Verbal and visual recall), Letter Digit Coding Test (LDCT), Trail Making Tests A, B and B-A, Seashore Rhythm test, Word List test, Controlled Word Association test, California Computerized Assessment Package (CalCAP), WAIS Vocabulary, Middlessex Elderly Assessment of Mental State (MEAMS), Speed and Language Processing test (SCOLP), and the Mini-Mental State Examination (MMSE).

z. The information is from studies with a low risk of bias for blinding of participants, personnel and outcome assessment and we do not believe that any uncertain biases (i.e. for sequence generation or allocation concealment) impacted the results. Therefore we will not downgrade for risk of bias.

aa. Inconsistency may be explained due to the many tools/tests used to assess various aspects of cognitive function. However, even though all of the confidence intervals overlap, the point estimates were often far apart from each other. Therefore we will downgrade for inconsistency.

ab. The upper and lower confidence limits of the estimate did not cross a clinically important threshold (i.e. used a minimum clinically meaningful difference threshold of 9 points, as suggested by the ThyPRO author). Therefore we will not downgrade for imprecision.

ac. Jorde 2006, Najafi 2015, Parle 2010, Reuters 2012

ad. The information is mostly from studies with a low risk of bias for blinding of participants, personnel and outcome assessment and we do not believe that any uncertain biases (i.e. for sequence generation or allocation concealment) impacted the results. Therefore we will not downgrade for risk of bias.

ae. Inconsistency may be explained due to the many tools/tests used to assess various aspects of mental well-being. Because the confidence intervals overlap and the point estimates were fairly close to each other, therefore we will not downgrade for inconsistency.

af. We will downgrade for imprecision due to small sample sizes.

ag. The point estimates and the confidence intervals are exactly the same, therefore we will not downgrade for inconsistency.

ah. The estimates likely do not cross a clinically important threshold and the sample size is fairly moderate therefore we will not downgrade for imprecision.

ai. Jorde 2006, Reuters 2012, Stott 2017

aj. The information is from studies with a low risk of bias for blinding of participants, personnel and outcome assessment and we do not believe that any uncertain biases (i.e. for sequence generation or allocation concealment) impacted the results. Therefore we will not downgrade for risk of bias.

ak. Inconsistency may be explained due to the many tools/tests used to assess various aspects of general well-being. Because the confidence intervals overlap and the point estimates were fairly close to each other, therefore we will not downgrade for inconsistency.

al. Although the estimates likely do not cross a clinically important threshold, the sample sizes are fairly small across studies, therefore we will downgrade for imprecision.

**Table 1.2 GRADE Evidence Profile Table – morbidity and mortality outcomes**

**Author(s)**: GHGD Science Team

**Question**: 3a. Does treatment of screen-detected overt or subclinical thyroid dysfunction improve morbidity or mortality?

**Setting**: Primary Care in Canada

| **Certainty assessment** | | | | | | | **№ of patients** | | **Effect** | | **Certainty** | **Importance** |
| --- | --- | --- | --- | --- | --- | --- | --- | --- | --- | --- | --- | --- |
| **№ of studies** | **Study design** | **Risk of bias** | **Inconsistency** | **Indirectness** | **Imprecision** | **Other considerations** | **treatment** | **no treatment** | **Relative (95% CI)** | **Absolute (95% CI)** |  |  |
| All-cause mortality - RCT (follow up: range 12 months to 3 years) | | | | | | | | | | | | |
| 1 ^a^ | randomised trials | not serious ^b^ | not serious ^c^ | serious ^d^ | serious ^e^ | none | 10/368 (2.7%) | 5/369 (1.4%) | **HR 1.91** (0.65 to 5.60) | **12 more per 1,000** (from 5 fewer to 60 more) | ⨁⨁◯◯ LOW | CRITICAL |
| All-cause mortality by AGE ((Adults (<65 years or 40-70 years)) - Cohort | | | | | | | | | | | | |
| 2 ^f^ | observational studies | not serious ^g^ | serious ^h^ | not serious ^i^ | not serious ^j^ | none | Both studies showed a beneficial effect of treatment for subclinical hypothyroidism in adults <65 years or between 40-70 years of age for all-cause mortality. Andersen 2015 reported an adjusted IRR of 0.63 (95% CI: 0.40-0.99) and Razvi 2012 reported a multivariate adjusted HR of 0.36 (95% CI: 0.19-0.66) [absolute value of 41 fewer deaths per 1,000, ranging from 52 fewer to 21 fewer). | | | | ⨁◯◯◯ VERY LOW | CRITICAL |
| All-cause mortality by AGE (Adults(>65 years)) - RCT (follow up: range 12 months to 3 years) | | | | | | | | | | | | |
| 1 ^a^ | randomised trials | not serious ^b^ | not serious ^c^ | not serious ^i^ | serious ^e^ | none | 10/368 (2.7%) | 5/369 (1.4%) | **HR 1.91** (0.65 to 5.60) | **12 more per 1,000** (from 5 fewer to 60 more) | ⨁⨁⨁◯ MODERATE | CRITICAL |
| All-cause mortality by SEX (Females) - Cohort | | | | | | | | | | | | |
| 2 ^k^ | observational studies | not serious ^g^ | not serious ^l^ | not serious ^i^ | serious ^m^ | none | Both studies found no benefit of treatment in females for subclinical hypothyroidism on all-cause mortality. Andersen 2015 reported an adjusted IRR of 0.99 (95% CI: 0.85-1.16) and Andersen 2016 reported an adjusted IRR of 1.08 (95% CI: 0.80-1.48). | | | | ⨁◯◯◯ VERY LOW | CRITICAL |
| All-cause mortality by SEX (Males) - Cohort | | | | | | | | | | | | |
| 2 ^k^ | observational studies | not serious ^g^ | serious ^n^ | not serious ^i^ | serious ^m^ | none | Both studies found no benefit of treatment for subclinical hypothyroidism in males on all-cause mortality. Andersen 2015 reported an adjusted IRR of 1.24 (95% CI: 0.89-1.16) and Andersen 2016 reported an adjusted IRR of 1.43 (95% CI: 0.87-2.34). | | | | ⨁◯◯◯ VERY LOW | CRITICAL |
| Deaths due to cardiovascular diseases - RCT (follow up: range 12 months to 3 years) | | | | | | | | | | | | |
| 1 ^a^ | randomised trials | not serious ^b^ | not serious ^c^ | serious ^d^ | very serious ^o^ | none | 2/368 (0.5%) | 1/369 (0.3%) | **OR 2.01** (0.18 to 22.30) | **3 more per 1,000** (from 2 fewer to 54 more) | ⨁◯◯◯ VERY LOW | CRITICAL |
| Deaths due to cardiovascular diseases by AGE (Adults (<65 years or 40-70 years)) - Cohort | | | | | | | | | | | | |
| 2 ^f^ | observational studies | not serious ^g^ | not serious ^p^ | not serious ^i^ | serious ^m^ | none | Razvi 2012 showed a beneficial effect of treatment for subclinical hypothyroidism in patients between 40-70 years on deaths due to circulatory diseases only (multivariate adjusted HR of 0.54 (95% CI: 0.37-0.92); absolute value of 12 fewer deaths per 1,000 (ranged from 16 fewer to 2 fewer)). However, Andersen 2015 found no beneficial effect of treatment on cardiovascular deaths (adjusted IRR of 0.55 (95% CI: 0.25-1.20)). | | | | ⨁◯◯◯ VERY LOW | CRITICAL |
| Deaths due to cardiovascular diseases by AGE (Adults (>65 years)) - RCT (follow up: range 12 months to 3 years) | | | | | | | | | | | | |
| 1 ^a^ | randomised trials | not serious ^g^ | not serious ^c^ | not serious ^i^ | very serious ^o^ | none | 2/368 (0.5%) | 1/369 (0.3%) | **OR 2.01** (0.18 to 22.27) | **3 more per 1,000** (from 2 fewer to 54 more) | ⨁⨁◯◯ LOW | CRITICAL |
| Deaths due to cardiovascular diseases by SEX (Females) - Cohort | | | | | | | | | | | | |
| 1 ^q^ | observational studies | not serious ^b^ | not serious ^c^ | not serious ^i^ | serious ^m^ | none | There were no differences found in females treated and not treated for subclinical hypothyroidism on cardiovascular-related deaths (adjusted IRR of 0.96, 95% CI: 0.77-1.12). | | | | ⨁◯◯◯ VERY LOW | CRITICAL |
| Deaths due to cardiovascular diseases by SEX (Males) - Cohort | | | | | | | | | | | | |
| 1 ^q^ | observational studies | not serious ^b^ | not serious ^c^ | not serious ^i^ | serious ^m^ | none | There were no differences found in males treated and not treated for subclinical hypothyroidism on cardiovascular-related deaths (adjusted IRR of 1.32, 95% CI: 0.83-2.08). | | | | ⨁◯◯◯ VERY LOW | CRITICAL |
| Fatal and non-fatal cardiovascular events (not including atrial fibrillation) - RCT (follow up: range 12 months to 3 years) | | | | | | | | | | | | |
| 1 ^a^ | randomised trials | not serious ^b^ | not serious ^c^ | serious ^d^ | serious ^e^ | none | 18/368 (4.9%) | 20/369 (5.4%) | **HR 0.89** (0.47 to 1.69) | **6 fewer per 1,000** (from 28 fewer to 36 more) | ⨁⨁◯◯ LOW | CRITICAL |
| Fatal and non-fatal cardiovascular events - by AGE (Adults (<65 years or 40-70 years)) - Cohort | | | | | | | | | | | | |
| 2 ^f^ | observational studies | not serious ^g^ | not serious ^r^ | not serious ^i^ | serious ^s^ | none | Razvi 2012 showed a beneficial effect of treatment for subclinical hypothyroidism in patients between 40-70 years on the occurrence of fatal and non-fatal ischemic heart disease events (multivariate adjusted HR of 0.61, 95% CI: 0.39-0.95; absolute value of 25 fewer per 1,000 (ranged from 3 fewer to 40 fewer)) but not on the occurrence of fatal and non-fatal cardiovascular disease (multivariate adjusted HR of 1.03, 95% CI: 0.51-2.13; absolute value of 1 more per 1,000 (ranged from 15 fewer to 27 more)). Andersen 2015 found no benefit of treatment on the occurrence of myocardial infarction (adjusted IRR of 1.11, 95% CI: 0.61-2.02). | | | | ⨁◯◯◯ VERY LOW | CRITICAL |
| Fatal and non-fatal cardiovascular events (not including atrial fibrillation) - by AGE (Adults (>65 years)) - RCT (follow up: range 12 months to 3 years) | | | | | | | | | | | | |
| 1 ^a^ | randomised trials | not serious ^b^ | not serious ^c^ | not serious ^i^ | serious ^e^ | none | 18/368 (4.9%) | 20/369 (5.4%) | **HR 0.89** (0.47 to 1.69) | **6 fewer per 1,000** (from 28 fewer to 36 more) | ⨁⨁⨁◯ MODERATE | CRITICAL |
| Fatal and non-fatal cardiovascular events - by SEX (Females) - Cohort | | | | | | | | | | | | |
| 2 ^k^ | observational studies | not serious ^g^ | not serious ^t^ | not serious ^i^ | serious ^m^ | none | Both studies found no benefit of treatment for subclinical hypothyroidism in females on the occurrence of fatal and non-fatal cardiovascular events. Andersen 2015 reported an adjusted IRR of 0.98 (95% CI: 0.70-1.38) for myocardial infarction events and Andersen 2016 reported an adjusted IRR of 0.99 (95% CI: 0.70-1.40) for major adverse cardiac events. | | | | ⨁◯◯◯ VERY LOW | CRITICAL |
| Fatal and non-fatal cardiovascular events - by SEX (Males) - Cohort | | | | | | | | | | | | |
| 2 ^k^ | observational studies | not serious ^g^ | not serious ^t^ | not serious ^i^ | serious ^m^ | none | Both studies found no benefit of treatment for subclinical hypothyroidism in males on the occurrence of fatal and non-fatal cardiovascular events. Andersen 2015 reported an adjusted IRR of 1.41 (95% CI: 0.83-2.4) for myocardial infarction events and Andersen 2016 reported an adjusted IRR of 1.36 (95% CI: 0.79-2.35) for major adverse cardiac events. | | | | ⨁◯◯◯ VERY LOW | CRITICAL |
| Atrial fibrillation - RCT (follow up: range 12 months to 3 years) | | | | | | | | | | | | |
| 1 ^a^ | randomised trials | not serious ^b^ | not serious ^c^ | serious ^d^ | serious ^u^ | none | 11/368 (3.0%) | 13/369 (3.5%) | **HR 0.80** (0.35 to 1.80) | **7 fewer per 1,000** (from 23 fewer to 27 more) | ⨁⨁◯◯ LOW | CRITICAL |
| Atrial fibrillation - by AGE (Adults (40-70 years)) - Cohort | | | | | | | | | | | | |
| 1 ^v^ | observational studies | not serious ^b^ | not serious ^c^ | not serious ^i^ | serious ^u^ | none | 35/1634 (2.1%) | 36/1459 (2.5%) | **HR 0.76** (0.26 to 1.73) | **6 fewer per 1,000** (from 18 fewer to 18 more) | ⨁◯◯◯ VERY LOW | CRITICAL |
| Atrial fibrillation - by AGE (Older adults (>65 years)) - RCT | | | | | | | | | | | | |
| 1 ^a^ | randomised trials | not serious ^b^ | not serious ^c^ | not serious ^i^ | serious ^u^ | none | 11/368 (3.0%) | 13/369 (3.5%) | **HR 0.80** (0.35 to 1.80) | **7 fewer per 1,000** (from 23 fewer to 27 more) | ⨁⨁⨁◯ MODERATE | CRITICAL |
| Fractures (follow up: range 12 months to 3 years) | | | | | | | | | | | | |
| 1 ^a^ | randomised trials | not serious ^b^ | not serious ^c^ | serious ^d^ | serious ^w^ | none | 9/368 (2.4%) | 8/369 (2.2%) | **HR 1.06** (0.41 to 2.76) | **1 more per 1,000** (from 13 fewer to 37 more) | ⨁⨁◯◯ LOW | CRITICAL |
| Thyroid-related Quality of Life - at 12 months | | | | | | | | | | | | |
| 1 ^a^ | randomised trials | not serious ^b^ | not serious ^c^ | serious ^d^ | not serious ^ab^ | none | 318 | 320 | - | MD **0**  (2 lower to 2.1 higher) | ⨁⨁⨁◯ MODERATE | CRITICAL |
| Thyroid-related Quality of Life - at extended follow-up (up to 3 years) | | | | | | | | | | | | |
| 1 ^a^ | randomised trials | not serious ^b^ | not serious ^c^ | serious ^d^ | not serious ^ab^ | none | There were no differences found between those treated and not treated for subclinical hypothyroidism on thyroid-specific measures of quality of life at extended follow-up. Stott 2017 reported a difference of 1.0 (95%CI: -1.9-3.9) on the ThyPRO Hypothyroid Symptoms scores and a difference of -0.5 (95%CI: -2.2-1.3) on the Comprehensive ThyPRO-39 scores between the treatment and control groups. | | | | ⨁⨁⨁◯ MODERATE | CRITICAL |
| Fatigue/Tiredness - at 12 months | | | | | | | | | | | | |
| 1 ^a^ | randomised trials | not serious ^b^ | not serious ^c^ | serious ^d^ | not serious ^ab^ | none | 318 | 320 | - | MD **0.4 higher** (2.1 lower to 2.9 higher) | ⨁⨁⨁◯ MODERATE | IMPORTANT |
| Fatigue/Tiredness - at extended follow-up | | | | | | | | | | | | |
| 1 ^a^ | randomised trials | not serious ^b^ | not serious ^c^ | serious ^d^ | not serious ^ab^ | none | 194 | 187 | - | MD **3.5 lower** (7 lower to 0 ) | ⨁⨁⨁◯ MODERATE | IMPORTANT |
| Mental well-being (follow up: range 12 weeks to 12 months) | | | | | | | | | | | | |
| 4 ^ac^ | randomised trials | not serious ^ad^ | not serious ^ae^ | not serious ^i^ | serious ^af^ | none | There were no differences found between those treated and not treated for subclinical hypothyroidism on measures of mental well-being. | | | | ⨁⨁⨁◯ MODERATE | CRITICAL |
| Physical well-being (follow up: range 12 months to 3 years) | | | | | | | | | | | | |
| 1 ^a^ | randomised trials | not serious ^b^ | not serious ^ag^ | serious ^d^ | not serious ^ah^ | none | There were no differences found between those treated and not treated for subclinical hypothyroidism on measures of physical well-being. Stott 2017 reported a -0.1 difference (95%CI: -0.3-1.0) in both the Barthel Index basic activities of daily living and the OARS instrumental activities of daily living scores. | | | | ⨁⨁⨁◯ MODERATE |  |
| General well-being (follow up: range 6 months to 12 months) | | | | | | | | | | | | |
| 3 ^ai^ | randomised trials | not serious ^aj^ | not serious ^ak^ | not serious ^i^ | serious ^al^ | none | Stott 2017 showed a minor improvement in EQ-5D descriptive scores in those ≥65 years of age treated for subclinical hypothyroidism (MD of 0.03, 95%CI: 0.05-0.00) but showed no statistically significant difference in the EQ-5D visual analogue scale scores. The other studies (Jorde 2006 and Reuters 2012) found no statistically significant difference in the the scores for GHQ-30 or SF-36 between those treated and not treated for subclinical hypothyroidism. | | | | ⨁⨁⨁◯ MODERATE |  |
| Overall Cognitive Function | | | | | | | | | | | | |
| 3 ^x,y^ | randomised trials | not serious ^z^ | serious ^aa^ | not serious ^i^ | serious ^u^ | none | One study showed a significant difference between those treated and not treated for subclinical hypothyroidism on the composite cognitive score, with those in the treatment group scoring slightly better than those in the control group. One study showed a significant improvement among those treated for the Speed and Capacity of Language Processing test. There were no differences found between those treated and not treated for subclinical hypothyroidism on the other measures of cognitive function. | | | | ⨁⨁◯◯ LOW | CRITICAL |

**CI:** Confidence interval; **HR:** Hazard Ratio; **OR:** Odds ratio; **MD:** Mean difference

#### Explanations

a. Stott 2017

b. The information is from a single study with a low risk of bias. Therefore we will not downgrade for risk of bias.

c. In this situation, the assessment of inconsistency is based on one study. Therefore, we will not downgrade for inconsistency.

d. These findings are from a study where all of the participants were >=65 years of age. Since adults <65 years of age were not included in the study and because this information is meant to provide linked evidence to inform the CTFPHC's recommendation on screening for thyroid dysfunction in adults <65 years of age as well, then we will rate down for indirectness.

e. We downgraded for imprecision because the upper confidence limit of the estimate crosses a clinically important threshold (an increase in mortality of >=1 death). In addition, the study was underpowered to detect an effect of treatment on this outcome.

f. Andersen 2015, Razvi 2012

g. The information are from cohort studies with a low risk of bias. Therefore we will not downgrade for risk of bias.

h. Assuming a constant hazard over time, the IRR would be similar to the HR and can be assessed for inconsistency. Although the confidence intervals of the 2 studies overlap; the point estimates are not very close to each other. Therefore we will downgrade for inconsistency.

i. These findings are from studies addressing the key question on treatment. Although this is meant to provide indirect evidence to inform the CTFPHC's recommendation on screening for thyroid dysfunction, indirectness of the evidence to the question on screening will be addressed in the Evidence-to-Decision framework. Therefore we will not rate down for indirectness in the GRADE tables.

j. The upper confidence limit of the estimates do not cross a clinically important threshold (an increase in mortality of >=1 death) and the effect sizes are not implausibly large. Therefore we will not downgrade for imprecision.

k. Andersen 2015, Andersen 2016

l. The point estimates are the same and the confidence intervals overlap; therefore we will not downgrade for inconsistency.

m. We downgraded for imprecision because the upper confidence limit of the estimates cross a clinically important threshold (an increase in mortality of >=1 death).

n. Although the confidence intervals of the 2 studies overlap; the point estimates are not very close to each other. Therefore we will downgrade for inconsistency.

o. We will downgrade for imprecision due to very few events for which comparisons between the treatment and control groups could not be made and because the study was underpowered to detect an effect of treatment on this outcome.

p. Assuming a constant hazard over time, the IRR would be similar to the HR and can be assessed for inconsistency. The point estimates are similar and the confidence intervals overlap; therefore we will not downgrade for inconsistency.

q. Andersen 2015

r. Assuming a constant hazard over time, the IRR would be similar to the HR and can be assessed for inconsistency. Although the confidence intervals overlap; the point estimates are not close together. However, the estimates are for different types of fatal and non-fatal cardiovascular events which may explain the variability. Therefore we will not downgrade for inconsistency.

s. We downgraded for imprecision because upper confidence limit of 2/3 estimates cross a clinically important threshold (an increase in mortality of >=1 death).

t. The point estimates are similar and the confidence intervals overlap; therefore we will not downgrade for inconsistency.

u. We downgraded for imprecision because the optimal information size was not met for this outcome.

v. Razvi 2012

w. We will downgrade for imprecision because the upper confidence limit of the estimate likely crosses a clinically important threshold and because the optimal information size was not met for this outcome.

x. Jorde 2006, Parle 2010, Stott 2017

y. Includes the following measures: Composite Cognitive Score (Used in one study and was made by adding together the Z-scores for the following tests: Digit span forward and backward, Stroop tests parts 1, 2 and 3, Verbal and visual recall), Letter Digit Coding Test (LDCT), Trail Making Tests A, B and B-A, Seashore Rhythm test, Word List test, Controlled Word Association test, California Computerized Assessment Package (CalCAP), WAIS Vocabulary, Middlessex Elderly Assessment of Mental State (MEAMS), Speed and Language Processing test (SCOLP), and the Mini-Mental State Examination (MMSE)

z. The information is from studies with a low risk of bias for blinding of participants, personnel and outcome assessment and we do not believe that any uncertain biases (i.e. for sequence generation or allocation concealment) impacted the results. Therefore we will not downgrade for risk of bias.

aa. Inconsistency may be explained due to the many tools/tests used to assess various aspects of cognitive function. However, even though all of the confidence intervals overlap, the point estimates were often far apart from each other. Therefore we will downgrade for inconsistency.

ab. The upper and lower confidence limits of the estimate did not cross a clinically important threshold (i.e. used a minimum clinically meaningful difference threshold of 9 points, as suggested by the ThyPRO author). Therefore we will not downgrade for imprecision.

ac. Jorde 2006, Najafi 2015, Parle 2010, Reuters 2012

ad. The information is from mostly studies with a low risk of bias for blinding of participants, personnel and outcome assessment and we do not believe that any uncertain biases (i.e. for sequence generation or allocation concealment) impacted the results. Therefore we will not downgrade for risk of bias.

ae. Inconsistency may be explained due to the many tools/tests used to assess various aspects of mental well-being. Because the confidence intervals overlap and the point estimates were fairly close to each other, therefore we will not downgrade for inconsistency.

af. We will downgrade for imprecision due to small sample sizes.

ag. The point estimates and the confidence intervals are exactly the same, therefore we will not downgrade for inconsistency.

ah. The estimates likely do not cross a clinically important threshold and the sample size is fairly moderate therefore we will not downgrade for imprecision.

ai. Jorde 2006, Reuters 2012, Stott 2017

aj. The information is from studies with a low risk of bias for blinding of participants, personnel and outcome assessment and we do not believe that any uncertain biases (i.e. for sequence generation or allocation concealment) impacted the results. Therefore we will not downgrade for risk of bias.

ak. Inconsistency may be explained due to the many tools/tests used to assess various aspects of general well-being. Because the confidence intervals overlap and the point estimates were fairly close to each other, therefore we will not downgrade for inconsistency.

al. Although the estimates likely do not cross a clinically important threshold, the sample sizes are fairly small across studies, therefore we will downgrade for imprecision.

**Evidence Set 2**

**KQ3b: Does treatment of screen-detected overt or subclinical thyroid dysfunction improve intermediate outcomes?**

**Table 2.1 GRADE Summary of Findings Table – intermediate outcomes**

| **3b. Does treatment of screen-detected overt or subclinical thyroid dysfunction improve intermediate outcomes?  Narrative** | | | |  |
| --- | --- | --- | --- | --- |
| **Outcomes, Follow-up** | **№ of participants (№ of studies)** | **Certainty of the evidence (GRADE)** | **Impact** | **Comments** |
| Bone Mineral Density | (0 studies) | - |  | No studies reported on the effects of treatment versus no treatment for thyroid dysfunction on bone mineral density. |
| Total Cholesterol follow up: range 5 months to 12 months | 958 (10 RCTs) ^a^ | ⨁⨁⨁◯ MODERATE ^b,c,d,e^ | Results were mixed. Six studies did not find a statistically significant difference in total cholesterol (TC) levels between those treated and not treated for subclinical hypothyroidism. Three studies found that mean values for TC levels at final follow-up were less in the treatment group compared to the control group and the findings were statistically significant. One study found the decline in mean TC levels from baseline to follow-up was statistically significantly larger in the treatment group than in the control group. Difference in means between treatment and control groups at final follow-up ranged from -1.07 to 0.00 mmol/L.  Difference in means between Treatment and Control groups at final follow-up:   \| Author \| Difference \| 95% CI \| \| --- \| --- \| --- \| \| Cabral 2011 \| -1.07 mmol/L* \| -2.49-0.36 \| \| Caraccio 2002 \| -0.30 mmol/L* \| -0.92-0.32 \| \| Duman 2007 \| 0.00 mmol/L* \| -0.98-0.98 \| \| Iqbal 2006 \| -0.10 mmol/L* \| -0.59-0.39 \| \| Liu 2015 \| -0.29 mmol/L** \| -0.54-(-0.04) \| \| Mikhail 2008 \| -0.30 mmol/L* \| -0.58-(-0.30) \| \| Monzani 2004 \| -1.56 mmol/L* \| -2.91-(-0.20) \| \| Nagasaki 2009 \| -0.14 mmol/L** \| -0.54-0.26 \| \| Teixeira 2008 \| -0.32 mmol/L* \| -1.79-1.16 \|   * Value is the difference in mean scores at final follow up between treatment and control groups.** Value is the difference in mean variation scores from baseline to follow-up between treatment and control group.***Values reported as mg/dL were converted to mmol/L.  Comparison of change from baseline values to final follow-up between Treatment and Control groups:   \| Author \| Results \| p-value \| \| --- \| --- \| --- \| \| Zhao 2016 \| The decline in the treatment group (-0.41 mmol/L) was statistically significantly greater than the decline in the control group (-0.17 mmol/L) \| p = 0.012 \| | There is moderate certainty that there may be little to no difference between those treated and not treated for subclinical hypothyroidism on total cholesterol levels. |
| Low-density Lipoprotein follow up: range 5 months to 12 months | 589 (9 RCTs) ^f^ | ⨁⨁⨁◯ MODERATE ^b,c,d,e^ | The majority of studies (8/9) did not find a difference in low-density lipoprotein (LDL) levels between those treated and not treated for subclinical hypothyroidism. Difference in means between treatment and control groups at final follow-up ranged from -1.23 to 0.11 mmol/L.  Difference in means between Treatment and Control groups at final follow-up:   \| Author \| Difference \| 95% CI \| \| --- \| --- \| --- \| \| Cabral 2011 \| -0.99 mmol/L* \| -2.40-0.42 \| \| Caraccio 2002 \| -0.30 mmol/L* \| -0.83-0.23 \| \| Duman 2007 \| 0.11 mmol/L* \| -0.89-1.11 \| \| Iqbal 2006 \| 0.00 mmol/L* \| -0.47-0.47 \| \| Liu 2015 \| -0.12 mmol/L** \| -0.32-0.08 \| \| Mikhail 2008 \| -0.21 mmol/L* \| -0.46-0.03 \| \| Monzani 2004 \| -1.23 mmol/L* \| -2.32-(-0.13) \| \| Nagasaki 2009 \| -0.22 mmol/L** \| -0.70-0.26 \| \| Teixeira 2008 \| -0.63 mmol/L* \| -1.90-0.64 \|   * Value is the difference in mean scores at final follow up between treatment and control groups.** Value is the difference in mean variation scores from baseline to follow-up between treatment and control group.***Values reported as mg/dL were converted to mmol/L. | There is moderate certainty that no statistically significant difference exists between those treated and not treated for subclinical hypothyroidism on low-density lipoprotein levels. |
| High-density Lipoprotein follow up: range 5 months to 12 months | 589 (9 RCTs) ^f^ | ⨁⨁⨁◯ MODERATE ^b,c,d,e^ | No studies found a difference in high-density lipoprotein (HDL) levels between those treated and not treated for subclinical hypothyroidism. Difference in means between treatment and control groups at final follow-up ranged from -0.17 to 0.26 mmol/L.  Difference in means between Treatment and Control groups at final follow-up:   \| Author \| Difference \| 95% CI \| \| --- \| --- \| --- \| \| Cabral 2011 \| 0.26 mmol/L* \| -0.17-0.70 \| \| Caraccio 2002 \| -0.10 mmol/L* \| -0.27-0.07 \| \| Duman 2007 \| 0.004 mmol/L* \| -0.45-0.44 \| \| Iqbal 2006 \| 0.00 mmol/L* \| -0.22-0.22 \| \| Liu 2015 \| 0.03 mmol/L** \| -0.03-0.09 \| \| Mikhail 2008 \| 0.09 mmol/L* \| -0.01-0.20 \| \| Monzani 2004 \| -0.17 mmol/L* \| -0.49-0.14 \| \| Nagasaki 2009 \| 0.02 mmol/L** \| -0.12-0.16 \| \| Teixeira 2008 \| 0.35 mmol/L* \| -0.29-0.99 \|   *Value is the difference in mean scores at final follow up between treatment and control groups.**Value is the difference in mean variation scores from baseline to follow-up between treatment and control group.***Values reported as mg/dL were converted to mmol/L. | There is moderate certainty that no statistically significant difference exists between those treated and not treated for subclinical hypothyroidism on high-density lipoprotein levels. |
| Triglycerides follow up: range 5 months to 12 months | 958 (10 RCTs) ^a^ | ⨁⨁⨁◯ MODERATE ^b,c,d,e^ | All but one study found no difference in triglyceride levels between those treated and not treated for subclinical hypothyroidism. Difference in means between treatment and control groups at final follow-up ranged from -1.72 to 0.12 mmol/L.  Difference in means between Treatment and Control groups at final follow-up:   \| Author \| Difference \| 95% CI \| \| --- \| --- \| --- \| \| Cabral 2011 \| -1.72 mmol/L* \| -3.51-0.07 \| \| Caraccio 2002 \| -0.10 mmol/L* \| -0.46-0.26 \| \| Duman 2007 \| -1.94 mmol/L* \| -3.65-(-0.24) \| \| Iqbal 2006 \| -0.10 mmol/L* \| -0.52-0.32 \| \| Liu 2015 \| -0.08 mmol/L** \| -0.26-0.10 \| \| Mikhail 2008 \| -0.11 mmol/L* \| -0.31-0.09 \| \| Monzani 2004 \| -0.81 mmol/L* \| -2.22-0.60 \| \| Nagasaki 2009 \| 0.12 mmol/L** \| -0.17-0.41 \| \| Teixeira 2008 \| -0.98 mmol/L* \| -3.52-1.55 \|   * Value is the difference in mean scores at final follow up between treatment and control groups.**Value is the difference in mean variation scores from baseline to follow-up between treatment and control group.***Values reported as mg/dL were converted to mmol/L.  Comparison of change from baseline values to final follow-up between Treatment and Control groups   \| Author \| Results \| p-value \| \| --- \| --- \| --- \| \| Zhao 2016 \| The decline in the control group (-0.11 mmol/L) was similar to the decline in the treatment group (-0.17 mmol/L) \| p≥0.05 \| | There is moderate certainty that no statistically significant difference exists between those treated and not treated for subclinical hypothyroidism on triglyceride levels. |
| Systolic Blood Pressure follow up: range 5 months to 15 months | 1354 (8 RCTs) ^g^ | ⨁⨁⨁◯ MODERATE ^d,h,i,j^ | No studies found a difference in systolic blood pressure measurements at final follow-up between those treated and not treated for subclinical hypothyroidism. Difference in means between treatment and control groups at final follow-up ranged from -12.25 to 0.5 mmHg.  Difference in means between Treatment and Control groups at final follow-up:   \| Author \| Difference \| 95% CI \| \| --- \| --- \| --- \| \| Liu 2015 \| -1.00 mmHg** \| -3.87-1.87 \| \| Mainenti 2009 \| -12.25 mmHg* \| -29.53-5.03 \| \| Monzani 2001 \| 0.50 mmHg* \| -6.79-7.79 \| \| Monzani 2004 \| -2.00 mmHg* \| -10.19-6.19 \| \| Nagasaki 2009 \| -3.40 mmHg** \| -10.56-3.76 \| \| Stott 2017 \| -0.10 mmHg* \| -2.1-2.4 \| \| Yazici 2004 \| 0.50 mmHg* \| -5.23-6.23 \| \| Zhao 2016 \| -2.54 mmHg* \| -6.65-1.57 \|   * Value is the difference in mean scores at final follow up between treatment and control groups.**Value is the difference in mean variation scores from baseline to follow-up between treatment and control group. | There is moderate certainty that no statistically significant difference exists between those treated and not treated for subclinical hypothyroidism on systolic blood pressure. |
| Diastolic Blood Pressure follow up: range 5 months to 15 months | 1354 (8 RCTs) ^g^ | ⨁⨁⨁◯ MODERATE ^d,h,i,j^ | No studies found a difference in diastolic blood pressure measurements at final follow-up between those treated and not treated for subclinical hypothyroidism. Difference in means between treatment and control groups at follow-up ranged from -5.4 to 3.8 mmHg.  Difference in means between Treatment and Control groups at final follow-up:   \| Author \| Difference \| 95% CI \| \| --- \| --- \| --- \| \| Liu 2015 \| -1.00 mmHg** \| -3.34-1.34 \| \| Mainenti 2009 \| -5.4 mmHg* \| -11.08-0.28 \| \| Monzani 2001 \| 3.80 mmHg* \| -2.42-10.02 \| \| Monzani 2004 \| -3.00 mmHg* \| -7.97-1.97 \| \| Nagasaki 2009 \| -0.10 mmHg** \| -4.22-4.02 \| \| Stott 2017 \| -0.10 mmHg* \| -1.5-1.3 \| \| Yazici 2004 \| -0.50 mmHg* \| -5.53-4.53 \| \| Zhao 2016 \| -0.13 mmHg* \| -2.42-2.16 \|   * Value is the difference in mean scores at final follow up between treatment and control groups.**Value is the difference in mean variation scores from baseline to follow-up between treatment and control group. | There is moderate certainty that no statistically significant difference exists between those treated and not treated for subclinical hypothyroidism on diastolic blood pressure. |
| Weight change - Body Mass Index follow up: range 5 months to 12 months | 1163 (11 RCTs) ^k^ | ⨁⨁⨁◯ MODERATE ^c,d,l,m^ | All but one study found no difference in body mass index (BMI) measurements between those treated and not treated for subclinical hypothyroidism. One study found that mean BMI measurements at follow-up were significantly higher in the treatment group than in the control group. Difference in means between treatment and control groups at follow-up ranged from -1.20 to 2.90 kg/m2.  Difference in means between Treatment and Control groups at final follow-up:   \| Author \| Sample size (treatment group vs. control group) \| Difference \| 95% CI \| \| --- \| --- \| --- \| --- \| \| Caraccio 2002 \| 49 (24 vs. 25) \| 1.30 kg/m^2^* \| -0.33-2.93 \| \| Caraccio 2005 \| 23 (12 vs. 11) \| -0.40 kg/m^2^* \| -2.52-1.72 \| \| Duman 2007 \| 39 (20 vs. 19) \| -0.70 kg/m^2^* \| -2.91-1.51 \| \| Iqbal 2006 \| 64 (32 vs. 32) \| 1.40 kg/m^2^* \| -1.06-3.86 \| \| Liu 2015 \| 119 (60 vs. 59) \| -0.10 kg/m^2^** \| -0.51-0.31 \| \| Monzani 2001 \| 20 (10 vs. 10) \| 0.00 kg/m^2^* \| -3.22-3.22 \| \| Monzani 2004 \| 45 (23 vs. 22) \| -1.20 kg/m^2^* \| -3.34-0.94 \| \| Nagasaki 2009 \| 95 (48 vs. 47) \| -0.30 kg/m^2^** \| -1.26-0.66 \| \| Stott 2017 \| 638 (318 vs. 320) \| 0.0 kg/m^2^* \| -0.02-0.02 \| \| Teixeira 2008 \| 26 (11 vs. 15) \| 2.90 kg/m^2^* \| 0.38-5.42 \| \| Yazici 2004 \| 45 (23 vs. 22) \| -0.20 kg/m^2^* \| -2.10-1.70 \|   * Value is the difference in mean scores at final follow up between treatment and control groups.**Value is the difference in mean variation scores from baseline to follow-up between treatment and control group. | There is moderate certainty that no statistically significant difference exists between those treated and not treated for subclinical hypothyroidism on body mass index. |
| ***The risk in the intervention group** (and its 95% confidence interval) is based on the assumed risk in the comparison group and the **relative effect** of the intervention (and its 95% CI).   **CI:** Confidence interval; **MD:** Mean difference | | | |  |
| **GRADE Working Group grades of evidence** **High certainty:** We are very confident that the true effect lies close to that of the estimate of the effect **Moderate certainty:** We are moderately confident in the effect estimate: The true effect is likely to be close to the estimate of the effect, but there is a possibility that it is substantially different **Low certainty:** Our confidence in the effect estimate is limited: The true effect may be substantially different from the estimate of the effect **Very low certainty:** We have very little confidence in the effect estimate: The true effect is likely to be substantially different from the estimate of effect | | | |  |

#### Explanations

a. Cabral 2011, Caraccio 2002, Duman 2007, Iqbal 2006, Liu 2015, Mikhail 2008, Monzani 2004, Nagasaki 2009, Teixeira 2008, Zhao 2016

b. Most of the information is from studies with unknown risk of bias for sequence generation and allocation concealment but low risk of bias for blinding of participants and personnel and outcome assessment. Because the outcome measure is based on an objective test (i.e. blood results), then we will downgrade by 0.5 point for risk of bias (and combined it with the 0.5 point from imprecision).

c. The individual point estimates are fairly close to each other and the majority of confidence intervals overlap. Therefore we will not rate down for inconsistency.

d. These findings are from studies addressing the key question on treatment. Although this is meant to provide linked evidence to inform the CTFPHC's recommendation on screening for thyroid dysfunction, indirectness of the evidence to the question on screening will be addressed in the Evidence-to-Decision framework. Therefore we will not rate down for indirectness in the GRADE tables.

e. The estimates likely do not cross a clinically important threshold, however the sample sizes across studies are fairly small therefore we will downgrade by 0.5 point. Combined with the 0.5 point downgrade from risk of bias, we downgraded by 1 for imprecision.

f. Cabral 2011, Caraccio 2002, Duman 2007, Iqbal 2006, Liu 2015, Mikhail 2008, Monzani 2004, Nagasaki 2009, Teixeira 2008

g. Liu 2015, Mainenti 2009, Monzani 2001, Monzani 2004, Nagasaki 2009, Stott 2017, Yazici 2004, Zhao 2016

h. Most of the studies are with unknown risk of bias for sequence generation and allocation concealment but low risk of bias for blinding of participants and personnel and outcome assessment. Because the outcome measure is based on an objective test (i.e. blood results) and because the studies contributing 60% of the weight for this estimate have low risk of bias for sequence generation and blinding of participants and personnel, then we will not downgrade for risk of bias.

i. Although most of the point estimates are fairly close to each other and most of the confidence intervals overlap, there are a few that are not. Therefore we will downgrade by 0.5 point and add to the 0.5 downgrade for imprecision.

j. Although most of the estimates likely do not cross a clinically important threshold, the sample sizes across studies are small. Therefore we will downgrade by 0.5 point. Combined with the 0.5 point from inconsistency, we will downgrade by 1 for imprecision.

k. Caraccio 2002, Caraccio 2005, Duman 2007, Iqbal 2006, Liu 2015, Monzani 2001, Monzani 2004, Nagasaki 2009, Stott 2017, Teixeira 2008, Yazici 2004

l. Most of the studies included in this meta-analysis are studies with unknown risk of bias for sequence generation and allocation concealment but low risk of bias for blinding of participants and personnel and outcome assessment. Because the outcome measure is based on an objective test (i.e. blood results) and because we do not believe that any uncertain biases (i.e. for sequence generation or allocation concealment) impacted the results. Therefore we will not downgrade for risk of bias.

m. Some estimates may cross a clinically important threshold and the sample sizes across studies are small. Therefore we will downgrade for imprecision.

**Table 2.2 GRADE Evidence Profile Table – intermediate outcomes**

**Question**: 3b. Does treatment of screen-detected overt or subclinical thyroid dysfunction improve intermediate outcomes?  Narrative

**Setting**: Primary care in Canada

| **Certainty assessment** | | | | | | | **Impact** | **Certainty** | **Importance** |
| --- | --- | --- | --- | --- | --- | --- | --- | --- | --- |
| **№ of studies** | **Study design** | **Risk of bias** | **Inconsistency** | **Indirectness** | **Imprecision** | **Other considerations** |  |  |  |
| Bone Mineral Density | | | | | | | | | |
| 0 |  |  |  |  |  |  |  | - | IMPORTANT |
| Total Cholesterol (follow up: range 5 months to 12 months) | | | | | | | | | |
| 10 ^a^ | randomised trials | not serious ^b^ | not serious ^c^ | not serious ^d^ | serious ^e^ | none | Results were mixed. Six studies did not find a statistically significant difference in total cholesterol (TC) levels between those treated and not treated for subclinical hypothyroidism. Three studies found that mean values for TC levels at final follow-up were less in the treatment group compared to the control group and the findings were statistically significant. One study found the decline in mean TC levels from baseline to follow-up was statistically significantly larger in the treatment group than in the control group. Difference in means between treatment and control groups at final follow-up ranged from -1.07 to 0.00 mmol/L. | ⨁⨁⨁◯ MODERATE | IMPORTANT |
| Low-density Lipoprotein (follow up: range 5 months to 12 months) | | | | | | | | | |
| 9 ^f^ | randomised trials | not serious ^b^ | not serious ^c^ | not serious ^d^ | serious ^e^ | none | The majority of studies (8/9) did not find a difference in low-density lipoprotein (LDL) levels between those treated and not treated for subclinical hypothyroidism. Difference in means between treatment and control groups at final follow-up ranged from -1.23 to 0.11 mmol/L. | ⨁⨁⨁◯ MODERATE | IMPORTANT |
| High-density Lipoprotein (follow up: range 5 months to 12 months) | | | | | | | | | |
| 9 ^f^ | randomised trials | not serious ^b^ | not serious ^c^ | not serious ^d^ | serious ^e^ | none | No studies found a difference in high-density lipoprotein (HDL) levels between those treated and not treated for subclinical hypothyroidism. Difference in means between treatment and control groups at final follow-up ranged from -0.17 to 0.26 mmol/L. | ⨁⨁⨁◯ MODERATE | IMPORTANT |
| Triglycerides (follow up: range 5 months to 12 months) | | | | | | | | | |
| 10 ^a^ | randomised trials | not serious ^b^ | not serious ^c^ | not serious ^d^ | serious ^e^ | none | All but one study found no difference in triglyceride levels between those treated and not treated for subclinical hypothyroidism. Difference in means between treatment and control groups at final follow-up ranged from -1.72 to 0.12 mmol/L. | ⨁⨁⨁◯ MODERATE | IMPORTANT |
| Systolic Blood Pressure (follow up: range 5 months to 15 months) | | | | | | | | | |
| 8 ^g^ | randomised trials | not serious ^h^ | not serious ^i^ | not serious ^d^ | serious ^j^ | none | No studies found a difference in systolic blood pressure measurements at final follow-up between those treated and not treated for subclinical hypothyroidism. Difference in means between treatment and control groups at final follow-up ranged from -12.25 to 0.5 mmHg. | ⨁⨁⨁◯ MODERATE | IMPORTANT |
| Diastolic Blood Pressure (follow up: range 5 months to 15 months) | | | | | | | | | |
| 8 ^g^ | randomised trials | not serious ^h^ | not serious ^i^ | not serious ^d^ | serious ^j^ | none | No studies found a difference in diastolic blood pressure measurements at final follow-up between those treated and not treated for subclinical hypothyroidism. Difference in means between treatment and control groups at follow-up ranged from -5.4 to 3.8 mmHg. | ⨁⨁⨁◯ MODERATE | IMPORTANT |
| Weight change - Body Mass Index (follow up: range 5 months to 12 months) | | | | | | | | | |
| 11 ^k^ | randomised trials | not serious ^l^ | not serious ^c^ | not serious ^d^ | serious ^m^ | none | All but one study found no difference in body mass index (BMI) measurements between those treated and not treated for subclinical hypothyroidism. One study found that mean BMI measurements at follow-up were significantly higher in the treatment group than in the control group. Difference in means between treatment and control groups at follow-up ranged from -1.20 to 2.90 kg/m2. | ⨁⨁⨁◯ MODERATE | IMPORTANT |

**CI:** Confidence interval; **MD:** Mean difference

#### Explanations

a. Cabral 2011, Caraccio 2002, Duman 2007, Iqbal 2006, Liu 2015, Mikhail 2008, Monzani 2004, Nagasaki 2009, Teixeira 2008, Zhao 2016

b. Most of the information are from studies with unknown risk of bias for sequence generation and allocation concealment but low risk of bias for blinding of participants and personnel and outcome assessment. Because the outcome measure is based on an objective test (i.e. blood results), then we will downgrade by 0.5 point for risk of bias (and combined it with the 0.5 point from imprecision).

c. The individual point estimates are fairly close to each other and the majority of confidence intervals overlap. Therefore we will not rate down for inconsistency.

d. These findings are from studies addressing the key question on treatment. Although this is meant to provide linked evidence to inform the CTFPHC's recommendation on screening for thyroid dysfunction, indirectness of the evidence to the question on screening will be addressed in the Evidence-to-Decision framework. Therefore we will not rate down for indirectness in the GRADE tables.

e. The estimates likely do not cross a clinically important threshold, however the sample sizes across studies are fairly small therefore we will downgrade by 0.5 point. Combined with the 0.5 point downgrade from risk of bias, we downgraded by 1 for imprecision.

f. Cabral 2011, Caraccio 2002, Duman 2007, Iqbal 2006, Liu 2015, Mikhail 2008, Monzani 2004, Nagasaki 2009, Teixeira 2008

g. Liu 2015, Mainenti 2009, Monzani 2001, Monzani 2004, Nagasaki 2009, Stott 2017, Yazici 2004, Zhao 2016

h. Most of the studies are with unknown risk of bias for sequence generation and allocation concealment but low risk of bias for blinding of participants and personnel and outcome assessment. Because the outcome measure is based on an objective test (i.e. blood results) and because the studies contributing 60% of the weight for this estimate have low risk of bias for sequence generation and blinding of participants and personnel, then we will not downgrade for risk of bias.

i. Although most of the point estimates are fairly close to each other and most of the confidence intervals overlap, there are a few that are not. Therefore we will downgrade by 0.5 point and add to the 0.5 downgrade for imprecision.

j. Although most of the estimates likely do not cross a clinically important threshold, the sample sizes across studies are small. Therefore we will downgrade by 0.5 point. Combined with the 0.5 point from inconsistency, we will downgrade by 1 for imprecision.

k. Caraccio 2002, Caraccio 2005, Duman 2007, Iqbal 2006, Liu 2015, Monzani 2001, Monzani 2004, Nagasaki 2009, Stott 2017, Teixeira 2008, Yazici 2004

l. Most of the studies included in this meta-analysis are studies with unknown risk of bias for sequence generation and allocation concealment but low risk of bias for blinding of participants and personnel and outcome assessment. Because the outcome measure is based on an objective test (i.e. blood results) and because we do not believe that any uncertain biases (i.e. for sequence generation or allocation concealment) impacted the results. Therefore we will not downgrade for risk of bias.

m. Some estimates may cross a clinically important threshold and the sample sizes across studies are small. Therefore we will downgrade for imprecision.

**Evidence Set 3**

**KQ4: What are the harms of treating screen-detected thyroid dysfunction in asymptomatic, non-pregnant adults?**

**Table 3.1 GRADE Summary of Findings Table – harms**

| **4. What are the harms of treatment of screen-detected thyroid dysfunction?** | | | |  |
| --- | --- | --- | --- | --- |
| **Outcomes Follow-up** | **№ of participants (№ of studies)** | **Certainty of the evidence (GRADE)** | **Impact** | **Comments** |
| Number of individuals reporting adverse outcomes follow up: range 48 weeks to 3 years | 1370 (5 RCTs) ^a^ | ⨁⨁◯◯ LOW ^b,c,d,e^ | Two studies did not find a statistically significant difference in the odds of reporting adverse outcomes at final follow-up between those treated and not treated for subclinical hypothyroidism but one study found the odds of reporting adverse outcomes were higher in the treatment group than in the control group (OR of 21.87 [95% CI: 1.25-383.87]). The odds ratios could not be calculated for the other studies due to missing data or because no adverse outcomes were reported in both the treatment and control groups. The proportion of adverse outcomes reported in the control groups ranged from 0%-8.2% and the range was 0%-26.3% in the treatment group.   \| Author \| Results \| Descriptive reports of adverse outcomes (results continued) \| \| --- \| --- \| --- \| \| Fadeyev 2006 \| Treatment group: 5 events; 26.3% \| In the treatment group 5/19 patients had various adverse events during treatment [3 patients had more ventricular premature beats (VPB) and 2 patients had an increased mean heart rate in conjunction with an increased number of VPB]. At the end of the follow-up period, one of the patients in the treatment group had an unstable episode of ventricular tachycardia.  * AEs in the control group were not reported. \| \| Liu 2015 \| Control group (0 events; 0.0%) vs treatment group (9 events; 13.2%) \| OR: 21.87 (95% CI: 1.25-383.87)  Absolute value (range): 0 fewer per 1,000 (from 0 fewer to 0 fewer)  Adverse reactions included mild insomnia, mild diarrhea, mild paroxysmal supraventricular tachycardia, and palpitations. \| \| Nagasaki 2009 \| Control group (0 events; 0.0%) vs treatment group (0 events; 0.0%) \| None of the patients experienced side effects such as arrhythmia, angina pectoris, or hypertension that would have required withdrawal or reduction of the dose of levothyroxine. \| \| Stott 2017 \| Control group (103 events, 27.9%) vs treatment group (78 events, 21.2%) \| HR of 0.94 (95% CI of 0.88-1.0)  Absolute value (range): 14 fewer per 1,000 (from 0 fewer to 29 fewer)  * for Serious Adverse Events only \| \| Zhao 2016 \| Control group (13 events; 8.2%) vs treatment group (16 events; 7.6%) \| OR: 0.93 (95% CI: 0.43-1.99)  Absolute value (range): 5 fewer per 1,000 (from 45 fewer to 69 more)  Adverse symptoms included palpitations, chest tightness, dizziness, perspiration, low back pain, and hunched back. No participant attempted to visit a physician due to adverse effects. \| | There may be no statistically significant difference between those treated and not treated for subclinical hypothyroidism on the number of individuals reporting adverse outcomes. |
| Withdrawal due to adverse outcomes follow up: range 48 weeks to 12 months | 290 (4 RCTs) ^f^ | ⨁⨁◯◯ LOW ^d,e,g,h^ | Three studies did not find a statistically significant difference in the odds of withdrawing from the study due to an adverse outcome between those treated and not treated for subclinical hypothyroidism. The odds ratios could not be calculated for the other study because no adverse outcomes were reported in both the treatment and control groups. The proportion of withdrawals due to adverse outcomes reported in the control groups ranged from 0%-14.3% and the range was 0%-9.6% in the treatment group.   \| Author \| Results \| Descriptive reports of adverse outcomes (results continued) \| \| --- \| --- \| --- \| \| Liu 2015 \| Control group (0; 0.0% vs treatment group (1; 1.5%) \| OR: 3.04 (95% CI: 0.12-76.06)  Absolute value (range): 0 fewer per 1,000 (from 0 fewer to 0 fewer)  Adverse reaction requiring withdrawal from the study was mild paroxysmal supraventricular tachycardia. \| \| Parle 2010 \| Control group (6; 14.3% vs treatment group (5; 9.6%) \| OR: 0.64 (95% CI: 0.18-2.26)  Absolute value (range): 46 fewer per 1,000 (from 114 fewer to 131 more)  The side effects resulting in withdrawal from the trial were not described. \| \| Teixeira 2008 \| Control group (0; 0.0% vs treatment group (2; 5.7%) \| OR: 3.81 (95% CI: 0.17-82.80)  Absolute value (range): 0 fewer per 1,000 (from 0 fewer to 0 fewer)  Adverse events requiring withdrawal from the study included developing "hashitoxicosis" while on levothyroxine therapy and symptomatic tachycardia. \| \| Zhao 2016 \| Control group (0; 0.0%) vs treatment group (0; 0.0%) \| No withdrawals due to adverse effects were reported in either the control or treatment groups. \| | There may be no statistically significant difference between those treated and not treated for subclinical hypothyroidism on withdrawals from a study due to adverse outcomes. |
| ***The risk in the intervention group** (and its 95% confidence interval) is based on the assumed risk in the comparison group and the **relative effect** of the intervention (and its 95% CI).   **CI:** Confidence interval; **OR:** Odds ratio | | | |  |
| **GRADE Working Group grades of evidence** **High certainty:** We are very confident that the true effect lies close to that of the estimate of the effect **Moderate certainty:** We are moderately confident in the effect estimate: The true effect is likely to be close to the estimate of the effect, but there is a possibility that it is substantially different **Low certainty:** Our confidence in the effect estimate is limited: The true effect may be substantially different from the estimate of the effect **Very low certainty:** We have very little confidence in the effect estimate: The true effect is likely to be substantially different from the estimate of effect | | | |  |

#### Explanations

a. Fadeyev 2006, Liu 2015, Nagasaki 2009, Stott 2017, Zhao 2016

b. Although some of the information are from studies with uncertain or high risk of bias for allocation concealment, blinding of participants, personnel and outcome assessment, the study providing the most weight (by sample size) for this outcome was of low risk of bias. In addition, we believe that the bias did not materialize based on the findings. Therefore we will not downgrade for risk of bias.

c. Point estimates and confidence intervals were not available for most of the studies due to low number of events. The methods for monitoring and reporting adverse events may have varied by study. The proportion of participants reporting adverse events varied across studies therefore we will downgrade for inconsistency.

d. These findings are from studies addressing the key question on treatment. Although this is meant to provide linked evidence to inform the CTFPHC's recommendation on screening for thyroid dysfunction, indirectness of the evidence to the question on screening will be addressed in the Evidence-to-Decision framework. Therefore we will not rate down for indirectness in the GRADE tables.

e. No clinically meaningful threshold was set and the sample size across studies was small. Therefore we will downgrade for imprecision.

f. Liu 2015, Parle 2010, Teixeira 2008, Zhao 2016

g. Although there were some uncertain or high risk of bias assessments for sequence generation, allocation concealment and blinding of participants, personnel and outcome assessment, we believe that the bias did not materialize based on the findings. Therefore we will not downgrade for risk of bias.

h. Point estimates and confidence intervals were not available for most of the studies due to low number of events. The methods for monitoring and reporting adverse events and subsequent withdrawals due to adverse events may have varied by study. The proportion of withdrawals due to adverse events varied across studies therefore we will downgrade for inconsistency.

**Table 3.2 GRADE Evidence Profile Table – harms**

**Question**: 4. What are the harms of treatment of screen-detected thyroid dysfunction?

**Setting**: Primary Care in Canada

| **Certainty assessment** | | | | | | | **Impact** | **Certainty** | **Importance** |
| --- | --- | --- | --- | --- | --- | --- | --- | --- | --- |
| **№ of studies** | **Study design** | **Risk of bias** | **Inconsistency** | **Indirectness** | **Imprecision** | **Other considerations** |  |  |  |
| Number of individuals reporting adverse outcomes (follow up: range 48 weeks to 3 years) | | | | | | | | | |
| 5 ^a^ | randomised trials | not serious ^b^ | serious ^c^ | not serious ^d^ | serious ^e^ | none | Two RCTs found that the odds of reporting an adverse outcome did not differ between the treatment and control groups. One RCT found that the odds of reporting an adverse outcome was statistically significantly higher in the treatment group than in the control group. Odds ratios could not be calculated for the other studies due to small number of events. The proportion of adverse outcomes reported in the control groups ranged from 0%-8.2% and the range was 0%-26.3% in the treatment group. | ⨁⨁◯◯ LOW | CRITICAL |
| Withdrawal due to adverse outcomes (follow up: range 48 weeks to 12 months) | | | | | | | | | |
| 4 ^f^ | randomised trials | not serious ^g^ | serious ^h^ | not serious ^d^ | serious ^e^ | none | Three RCTs found that the odds of withdrawing from the study due to an adverse outcome did not differ between the treatment and control groups. Odds ratios could not be calculated for the other study because no adverse outcomes were reported in both the treatment and control groups. The proportion of withdrawals due to adverse outcomes reported in the control groups ranged from 0%-14.3% and the range was 0%-9.6% in the treatment group. | ⨁⨁◯◯ LOW | CRITICAL |

**CI:** Confidence interval; **OR:** Odds ratio

#### Explanations

a. Fadeyev 2006, Liu 2015, Nagasaki 2009, Stott 2017, Zhao 2016

b. Although some of the information are from studies with uncertain or high risk of bias for allocation concealment, blinding of participants, personnel and outcome assessment, the study providing the most weight (by sample size) for this outcome was of low risk of bias. In addition, we believe that the bias did not materialize based on the findings. Therefore we will not downgrade for risk of bias.

c. Point estimates and confidence intervals were not available for most of the studies due to low number of events. The methods for monitoring and reporting adverse events may have varied by study. The proportion of participants reporting adverse events varied across studies therefore we will downgrade for inconsistency.

d. These findings are from studies addressing the key question on treatment. Although this is meant to provide linked evidence to inform the CTFPHC's recommendation on screening for thyroid dysfunction, indirectness of the evidence to the question on screening will be addressed in the Evidence-to-Decision framework. Therefore we will not rate down for indirectness in the GRADE tables.

e. No clinically meaningful threshold was set and the sample size across studies was small. Therefore we will downgrade for imprecision.

f. Liu 2015, Parle 2010, Teixeira 2008, Zhao 2016

g. Although there were some uncertain or high risk of bias assessments for sequence generation, allocation concealment and blinding of participants, personnel and outcome assessment, we believe that the bias did not materialize based on the findings. Therefore we will not downgrade for risk of bias.

h. Point estimates and confidence intervals were not available for most of the studies due to low number of events. The methods for monitoring and reporting adverse events and subsequent withdrawals due to adverse events may have varied by study. The proportion of withdrawals due to adverse events varied across studies therefore we will downgrade for inconsistency.

1. Represents the total number of participants in the study because n for the outcome was not provided in the published paper. [↑](#footnote-ref-1)
2. Represents the total number of female or male participants in the study because n for the outcome was not provided in the published paper [↑](#footnote-ref-2)
